# Supplementary material for: Synthesis and Characterization of 1,10-Phenanthroline-mono-N-oxides
Source: Molecules. 2021 Jun 14;26(12):3632. doi: 10.3390/molecules26123632 (PMC8231831; doi:10.3390/molecules26123632)
Supplement: Supplementary file 1 [file molecules-26-03632-s001.zip › molecules-1249202-supplementary.pdf]

Supplementary Material for

Synthesis and characterization of 1,10-phenanthroline-mono-*N*-oxides

Ferenc Najóczki[1, 4], Mária Szabó[1], Norbert Lihi[1,2], Antal Udvardy[3] and István Fábián[1,2]*

[1] Department of Inorganic and Analytical Chemistry, University of Debrecen, H-4032 Debrecen, Egyetem tér 1, Hungary

[2] MTA-DE Homogeneous Catalysis and Reaction Mechanisms Research Group, University of Debrecen, H-4032 Debrecen, Egyetem tér 1, Hungary

[3] Department of Physical Chemistry, University of Debrecen, H-4032 Debrecen, Egyetem tér 1, Hungary

[4] Doctoral School of Chemistry, University of Debrecen, H-4032 Debrecen, Egyetem tér 1., Hungary

Corresponding author: István Fábián, e-mail: ifabian@science.unideb.hu, Tel: + 36 52 512-900/22378, Fax: + 36 52 518-660

Table of Contents

General Information.. 3

Figure S1. 3

Copies of NMR and mass spectra 4

The acid dissociation constants of substituted mono-*N*-oxide derivatives 41

X-ray structures of 1,10-phenatroline-1-*N*-oxide derivatives 42

DFT and TD-DFT calculations.. 56

General Information

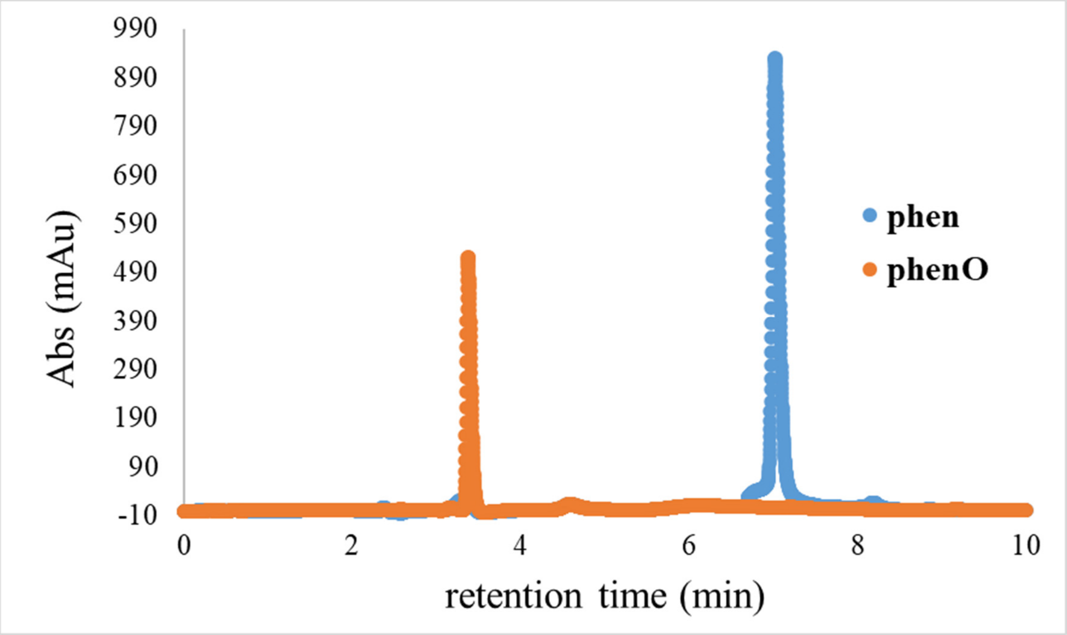

Figure S1. HPLC chromatogram recorded in the phen/PMS system after the extraction with $CHCl_3$ (orange). Blue: chromatogram of 1,10-phenanthroline. The elution was achieved on Phenomenex Luna 5u C18(2) 100A 250 x 4.60 mm 5 microns column. The chromatograms were detected at 230 nm.

Copies of NMR and mass spectra

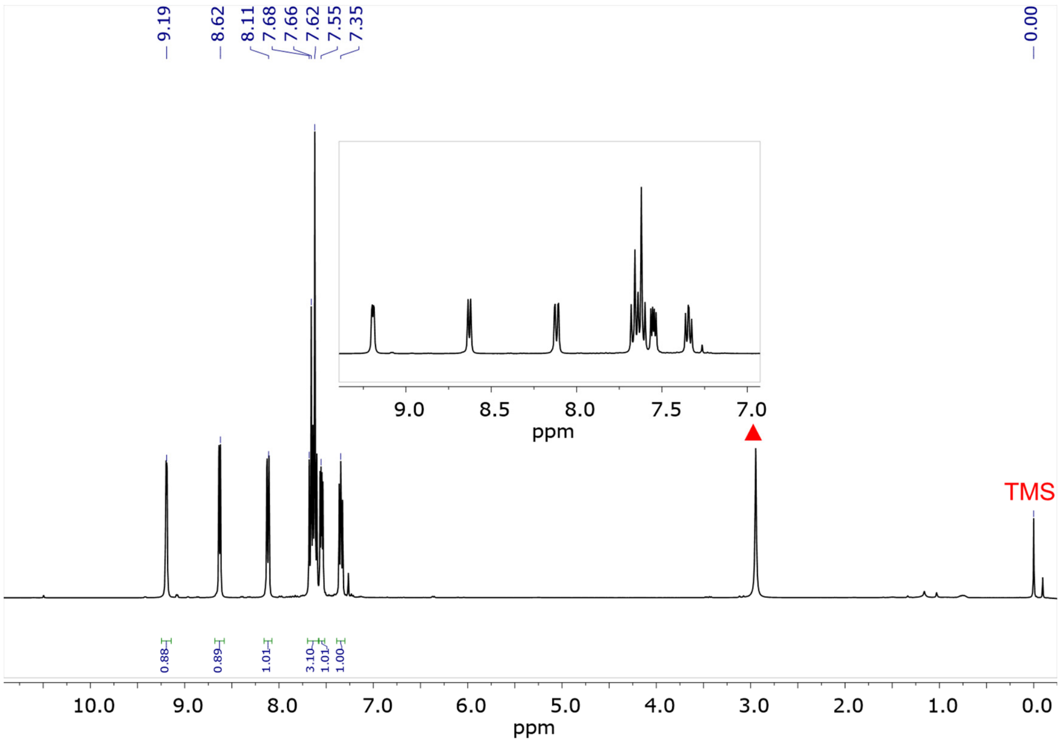

Figure S2. ^{1}H NMR spectrum of phenO in $CDCl_3$ (400 MHz, 25 °C). ▲ indicates solvent impurity. Inset: Selected region of the aromatic part.

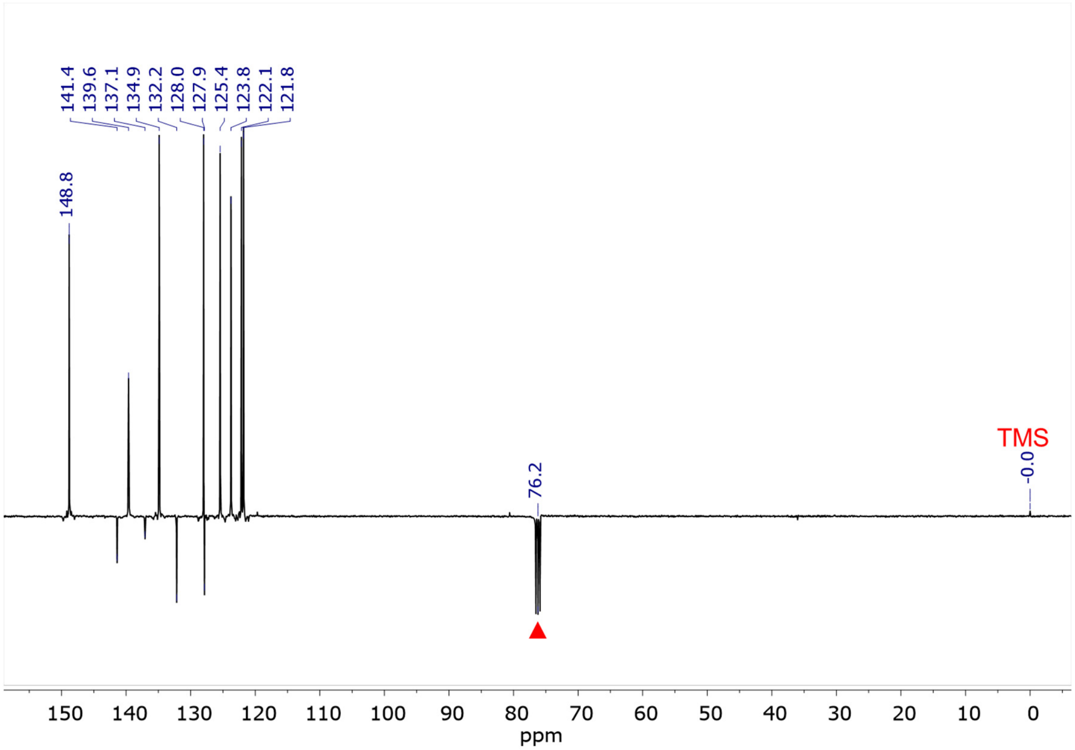

Figure S3. APT ^{13}C NMR spectrum of phenO in $CDCl_3$ (100.6 MHz, 25 °C). ▲ indicates the solvent residual peak.

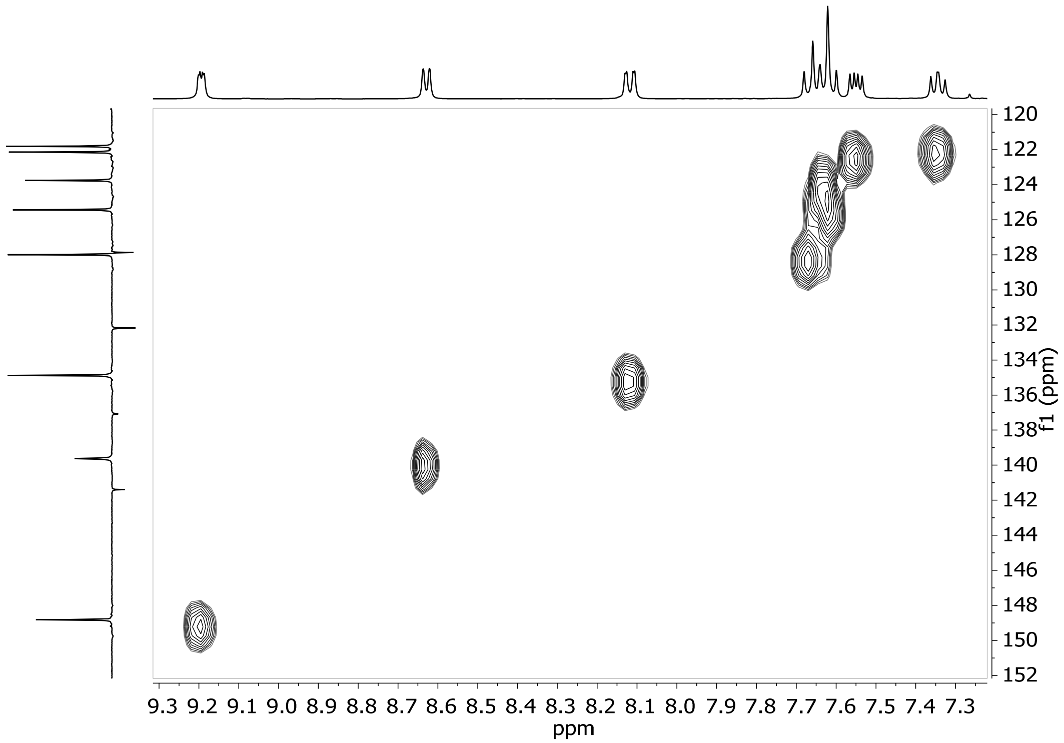

Figure S4. (^{1}H–^{13}C) HSQC NMR spectrum of phenO in $CDCl_3$ (400 MHz, 25 °C).

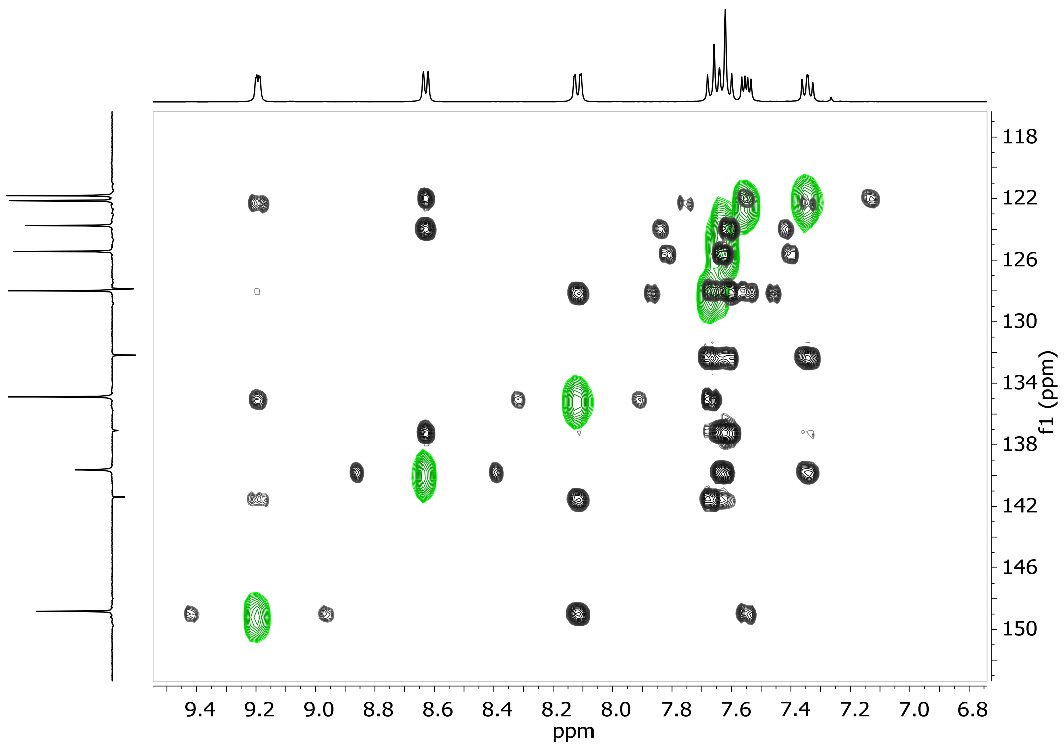

Figure S5. Superposition of the (^{1}H–^{13}C) HSQC (green) and (^{1}H–^{13}C) HMBC (black) NMR spectra of phenO in $CDCl_3$ (400 MHz, 25 °C).

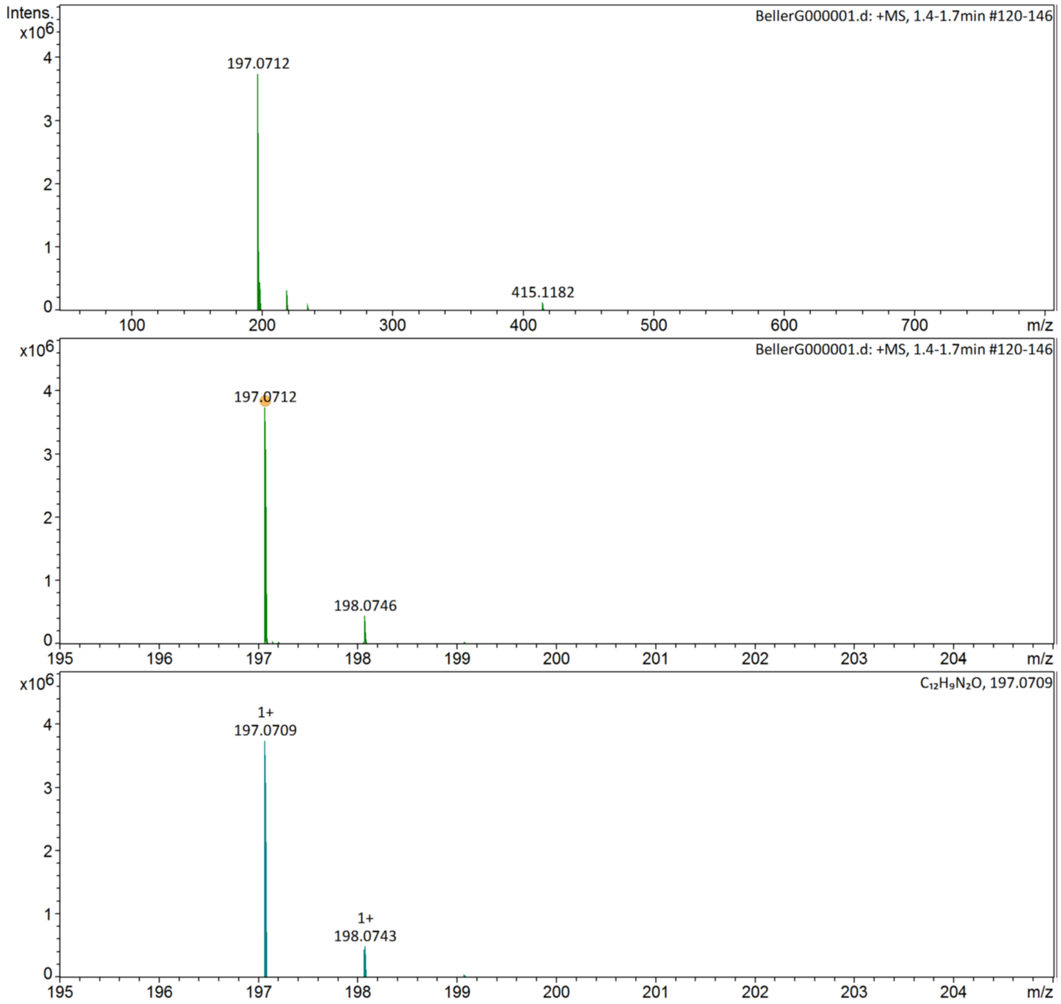

Figure S6. HRMS spectrum of phenO in positive mode.

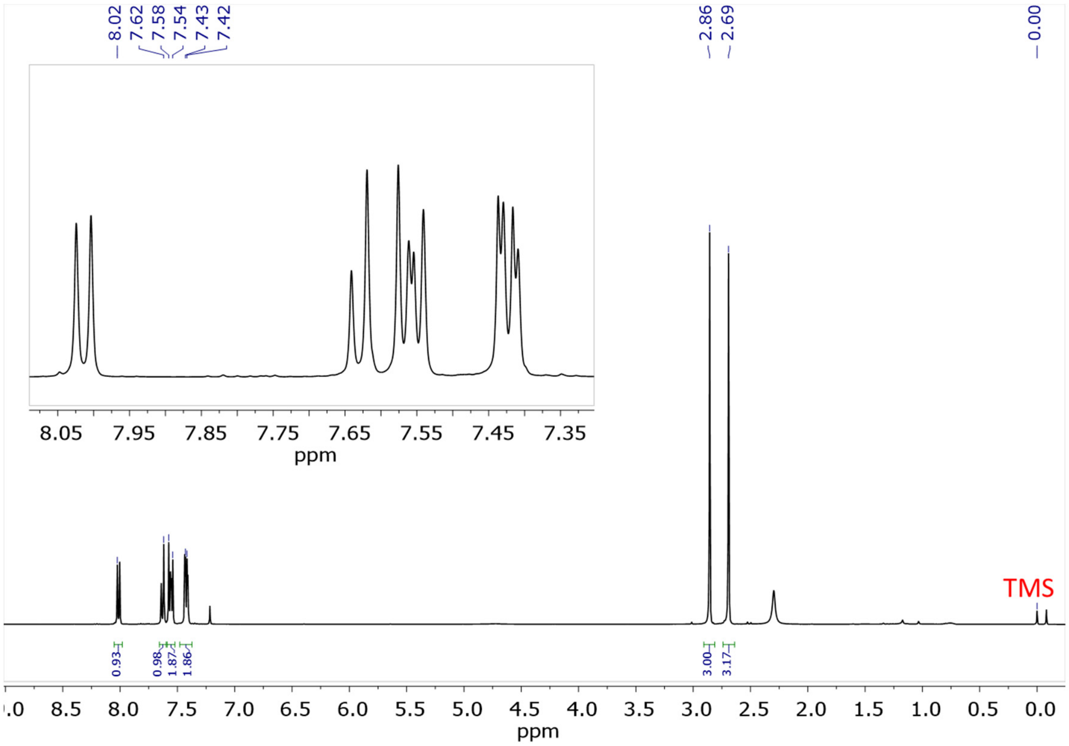

Figure S7. ^{1}H NMR spectrum of DMPO in $CDCl_3$ (400 MHz, 25 °C). Inset: Selected region of the aromatic part.

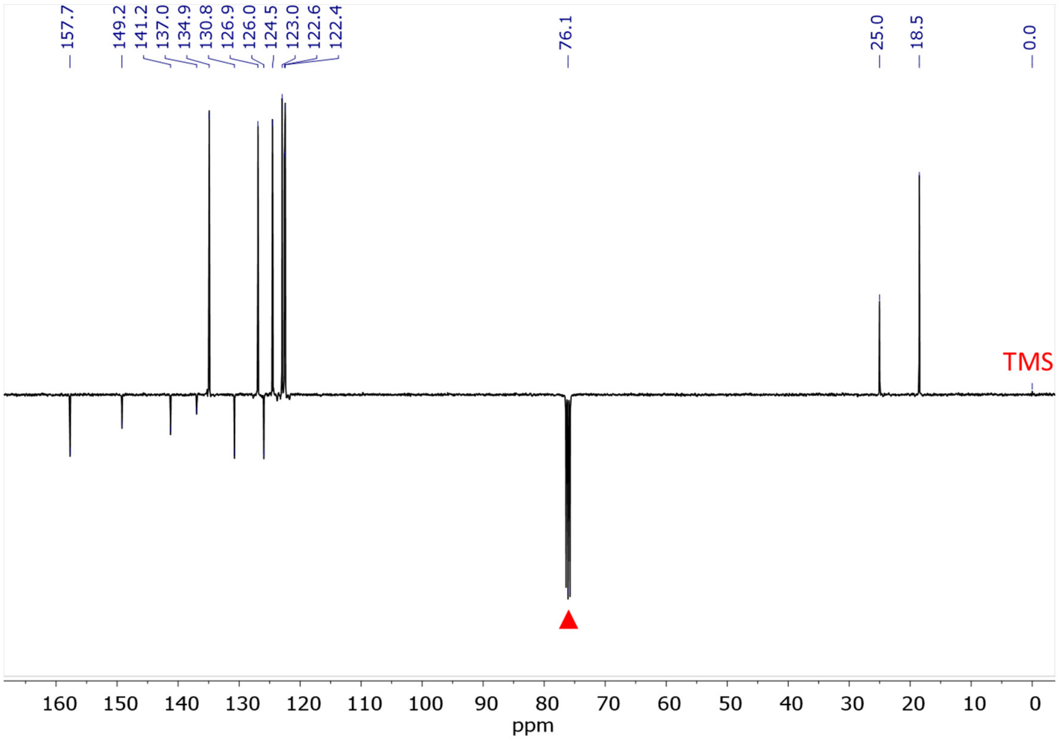

Figure S8. APT ^{13}C NMR spectrum of DMPO in $CDCl_3$ (100.6 MHz, 25 °C). ▲ indicates the solvent residual peak.

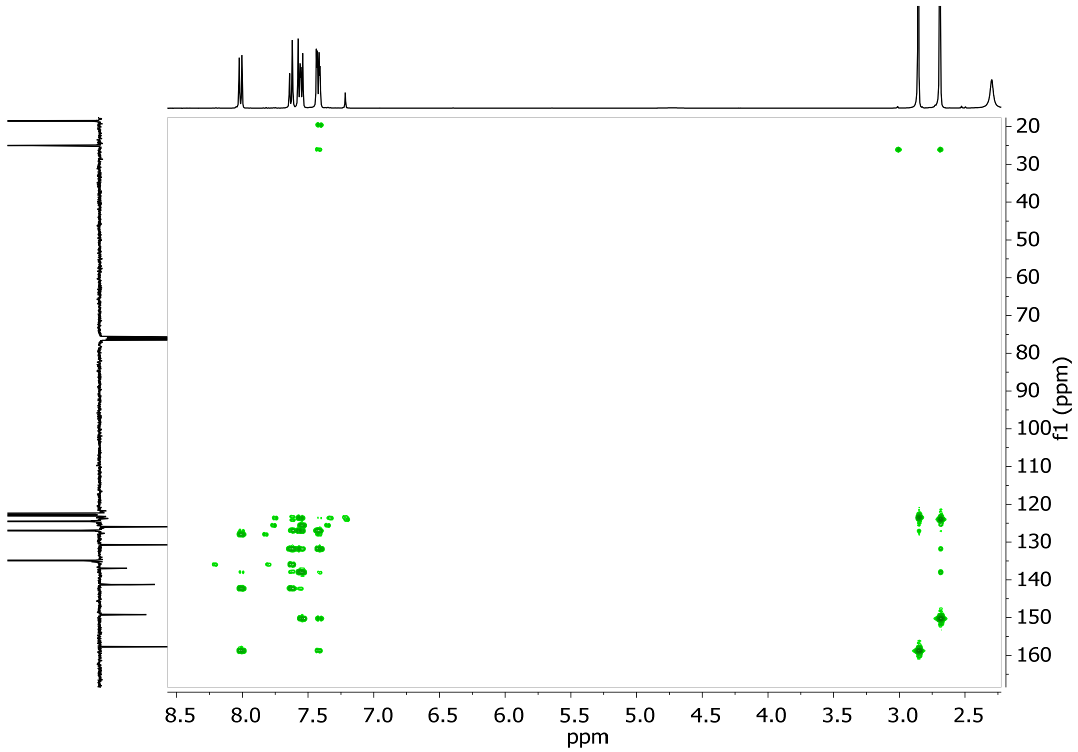

Figure S9. (^{1}H–^{13}C) HMBC NMR spectrum of DMPO in $CDCl_3$ (400 MHz, 25 °C).

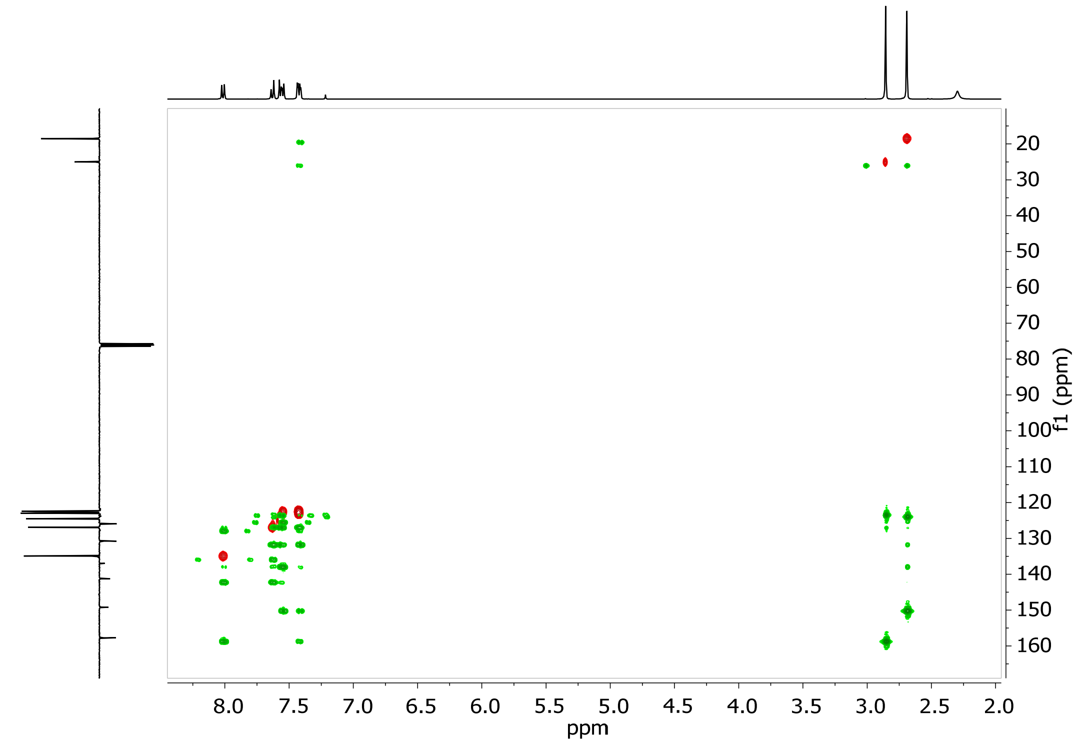

Figure S10. Superposition of the (^{1}H–^{13}C) HSQC (red) and (^{1}H–^{13}C) HMBC (green) NMR spectra of DMPO in $CDCl_3$ (400 MHz, 25 °C).

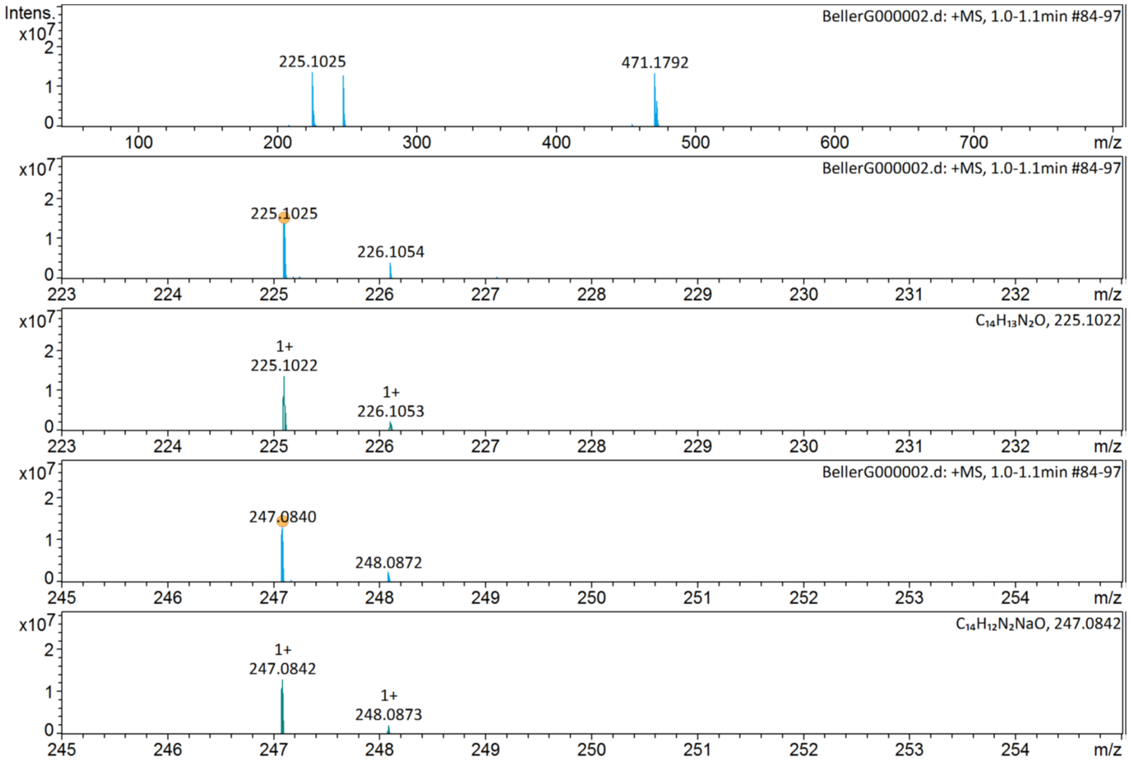

Figure S11. HRMS spectrum of DMPO in positive mode.

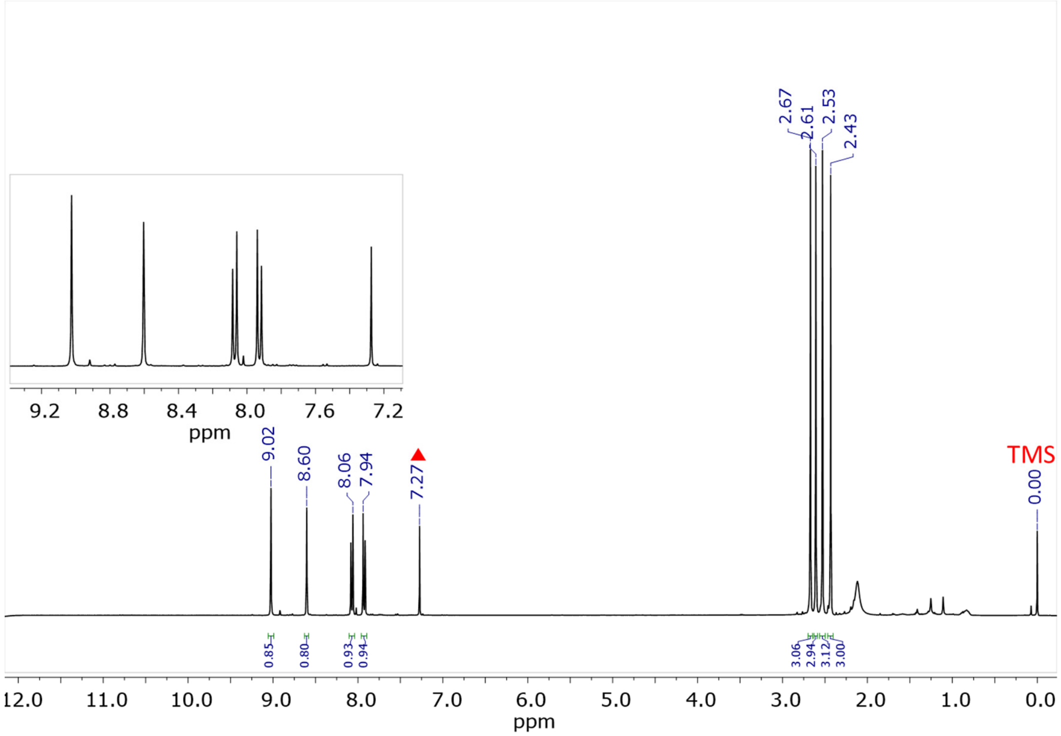

Figure S12. ^{1}H NMR spectrum of TMPO in $CDCl_3$ (400 MHz, 25 °C). ▲ indicates solvent residual peak. Inset: Selected region of the aromatic part.

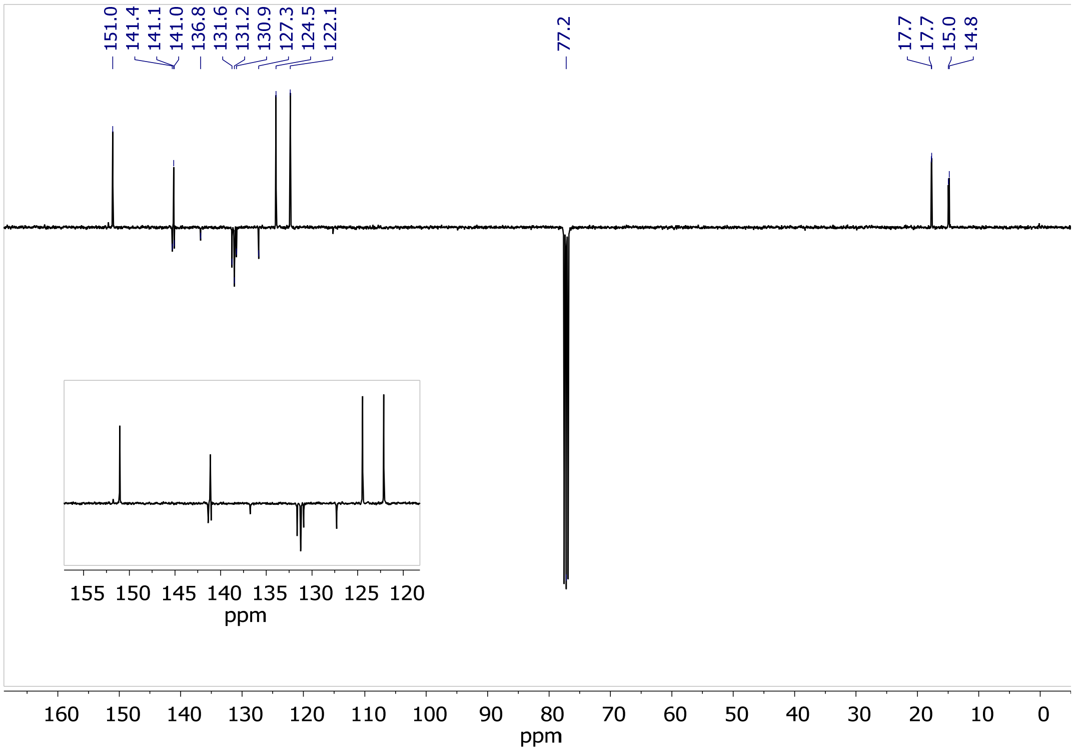

Figure S13. APT ^{13}C NMR spectrum of TMPO in $CDCl_3$ (100.6 MHz, 25 °C). ▲ indicates the solvent residual peak. Inset: Selected region of the aromatic part.

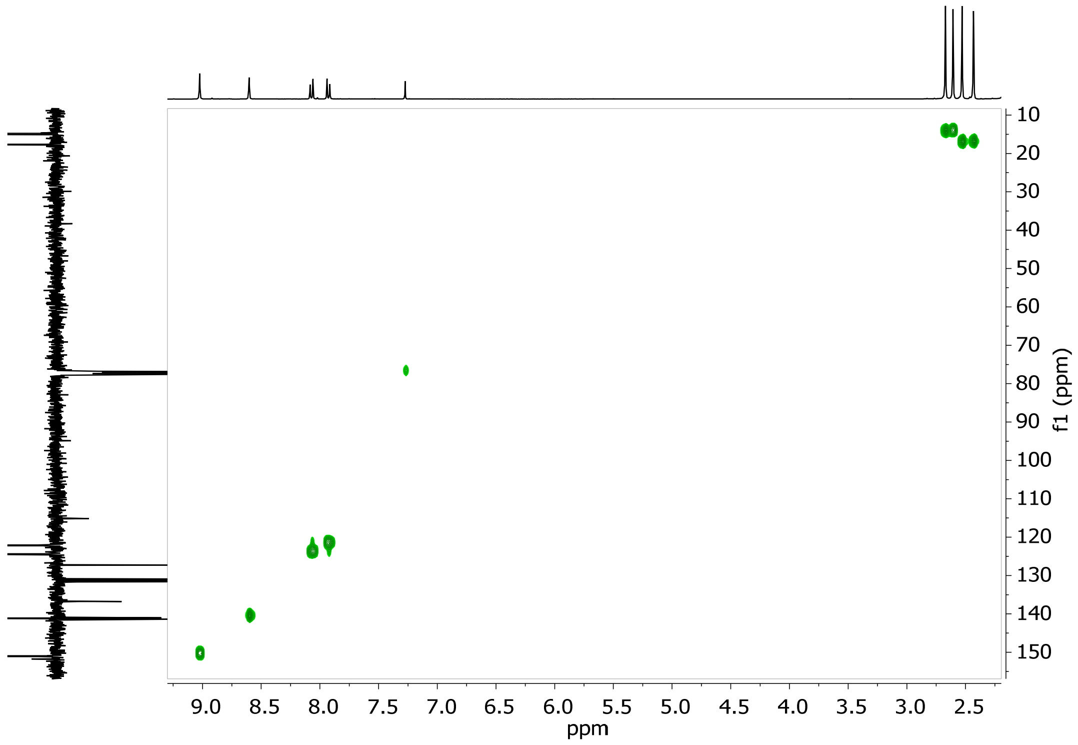

Figure S14. (^1H–^{13}C) HSQC NMR spectrum of TMPO in $CDCl_3$ (400 MHz, 25 °C).

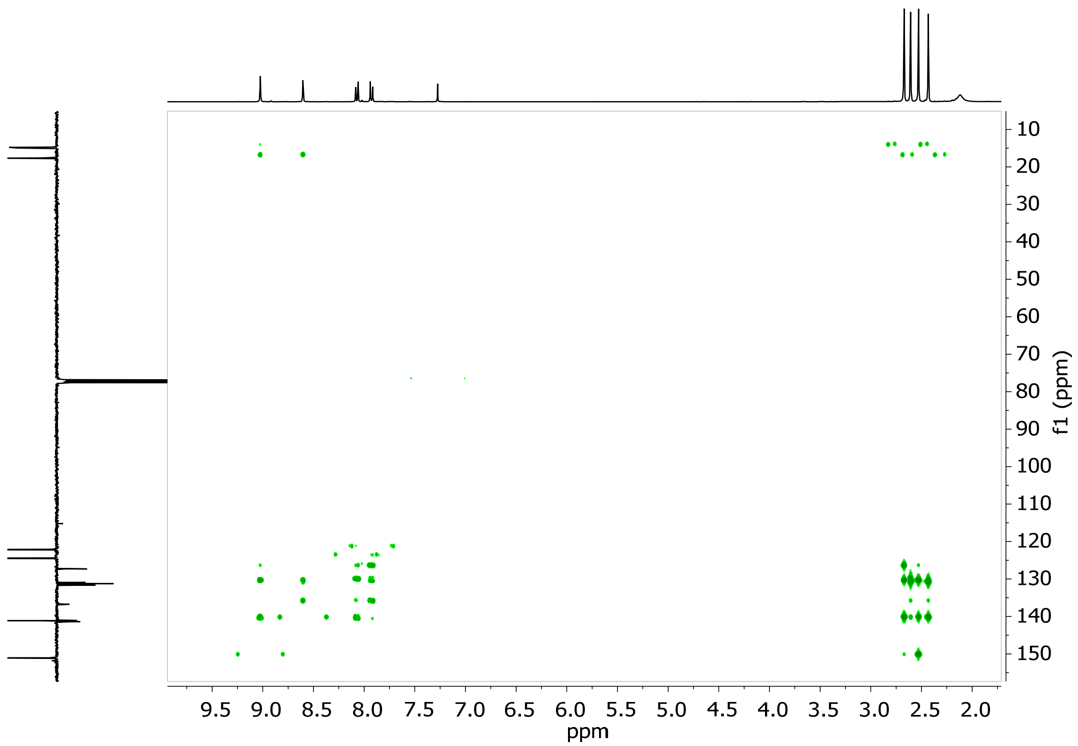

Figure S15. (^1H–^{13}C) HMBC NMR spectrum of TMPO in $CDCl_3$ (400 MHz, 25 °C).

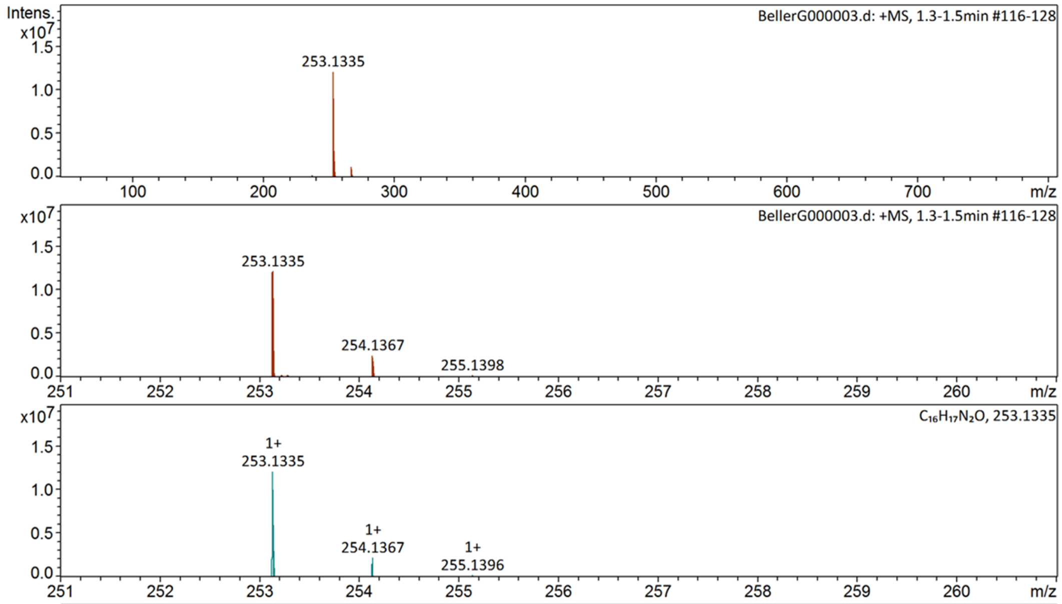

Figure S16. HRMS spectrum of TMPO in positive mode.

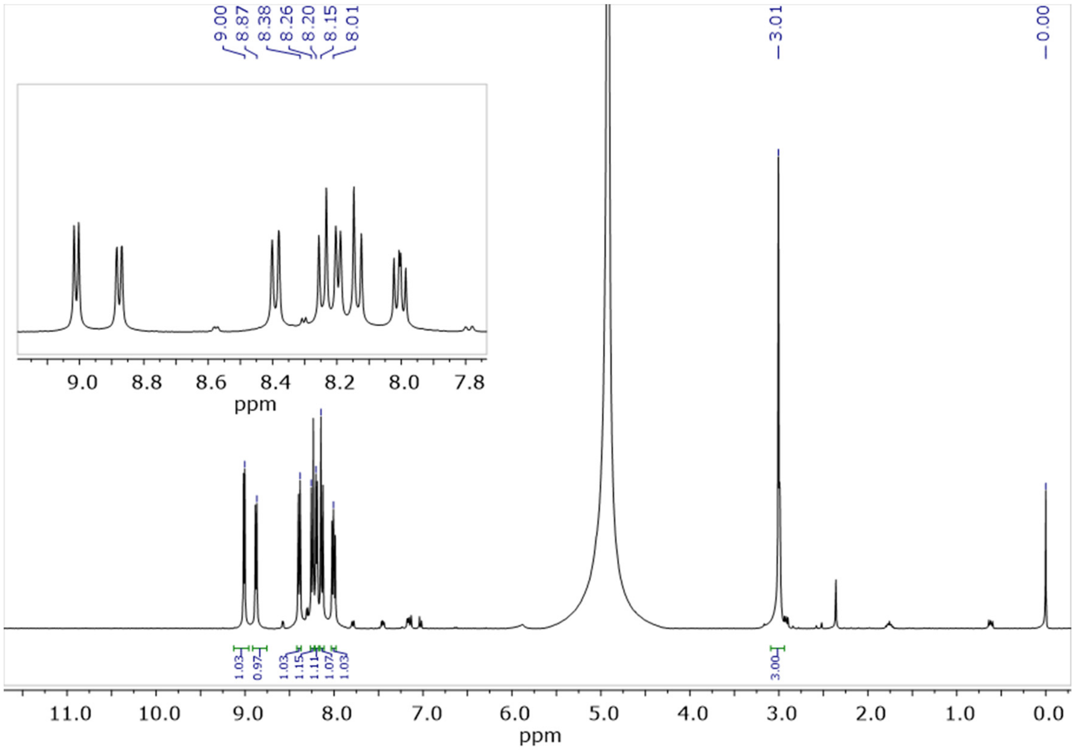

Figure S17. ^{1}H NMR spectrum of 4MPO in D_2O (400 MHz, 25 °C). Inset: Selected region of the aromatic part.

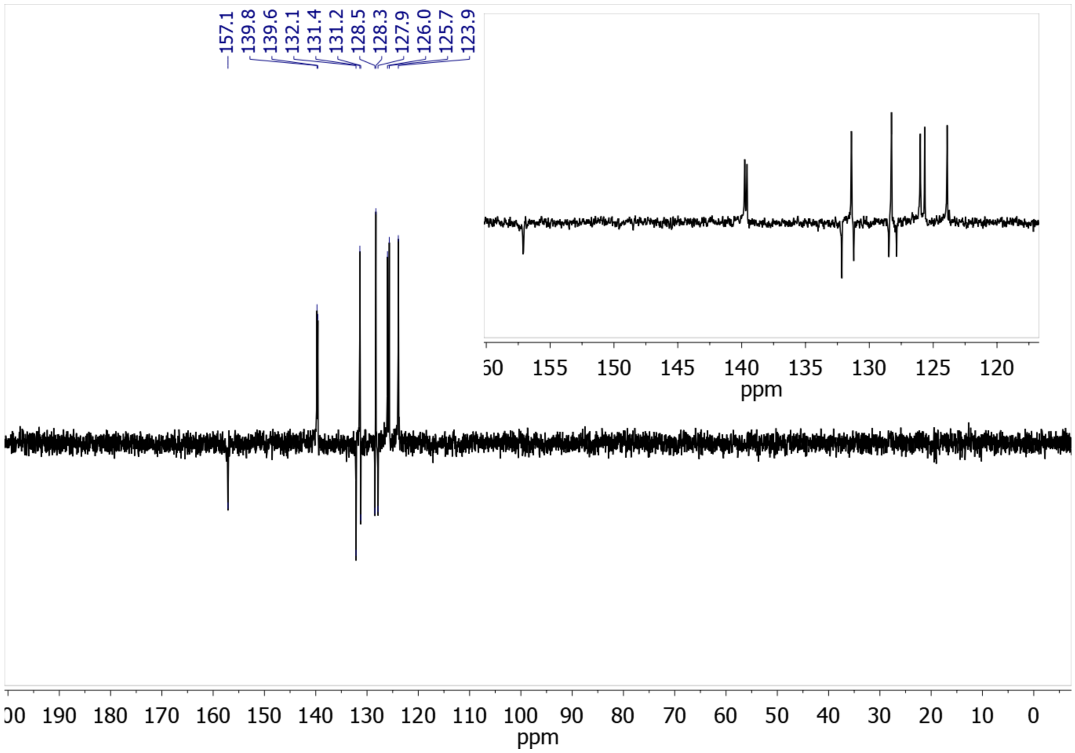

Figure S18. APT ^{13}C NMR spectrum of 4MPO in D_2O (100.6 MHz, 25 °C). Inset: Selected region of the aromatic part.

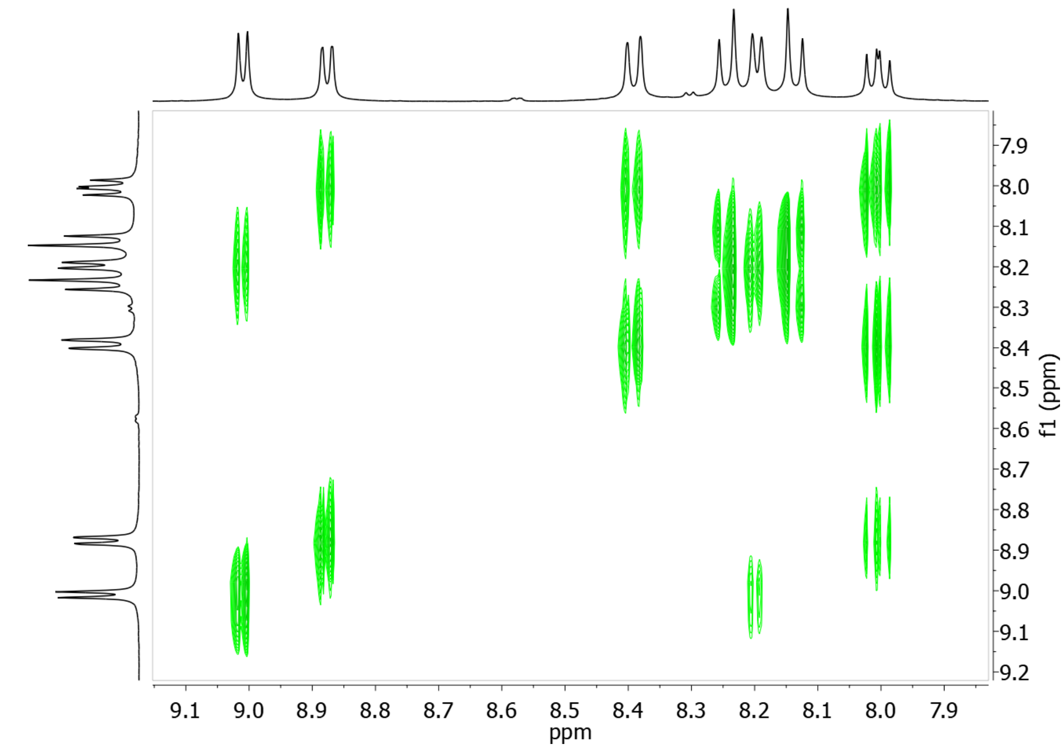

Figure S19. ^{1}H-1H COSY45 NMR spectrum of 4MPO in D_2O (400 MHz, 25 °C). Inset: Selected region of the aromatic part.

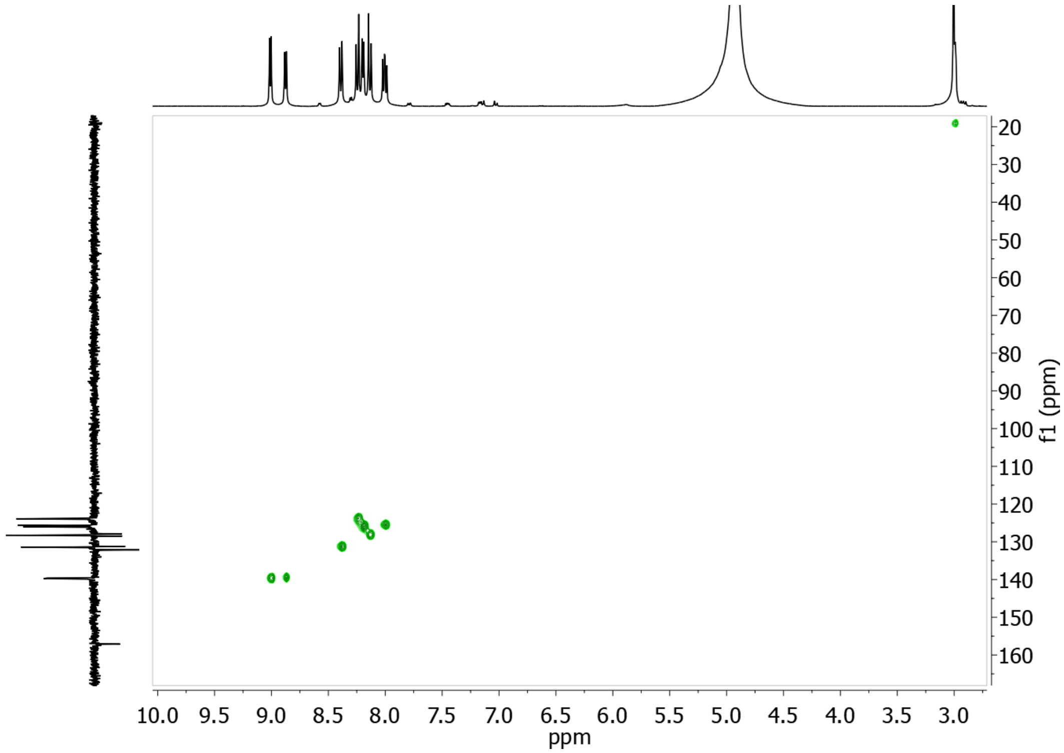

Figure S20. (^{1}H–^{13}C) HSQC NMR spectrum of 4MPO in D_2O (400 MHz, 25 °C).

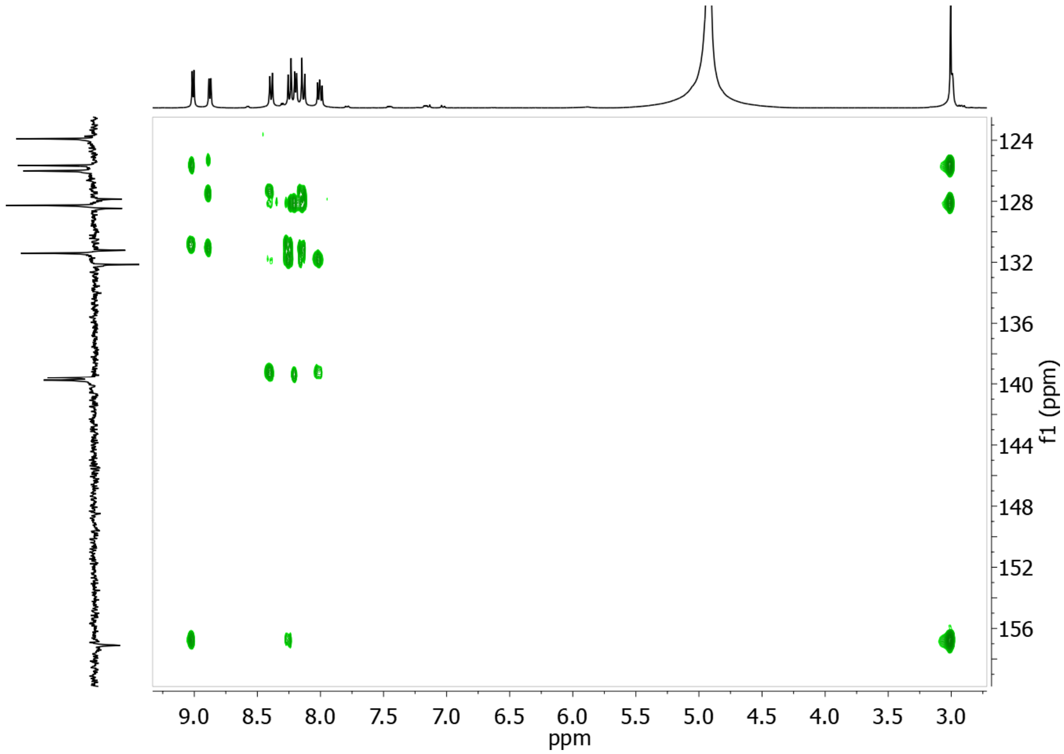

Figure S21. (^1H–^{13}C) HMBC NMR spectrum of 4MPO in D_2O (400 MHz, 25 °C).

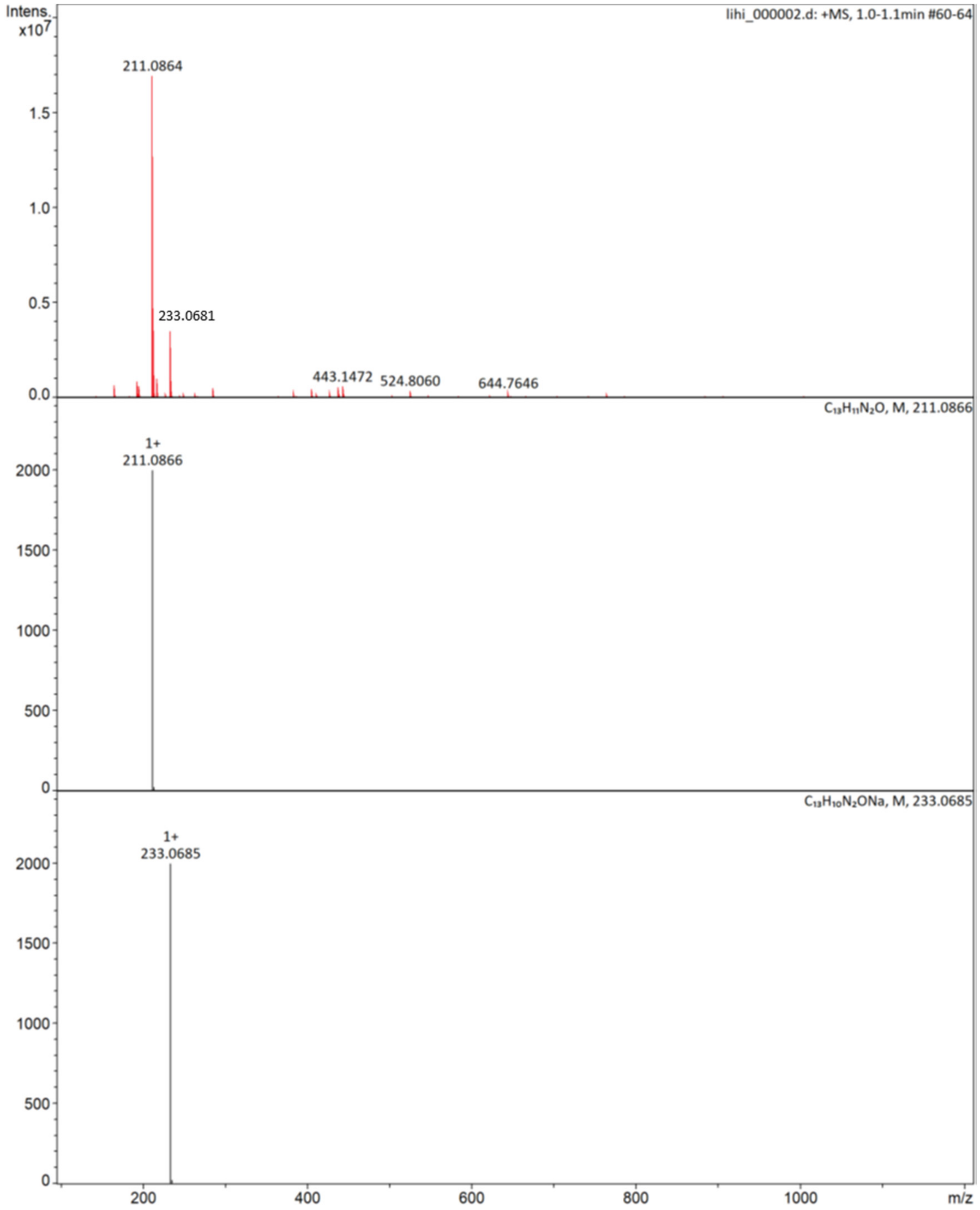

Figure S22. HRMS spectrum of 4MPO in positive mode.

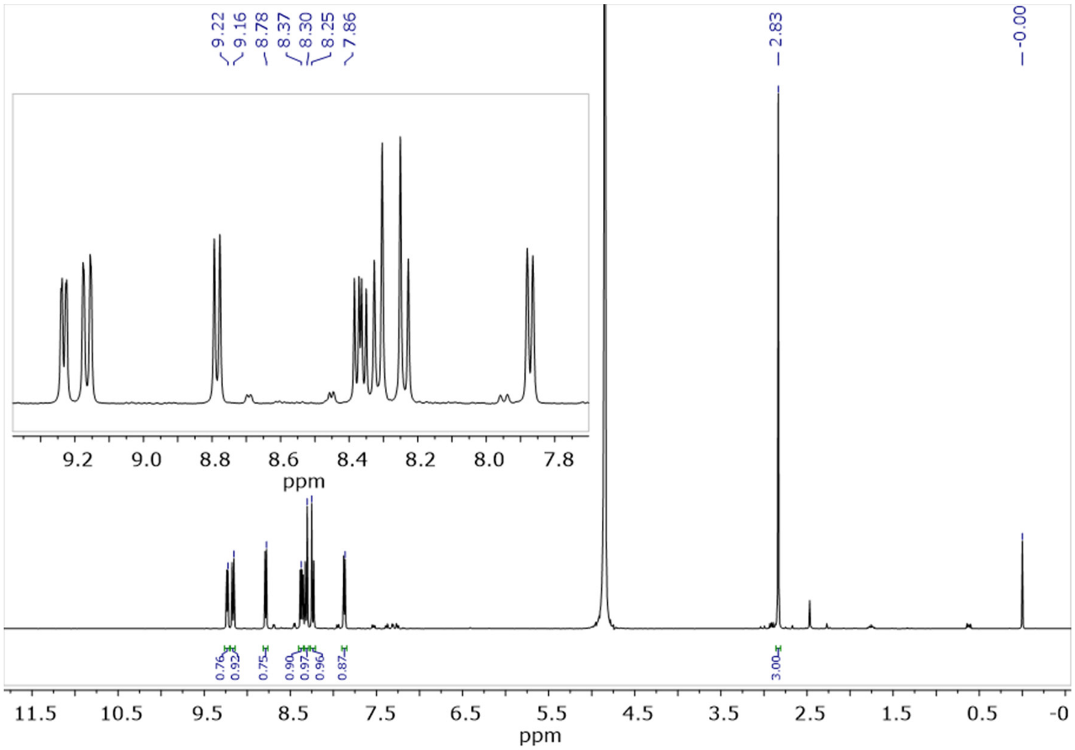

Figure S23. ^{1}H NMR spectrum of 7MPO in D_2O (400 MHz, 25 °C). Inset: Selected region of the aromatic part.

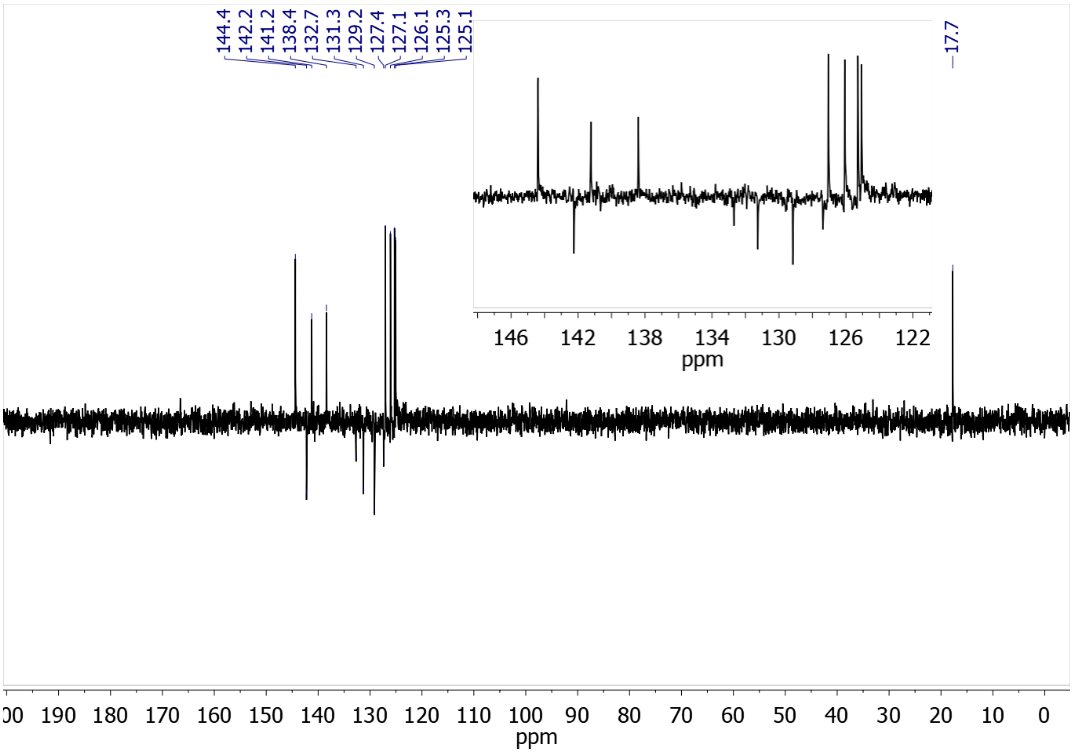

Figure S24. APT ^{13}C NMR spectrum of 7MPO in D_2O (100.6 MHz, 25 °C). Inset: Selected region of the aromatic part.

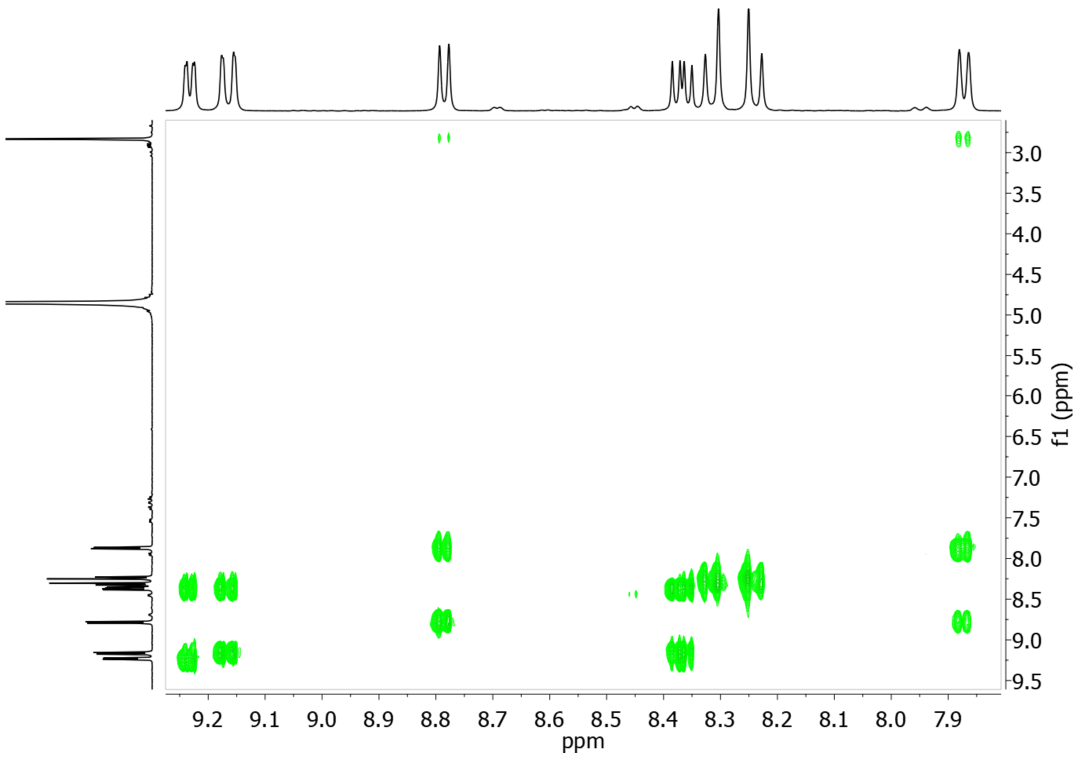

Figure S25. ^1H-^1H COSY45 NMR spectrum of 7MPO in D_2O (400 MHz, 25 °C). Inset: Selected region of the aromatic part.

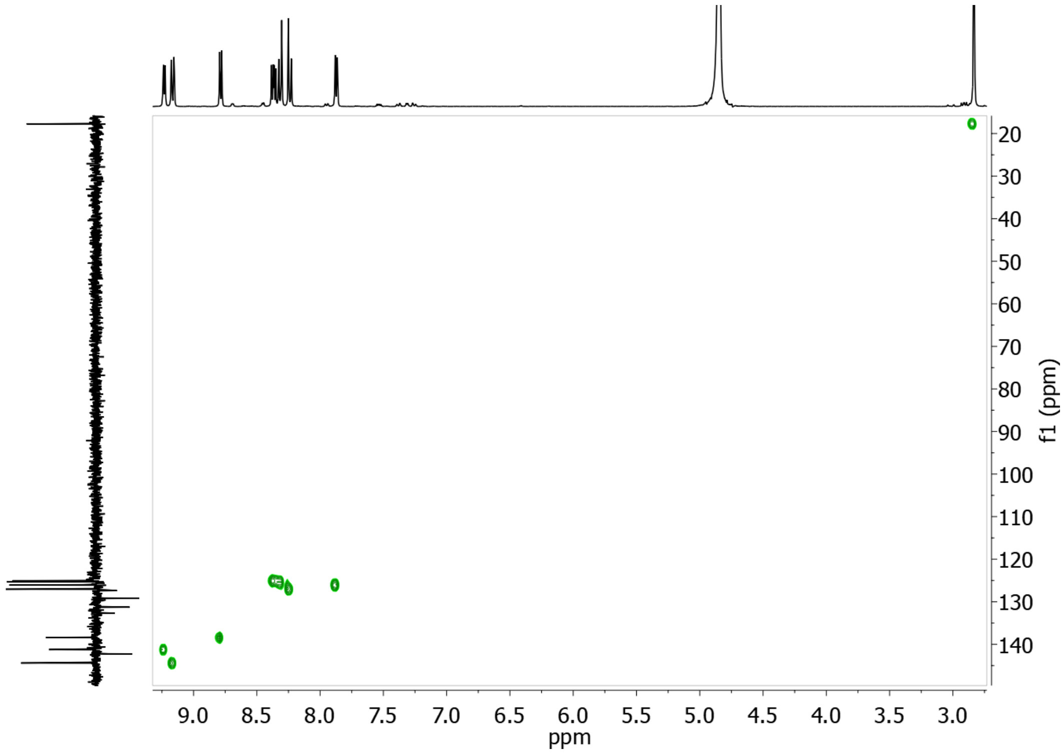

Figure S26. (^1H–^{13}C) HSQC NMR spectrum of 7MPO in D_2O (400 MHz, 25 °C).

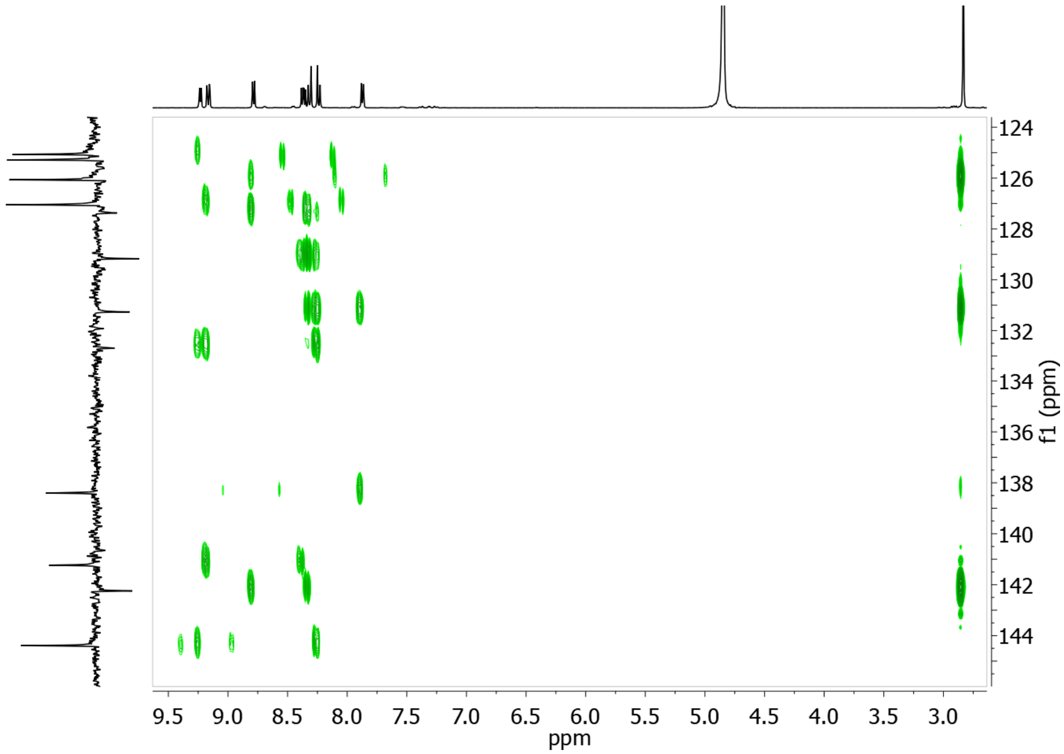

Figure S27. (^1H–^{13}C) HMBC NMR spectrum of 7MPO in D_2O (400 MHz, 25 °C).

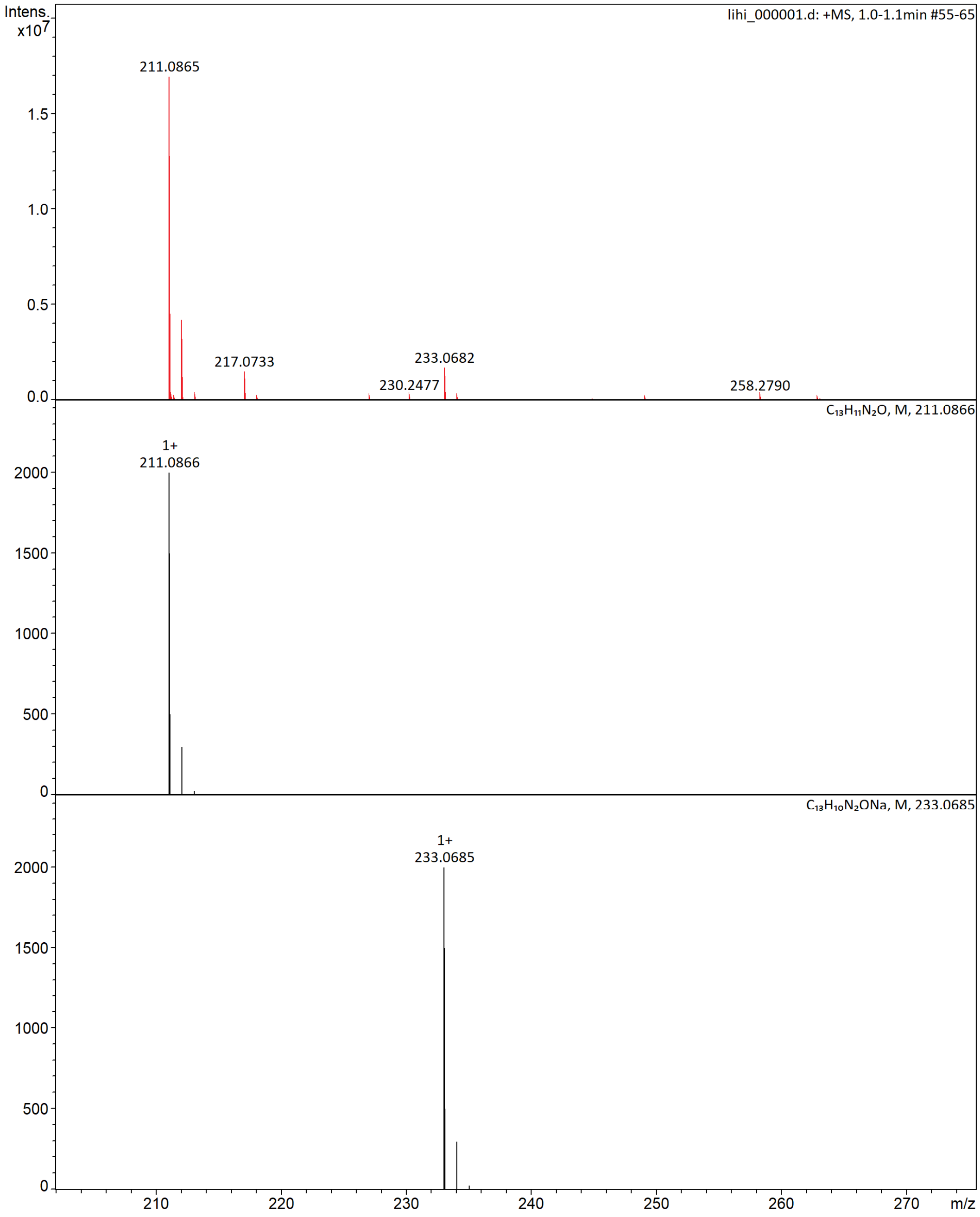

Figure S28. HRMS spectrum of 7MPO in positive mode.

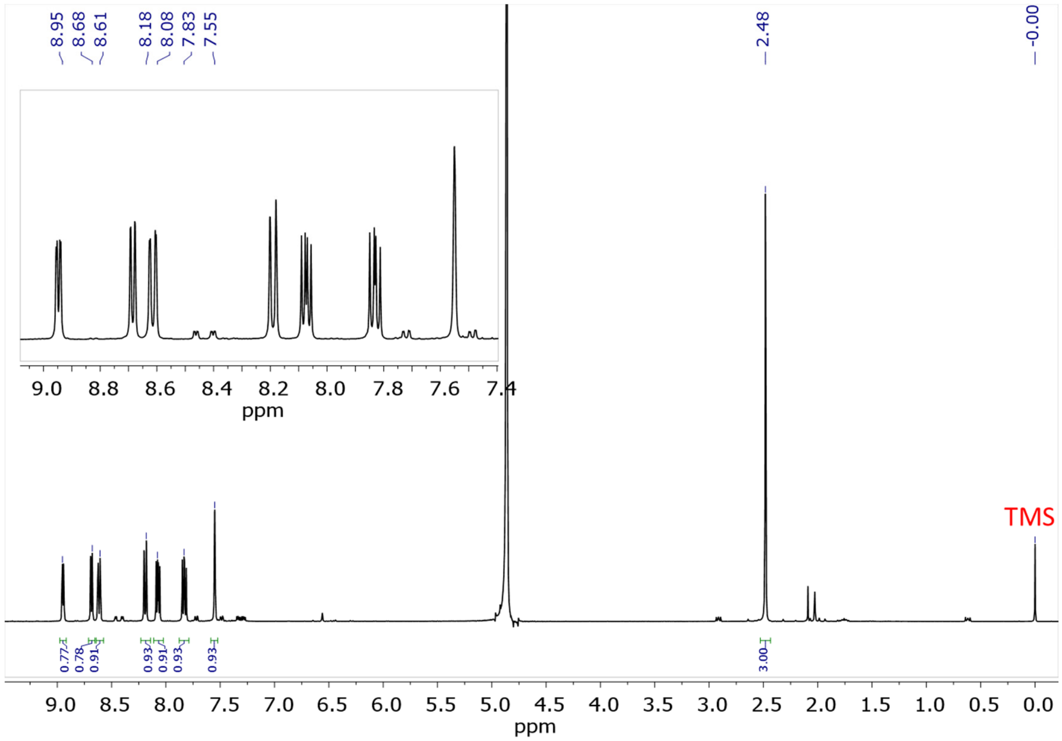

Figure S29. ^{1}H NMR spectrum of 5MPO in D_2O (400 MHz, 25 °C). Inset: Selected region of the aromatic part.

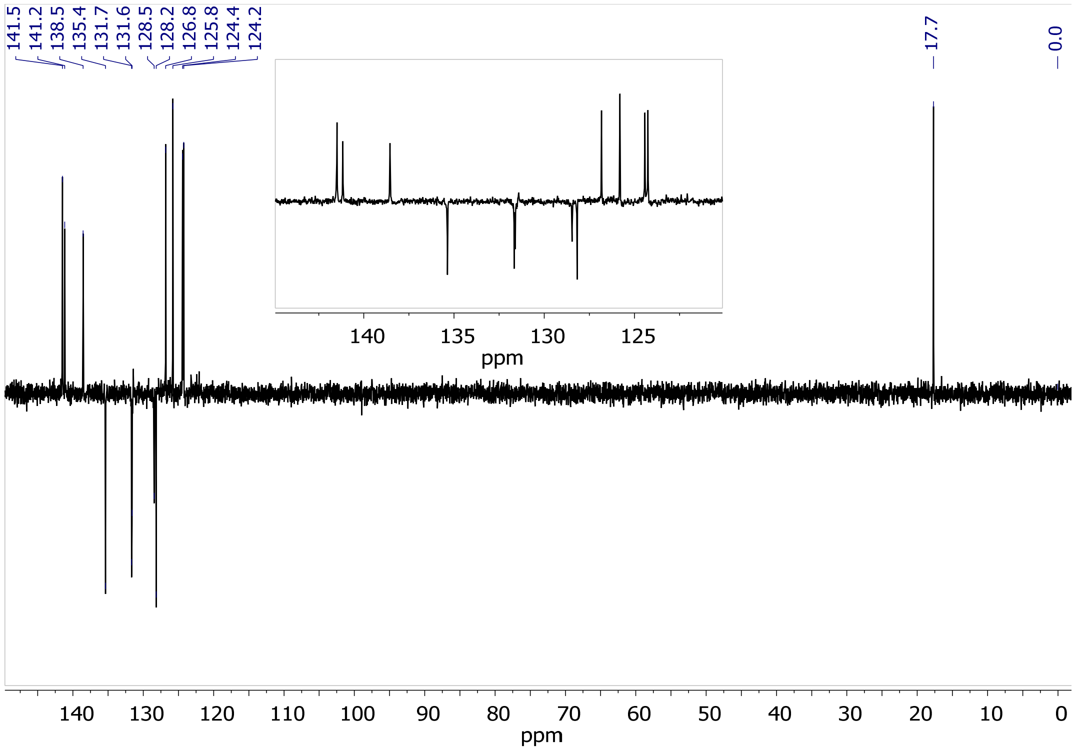

Figure S30. APT ^{13}C NMR spectrum of 5MPO in D_2O (100.6 MHz, 25 °C). Inset: Selected region of the aromatic part.

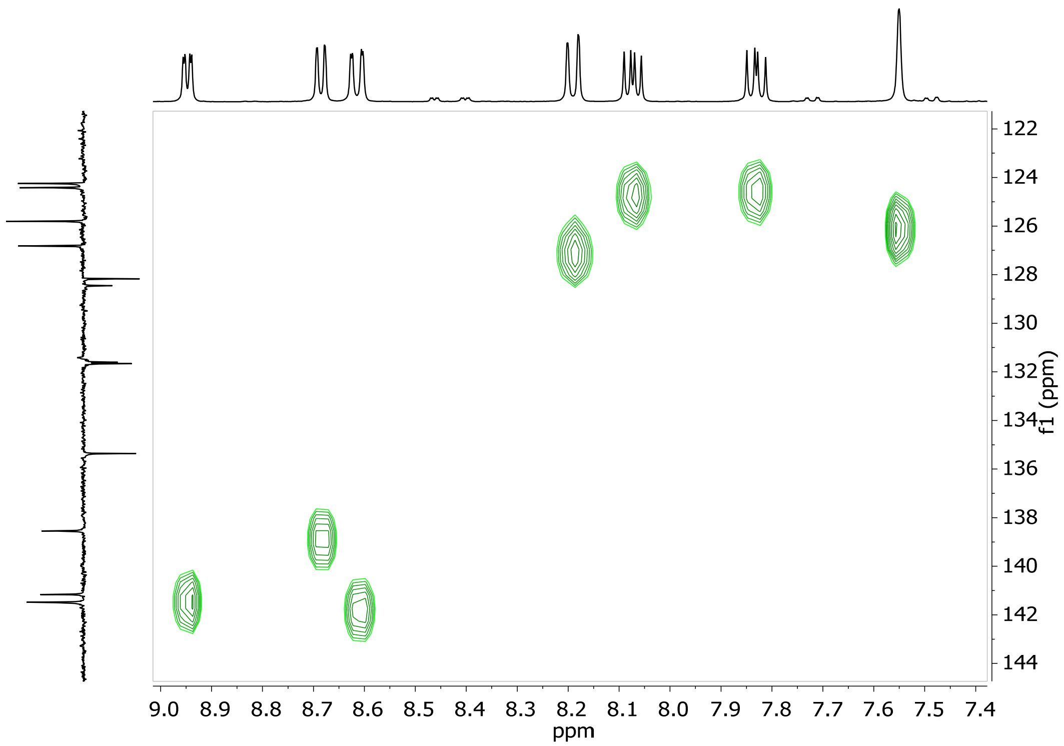

Figure S31. (^{1}H–^{13}C) HSQC NMR spectrum of 5MPO in D_2O (400 MHz, 25 °C).

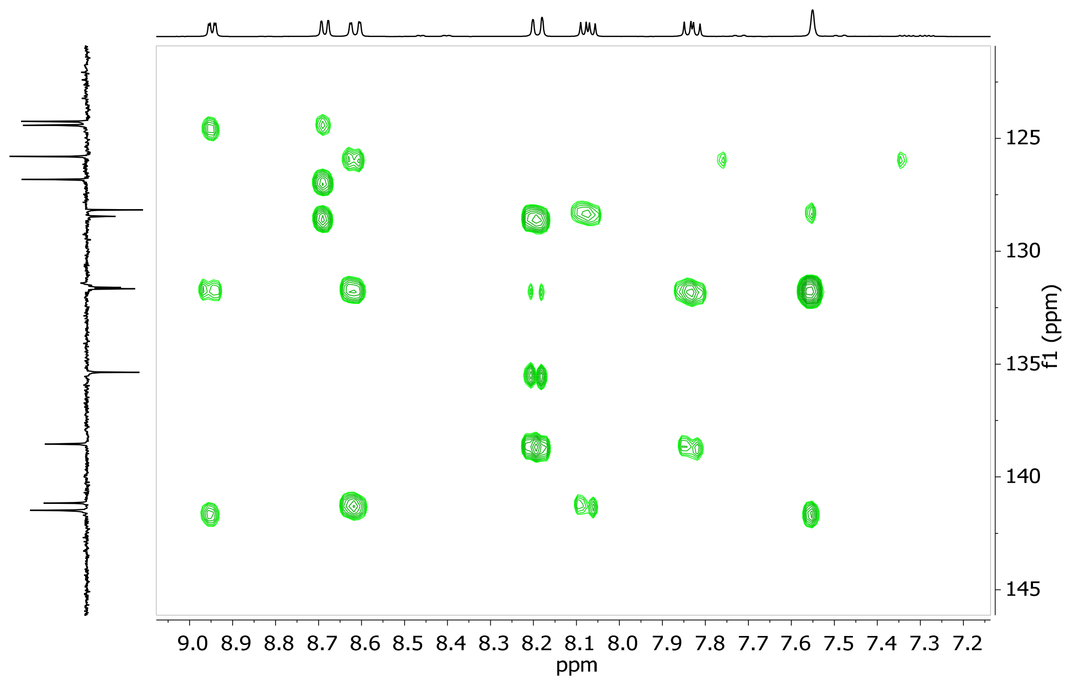

Figure S32. (^{1}H–^{13}C) HMBC NMR spectrum of 5MPO in D_2O (400 MHz, 25 °C).

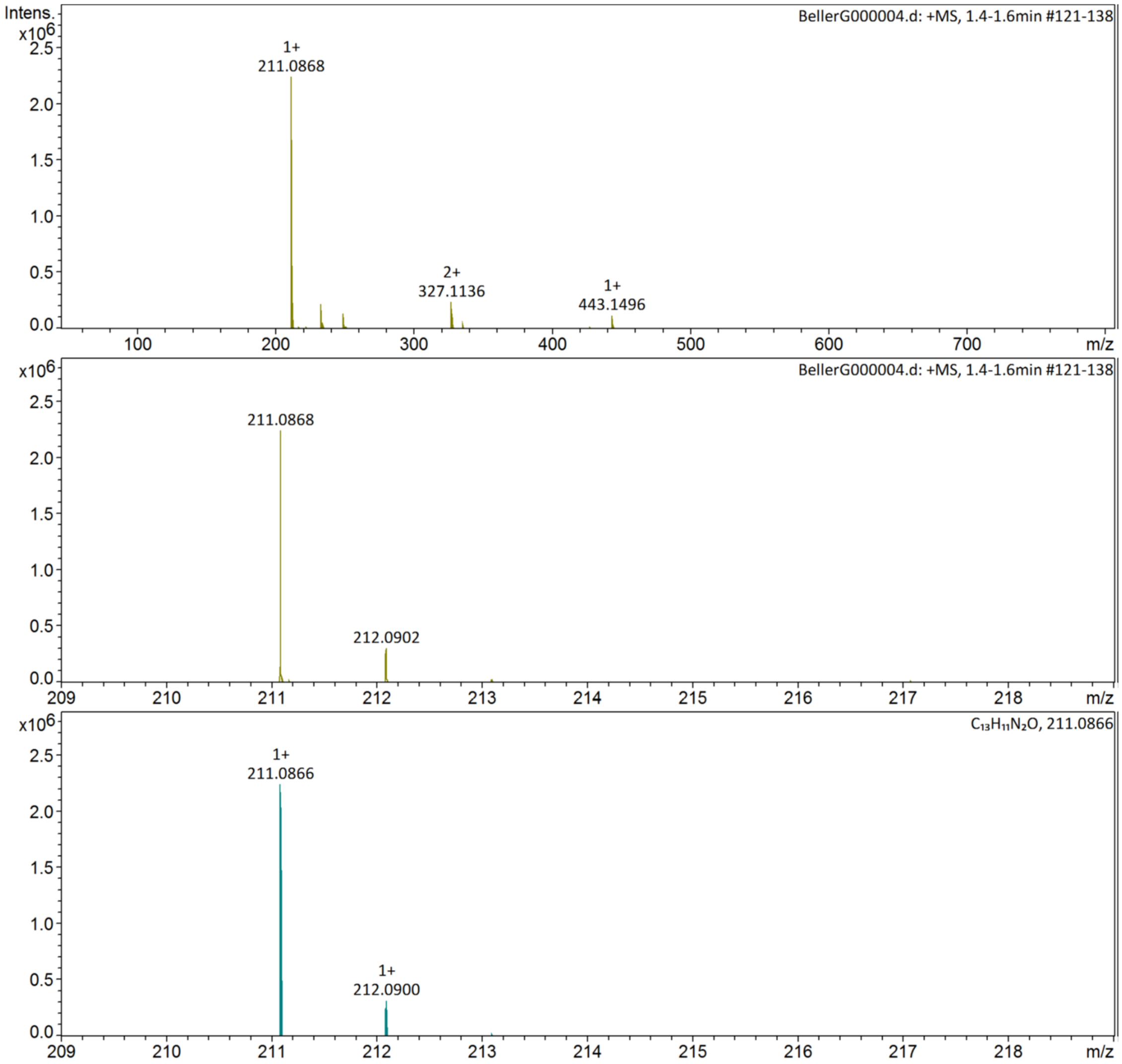

Figure S33. HRMS spectrum of 5MPO in positive mode.

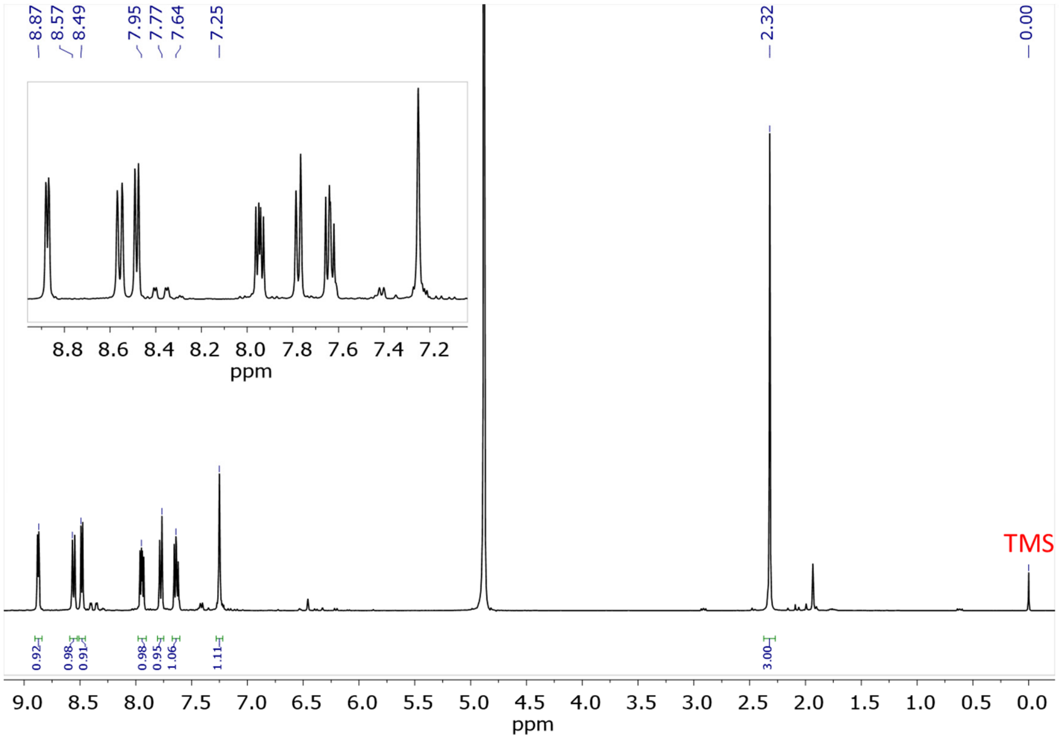

Figure S34. ^{1}H NMR spectrum of 6MPO in D_2O (400 MHz, 25 °C). Inset: Selected region of the aromatic part.

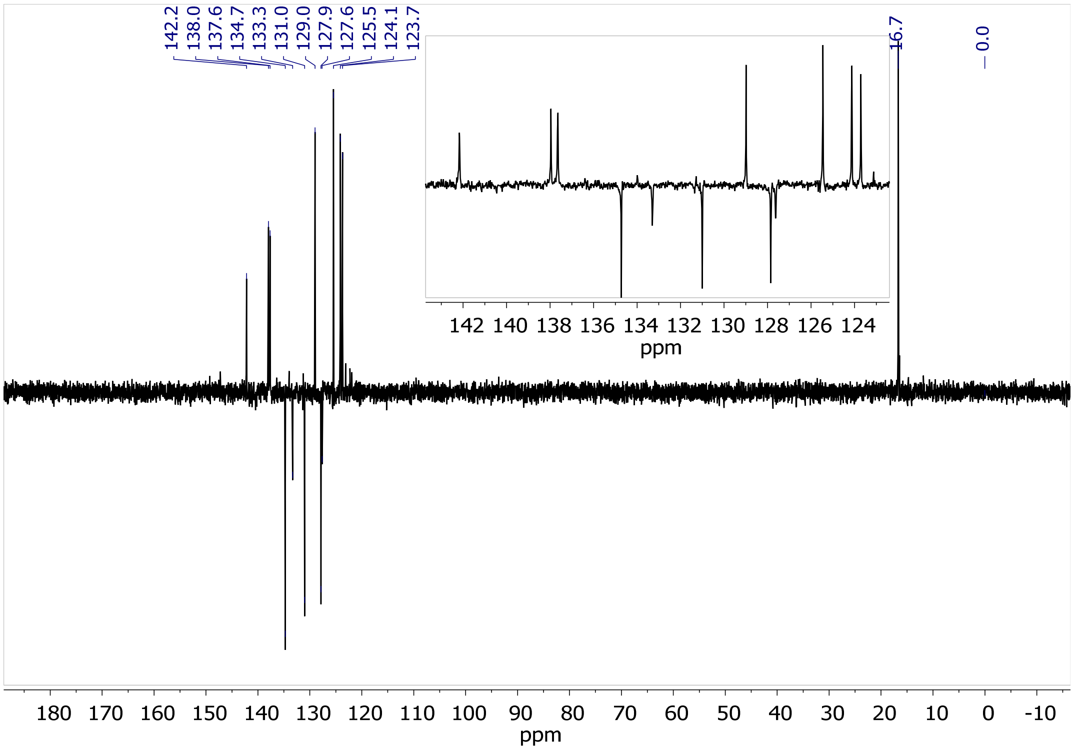

Figure S35. APT ^{13}C NMR spectrum of 6MPO in D_2O (100.6 MHz, 25 °C). Inset: Selected region of the aromatic part.

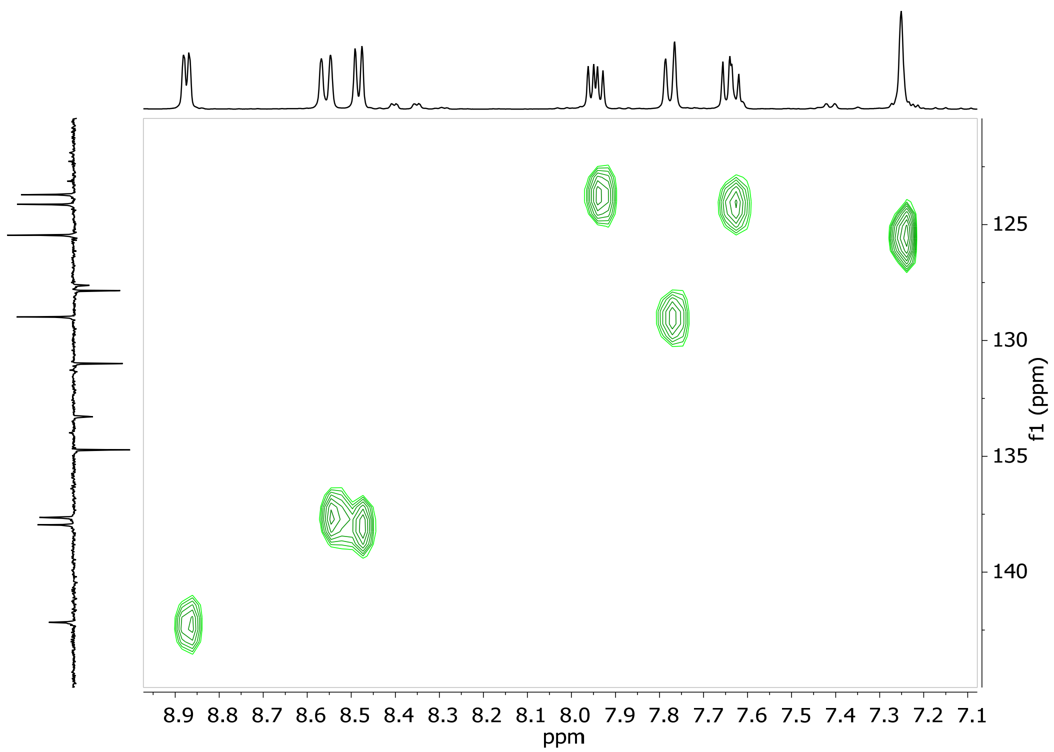

Figure S36. (^{1}H–^{13}C) HSQC NMR spectrum of 6MPO in D_2O (400 MHz, 25 °C).

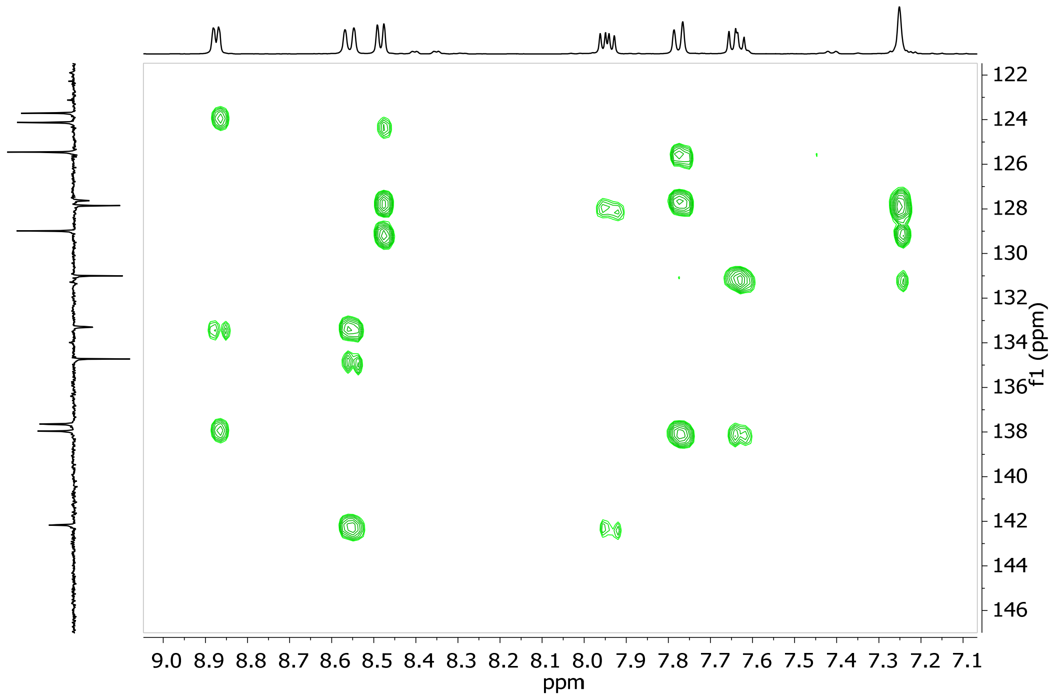

Figure S37. (^{1}H–^{13}C) HMBC NMR spectrum of 6MPO in D_2O (400 MHz, 25 °C).

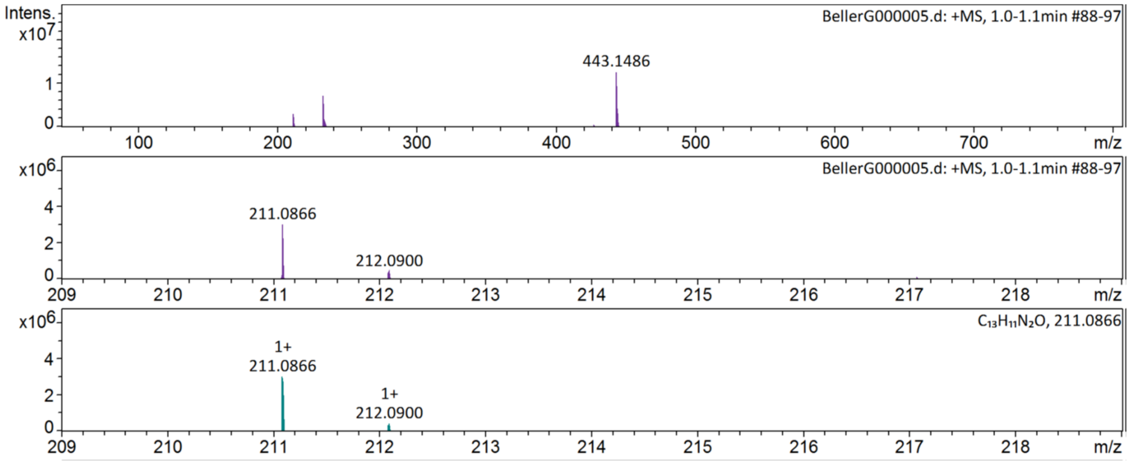

Figure S38. HRMS spectrum of 6MPO in positive mode.

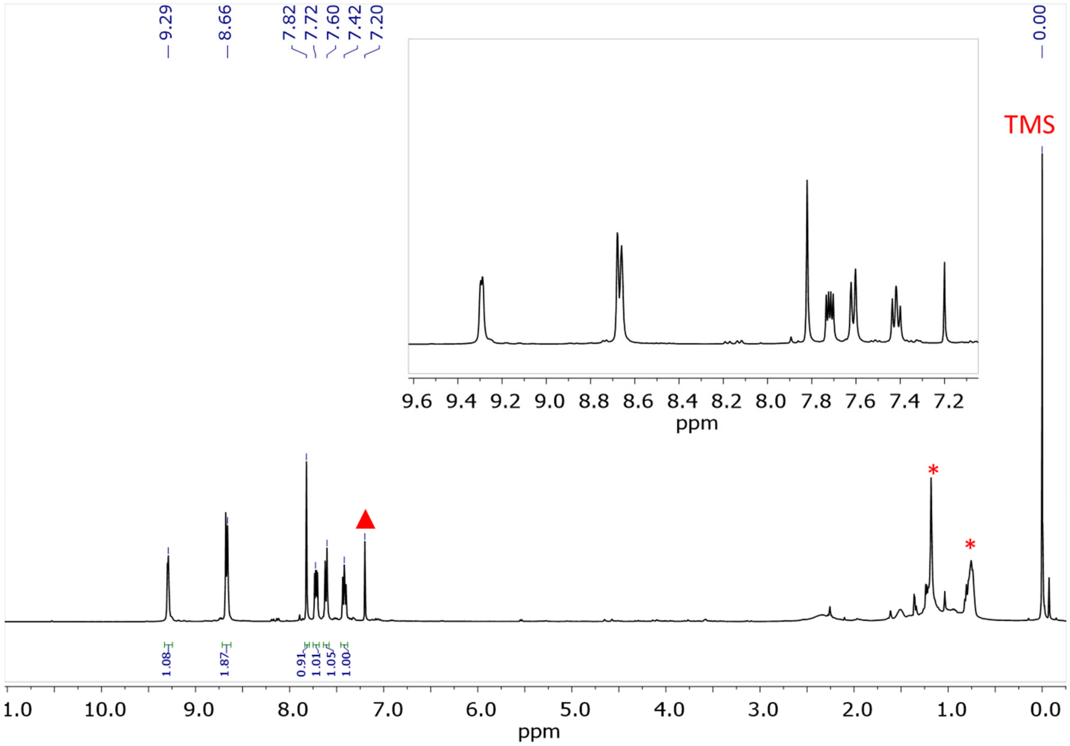

Figure S39. ^{1}H NMR spectrum of 5CPO in $CDCl_3$ (400 MHz, 25 °C). ▲ indicates solvent residual peak and asterisks show solvent impurity. Inset: Selected region of the aromatic part.

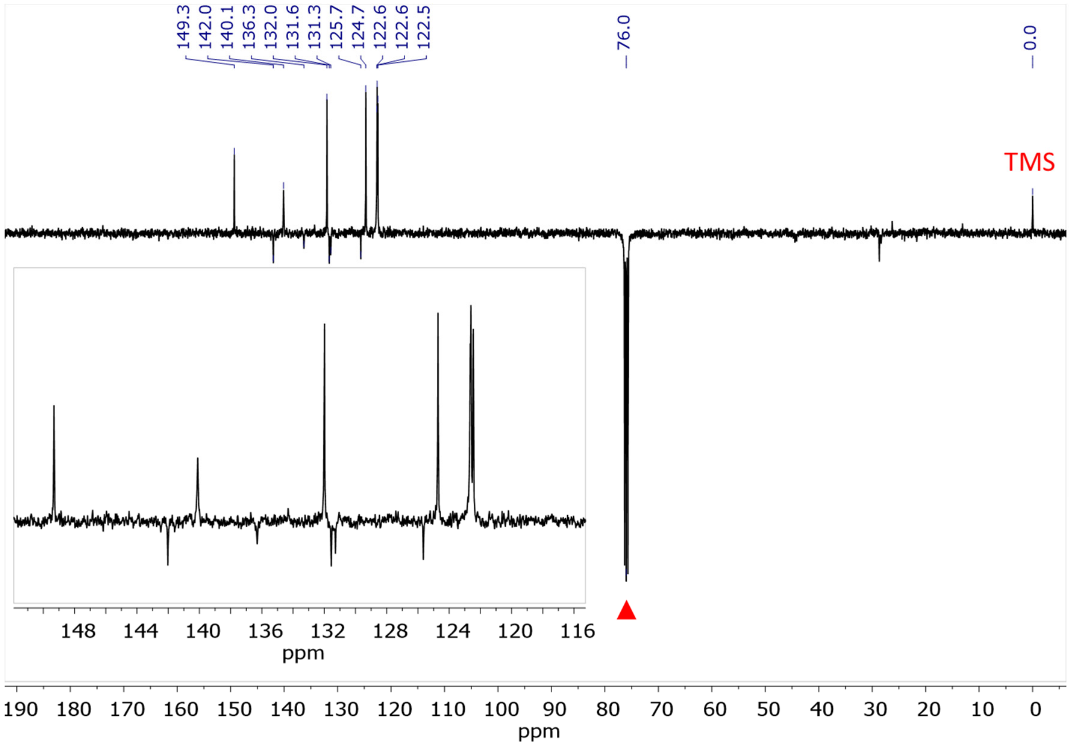

Figure S40. APT ^{13}C NMR spectrum of 5CPO in $CDCl_3$ (100.6 MHz, 25 °C). ▲ indicates the solvent residual peak. Inset: Selected region of the aromatic part.

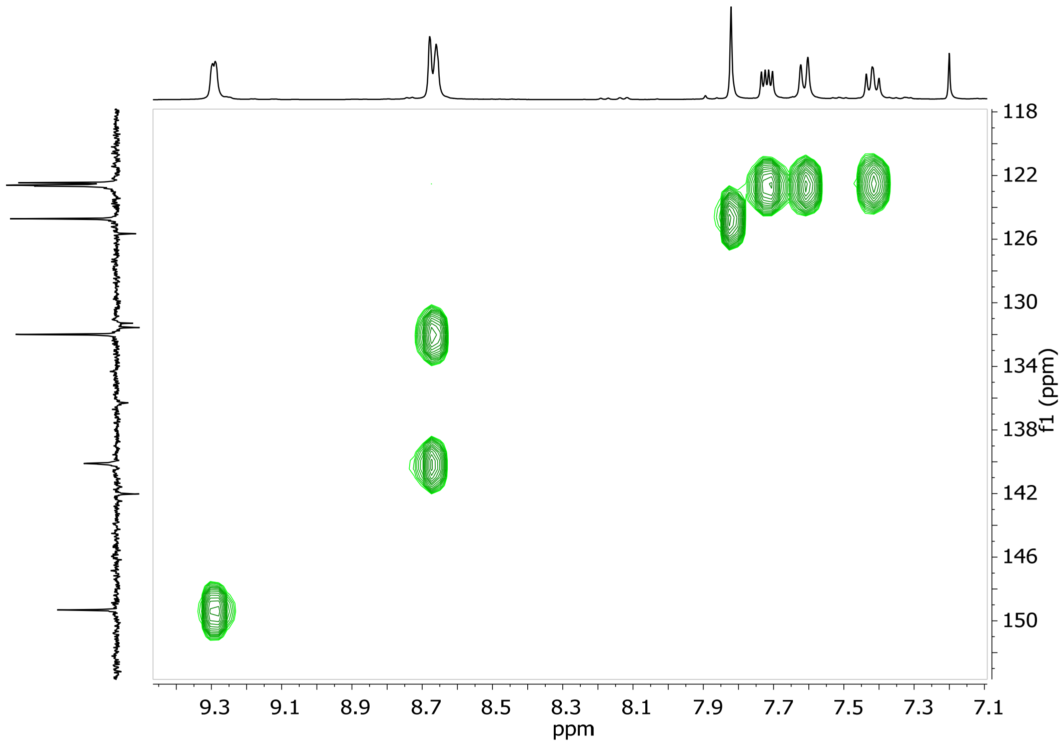

Figure S41. (^1H–^{13}C) HSQC NMR spectrum of 5CPO in $CDCl_3$ (400 MHz, 25 °C).

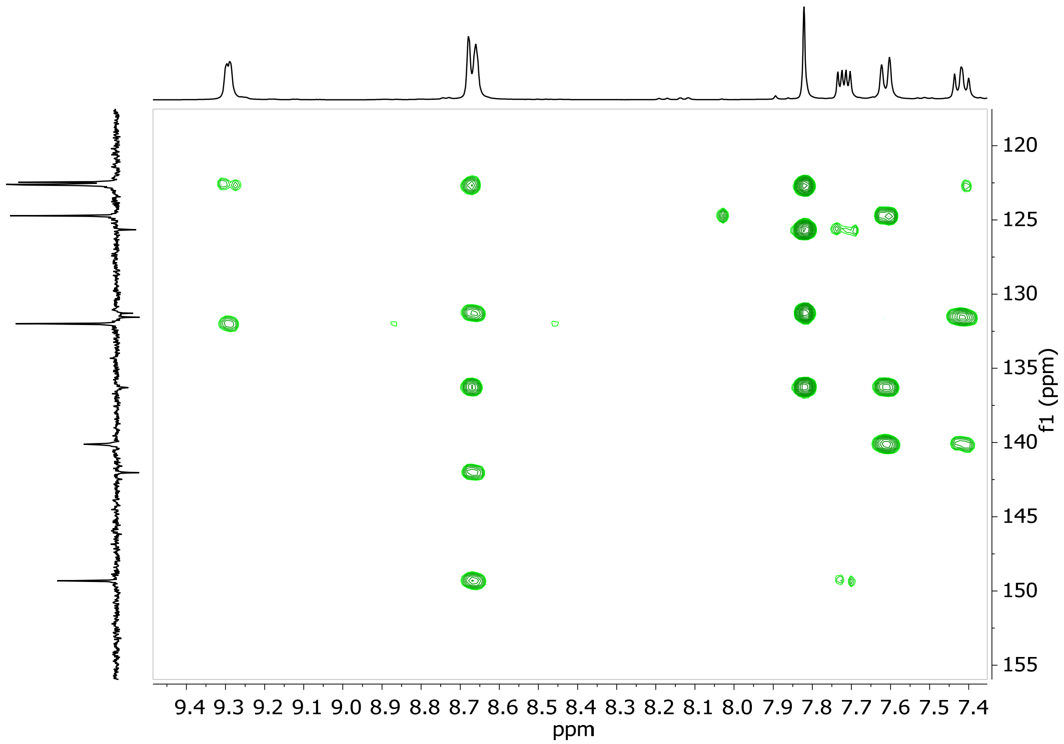

Figure S42. (^{1}H–^{13}C) HMBC NMR spectrum of 5CPO in $CDCl_3$ (400 MHz, 25 °C).

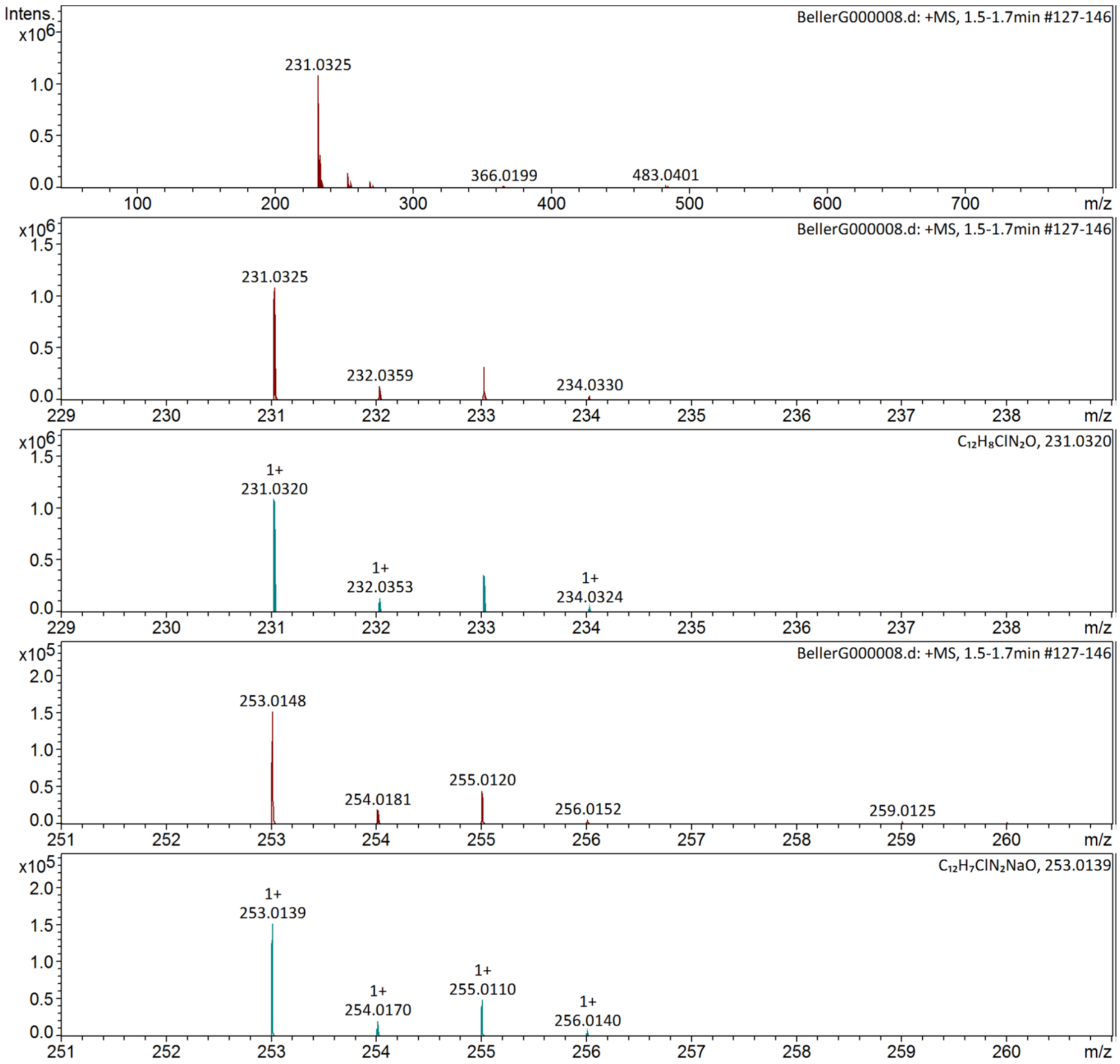

Figure S43. HRMS spectrum of 5CPO in positive mode.

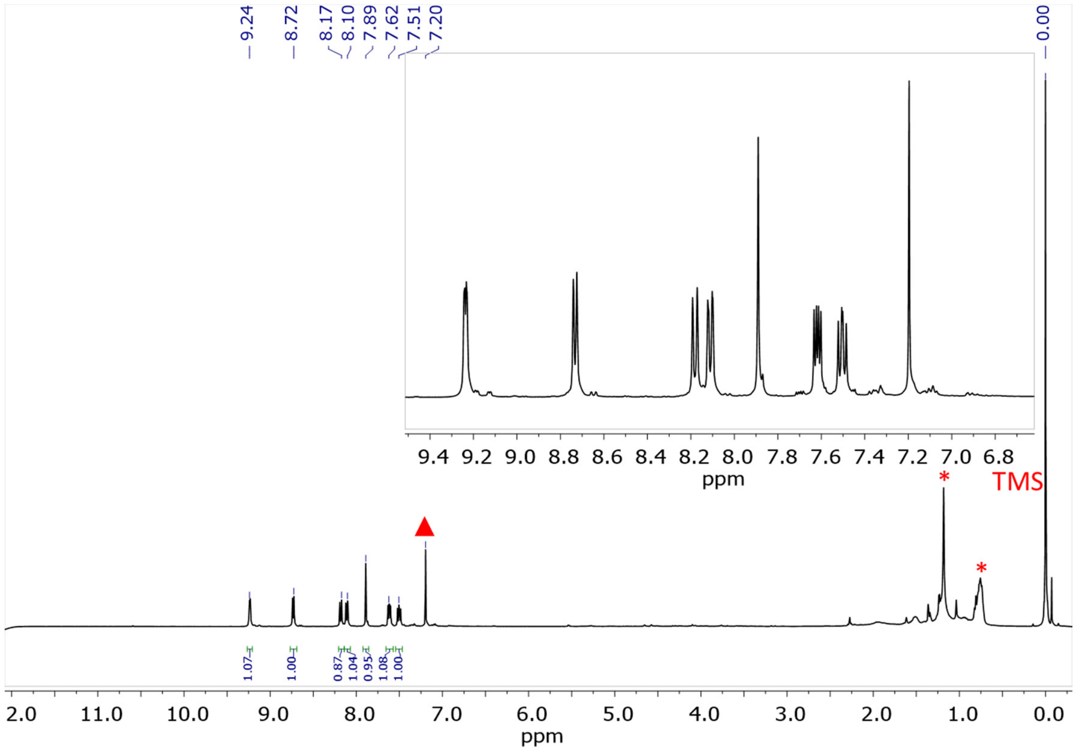

Figure S44. ^{1}H NMR spectrum of 6CPO in $CDCl_3$ (400 MHz, 25 °C). ▲ indicates solvent residual peak and asterisks show solvent impurity. Inset: Selected region of the aromatic part.

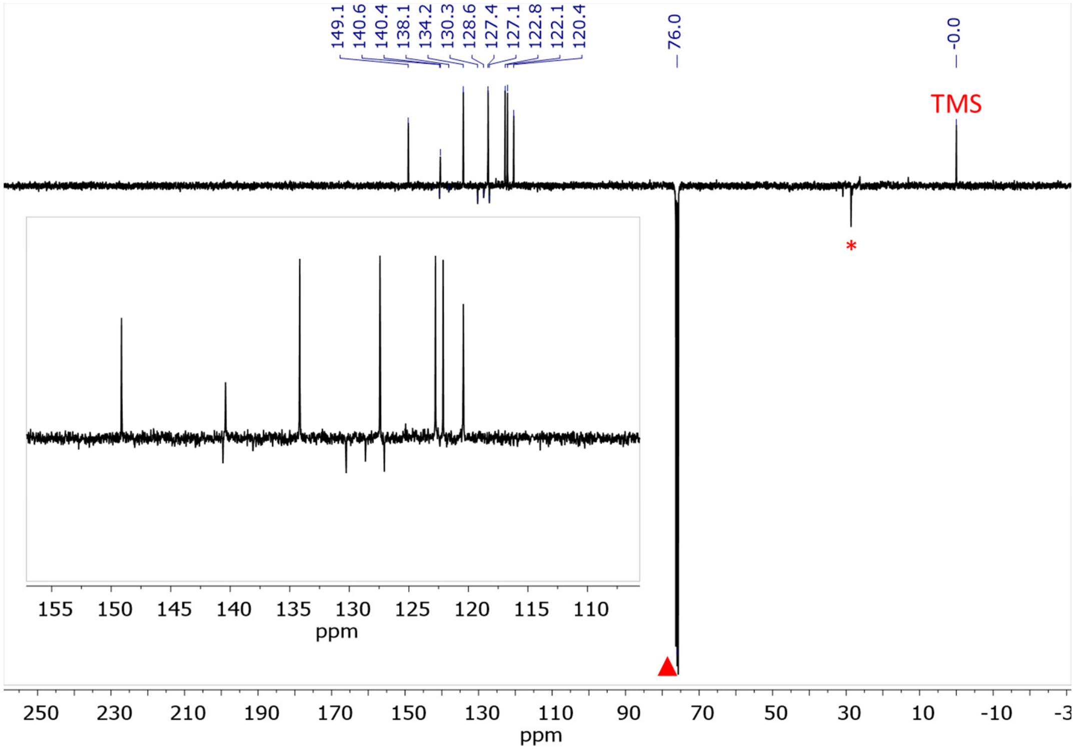

Figure S45. APT ^{13}C NMR spectrum of 6CPO in $CDCl_3$ (100.6 MHz, 25 °C). ▲ indicates the solvent residual peak and asterisk shows solvent impurity. Inset: Selected region of the aromatic part.

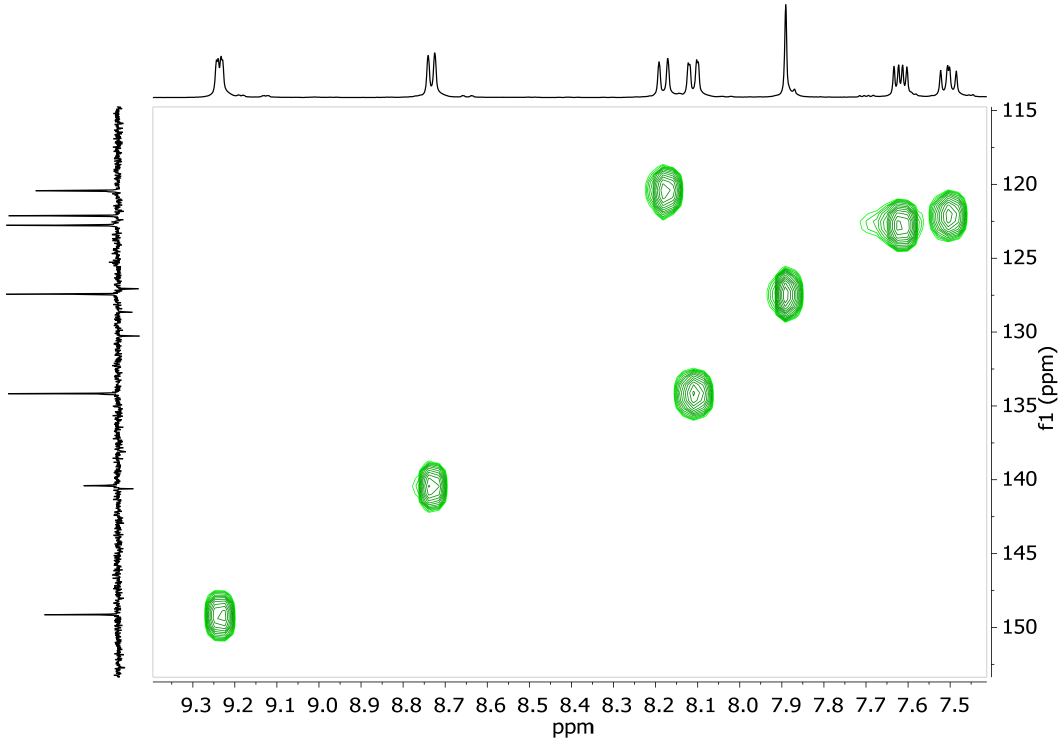

Figure S46. (^{1}H–^{13}C) HSQC NMR spectrum of 6CPO in $CDCl_3$ (400 MHz, 25 °C).

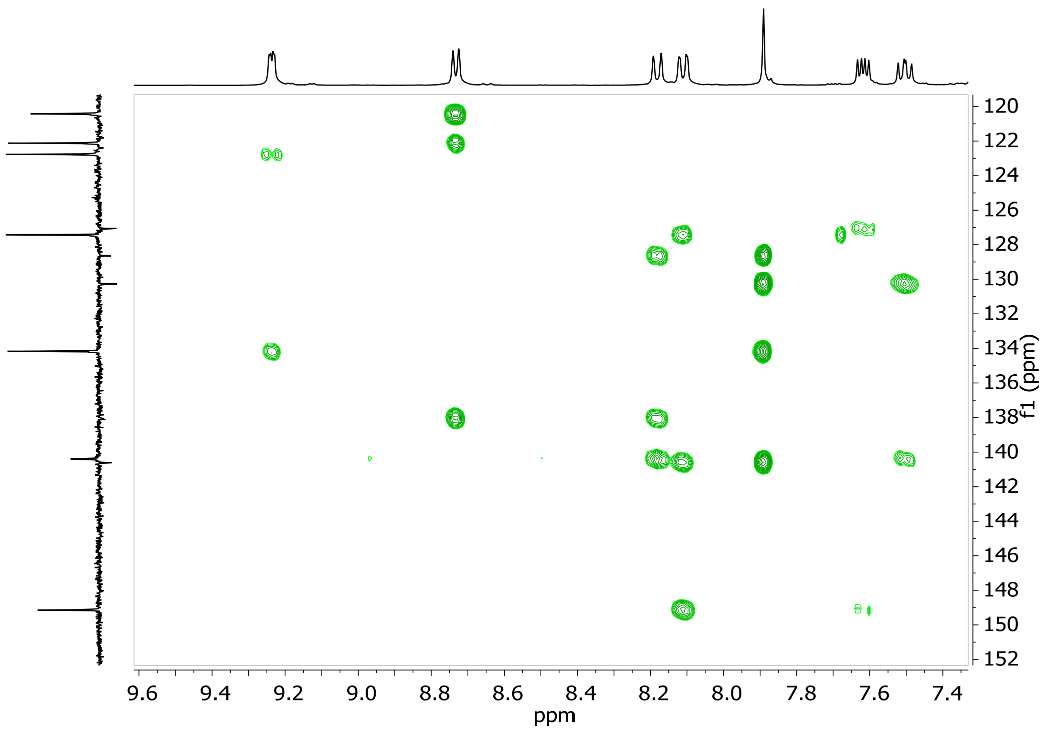

Figure S47. (^{1}H–^{13}C) HMBC NMR spectrum of 6CPO in $CDCl_3$ (400 MHz, 25 °C).

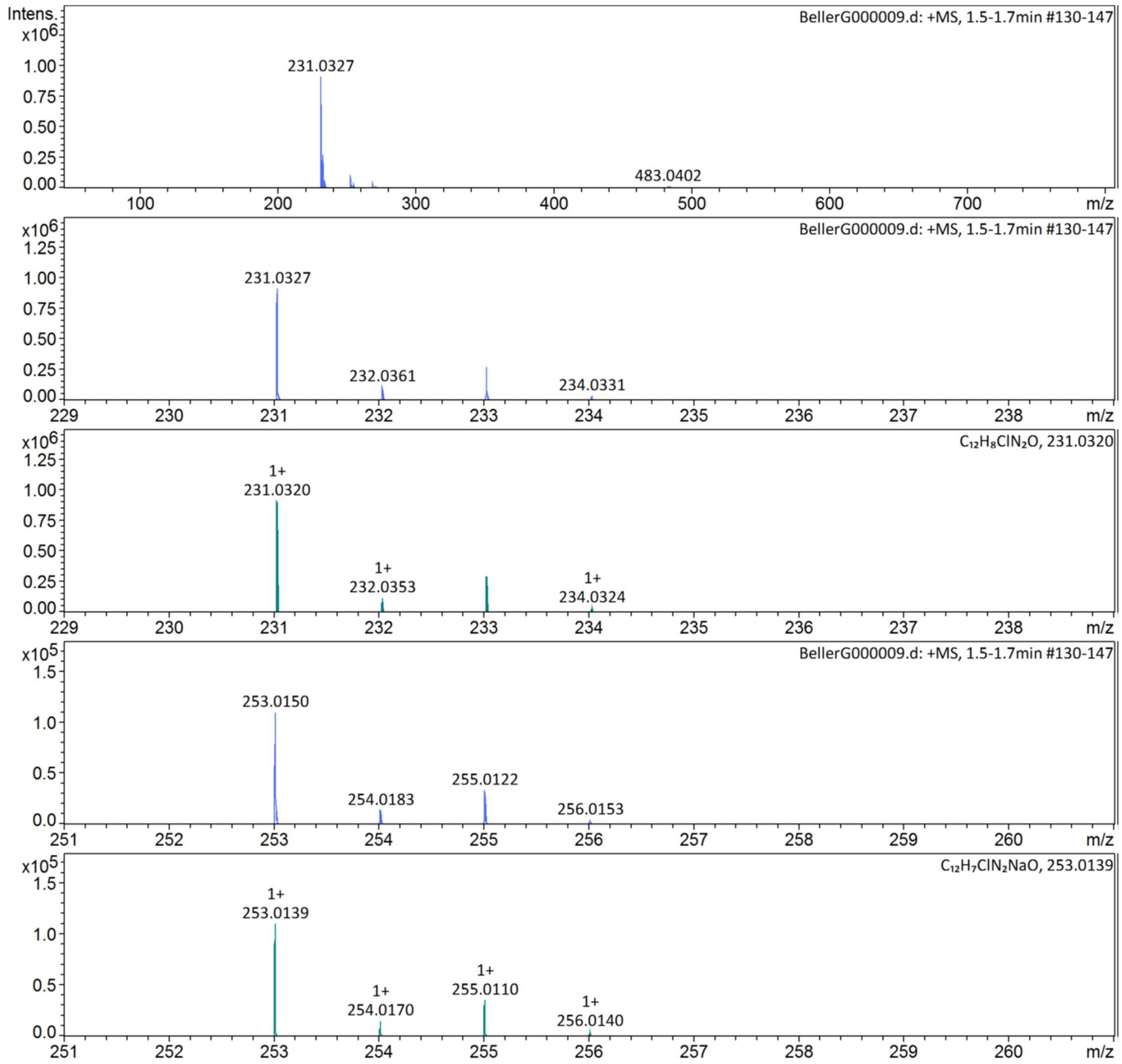

Figure S48. HRMS spectrum of 6CPO in positive mode.

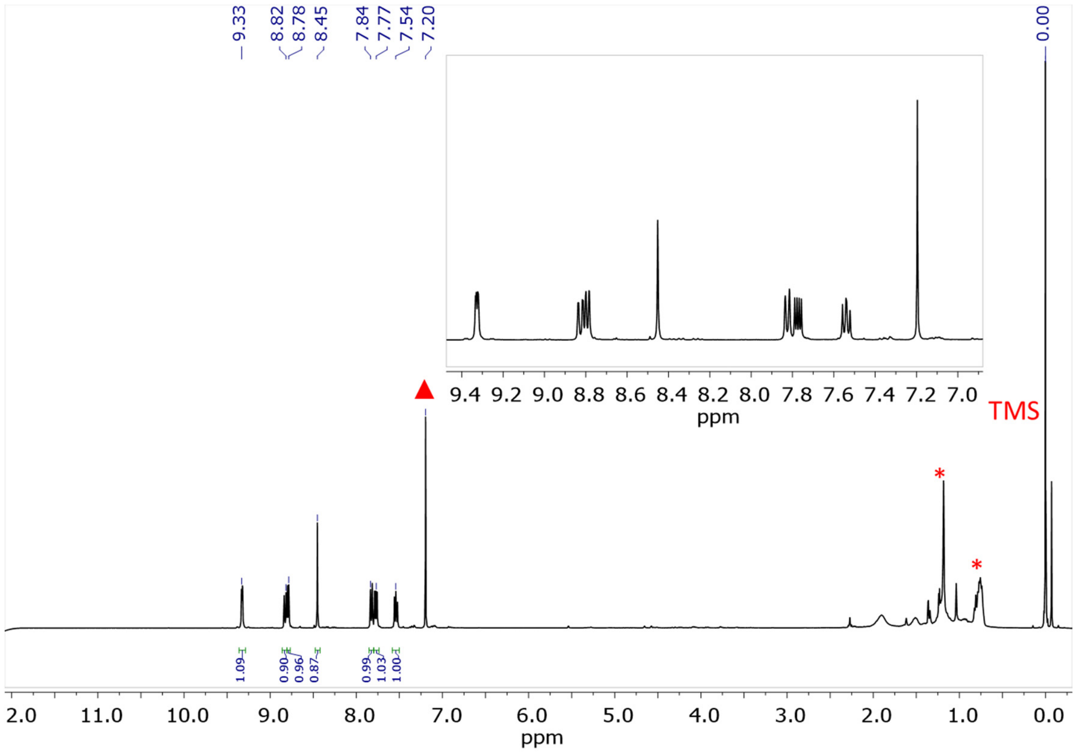

Figure S49. ^{1}H NMR spectrum of 5NPO in $CDCl_3$ (400 MHz, 25 °C). ▲ indicates solvent residual peak and asterisks show solvent impurity. Inset: Selected region of the aromatic part.

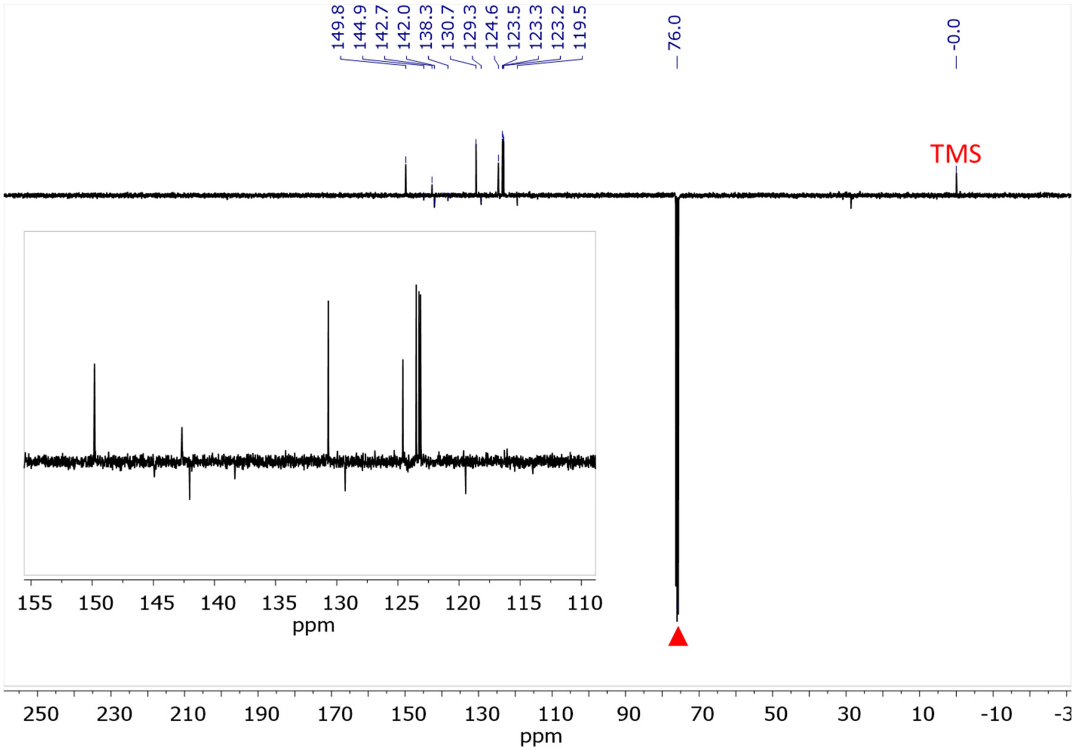

Figure S50. APT ^{13}C NMR spectrum of 5NPO in $CDCl_3$ (100.6 MHz, 25 °C). ▲ indicates the solvent residual peak. Inset: Selected region of the aromatic part.

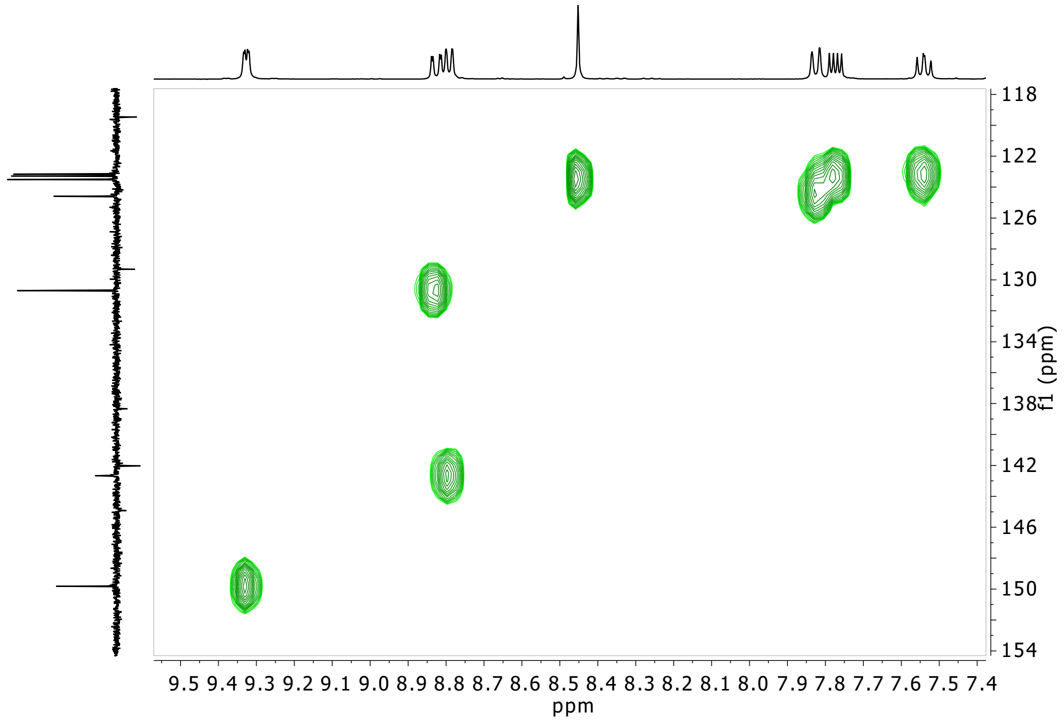

Figure S51. (^{1}H–^{13}C) HSQC NMR spectrum of 5NPO in $CDCl_3$ (400 MHz, 25 °C).

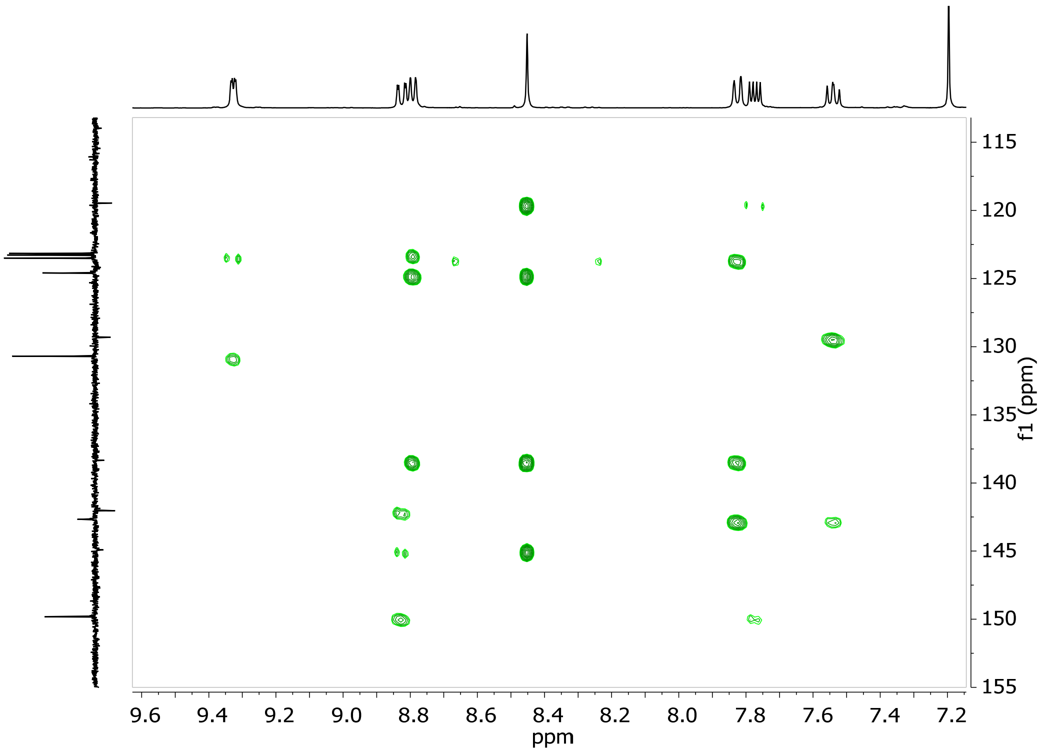

Figure S52. (^{1}H–^{13}C) HMBC NMR spectrum of 5NPO in $CDCl_3$ (400 MHz, 25 °C).

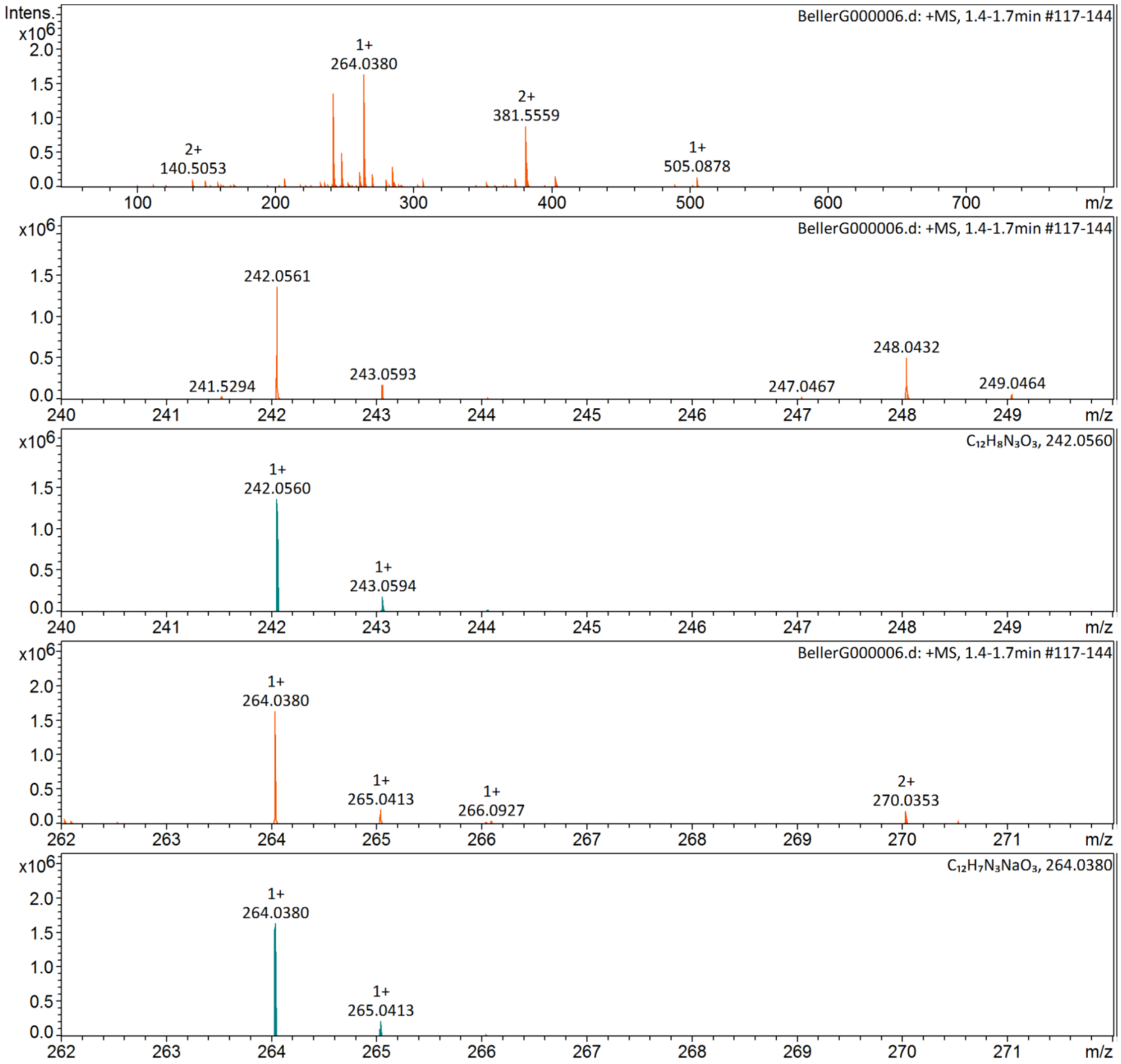

Figure S53. HRMS spectrum of 5NPO in positive mode.

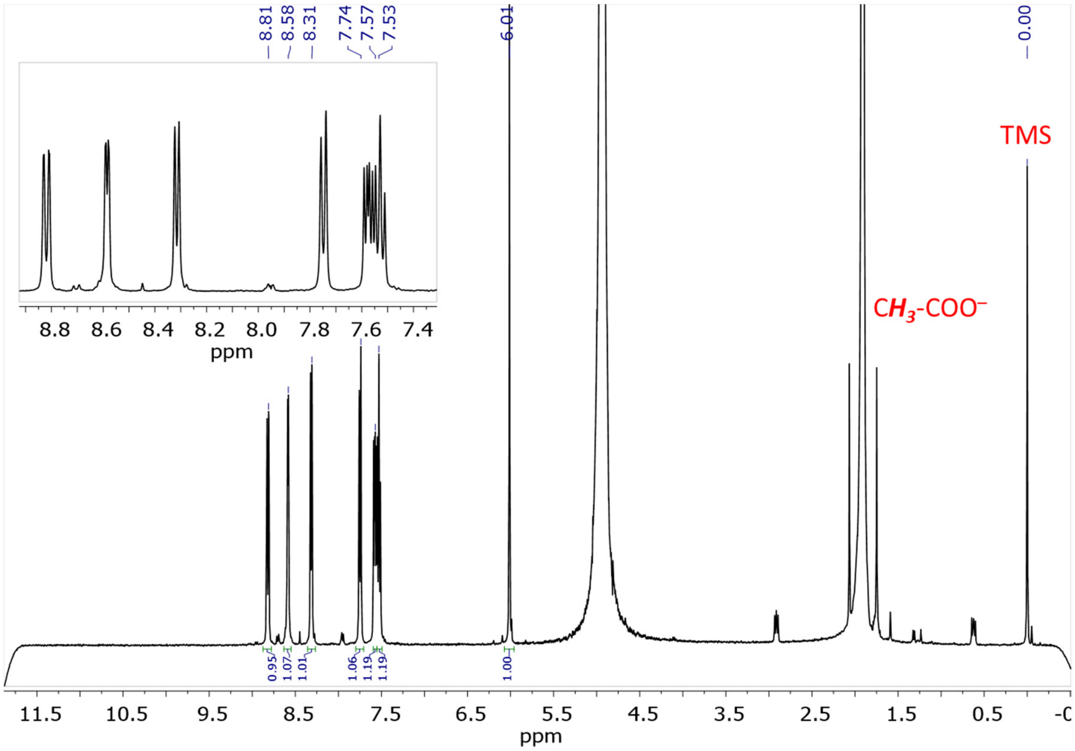

Figure S54. ^{1}H NMR spectrum of 6NPO in D_2O (400 MHz, 25 °C). Inset: Selected region of the aromatic part.

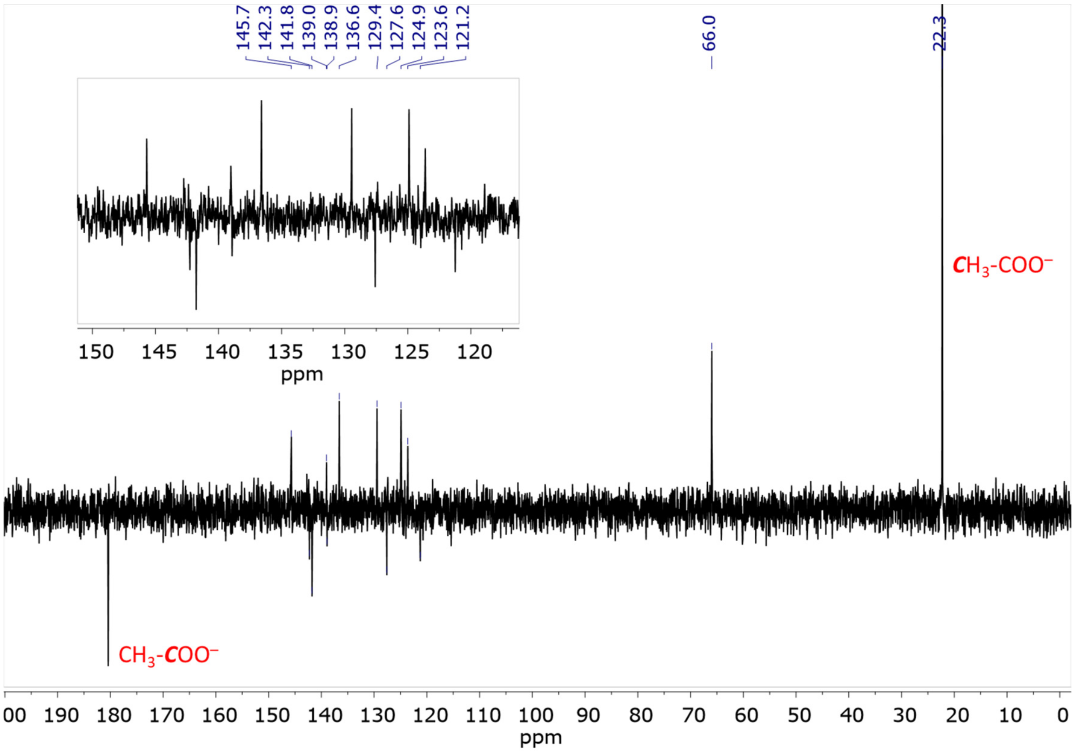

Figure S55. APT ^{13}C NMR spectrum of 6NPO in D_2O (100.6 MHz, 25 °C). Inset: Selected region of the aromatic part.

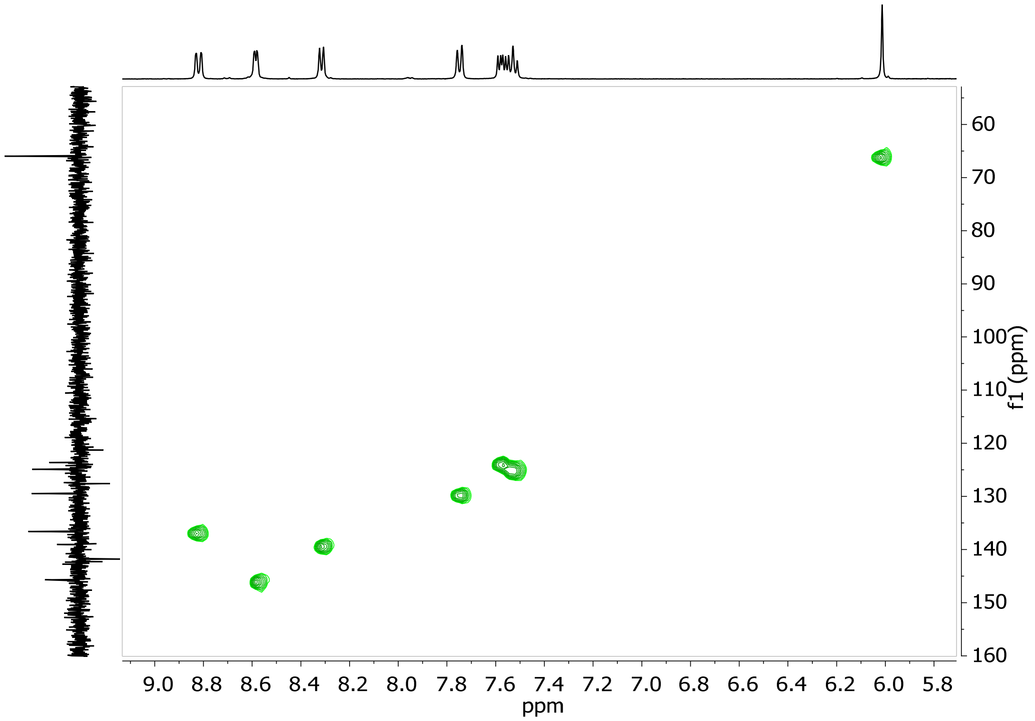

Figure S56. (^1H–^{13}C) HSQC NMR spectrum of 6NPO in D_2O (400 MHz, 25 °C).

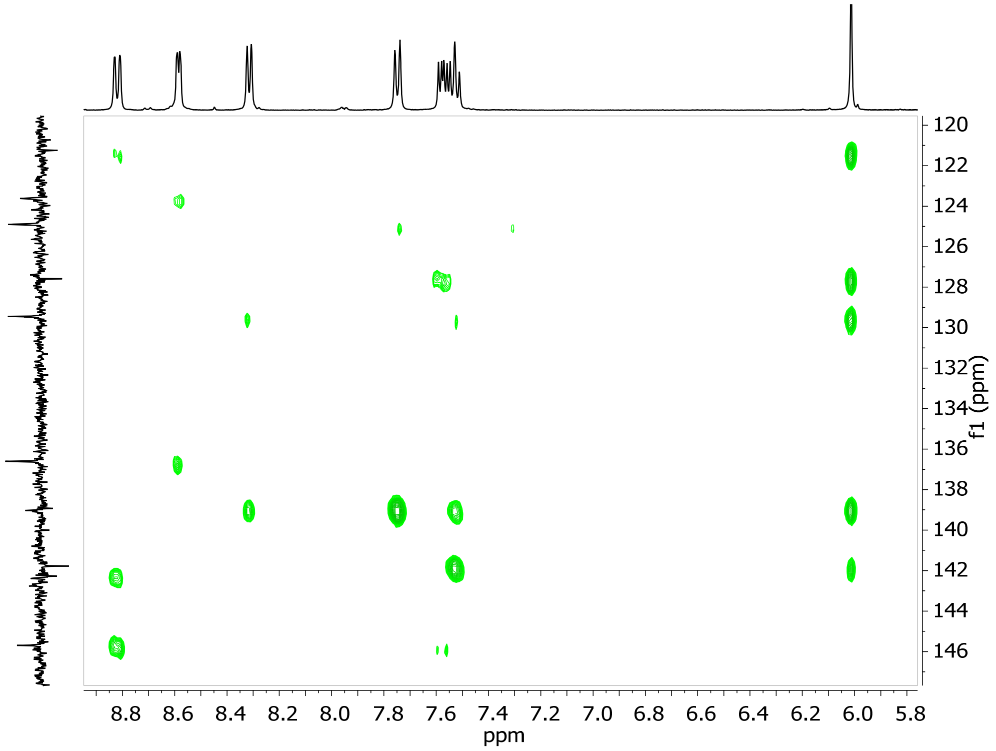

Figure S57. (^1H–^{13}C) HMBC NMR spectrum of 6NPO in D_2O (400 MHz, 25 °C).

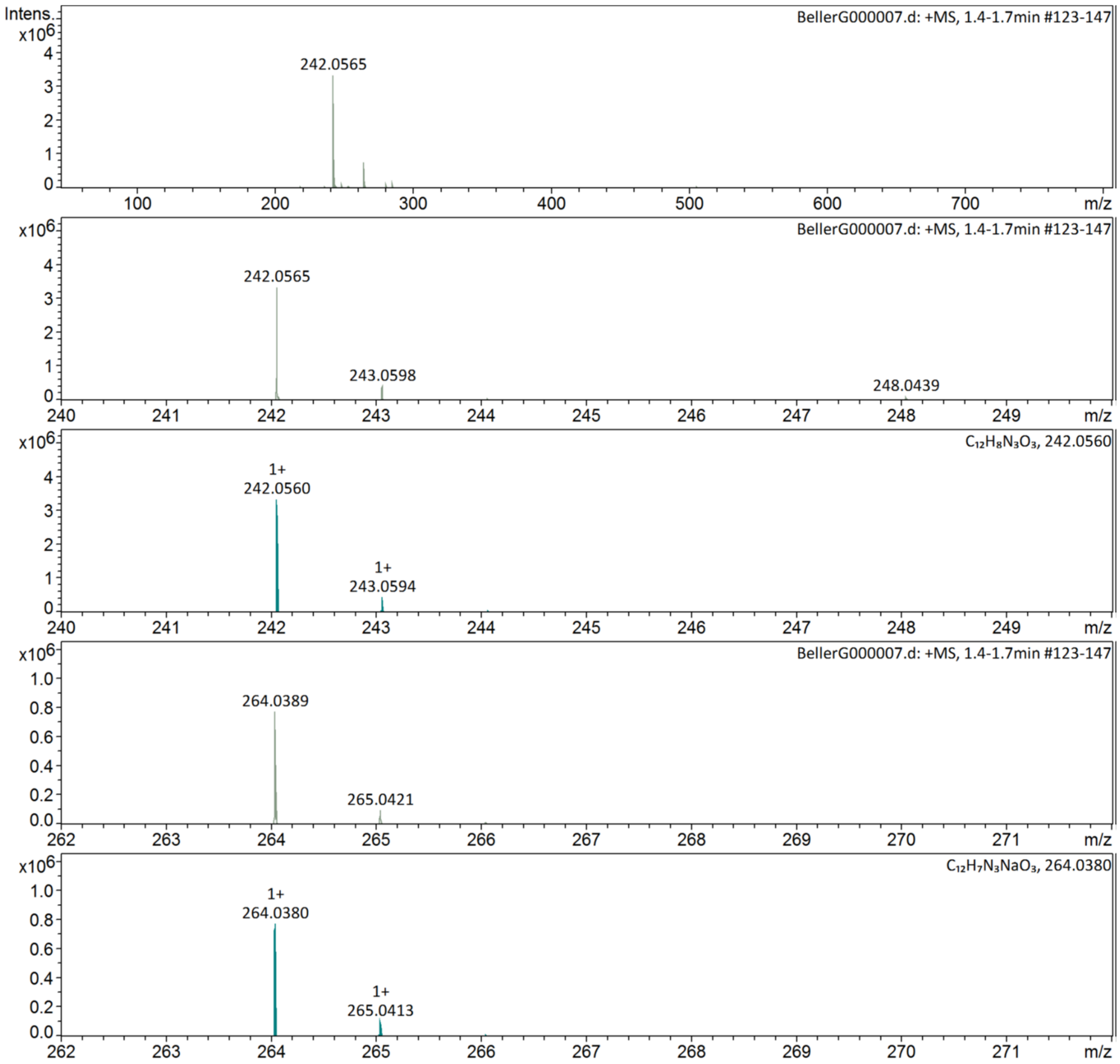

Figure S58. HRMS spectrum of 6NPO in positive mode.

The acid dissociation constants of substituted mono-*N*-oxide derivatives

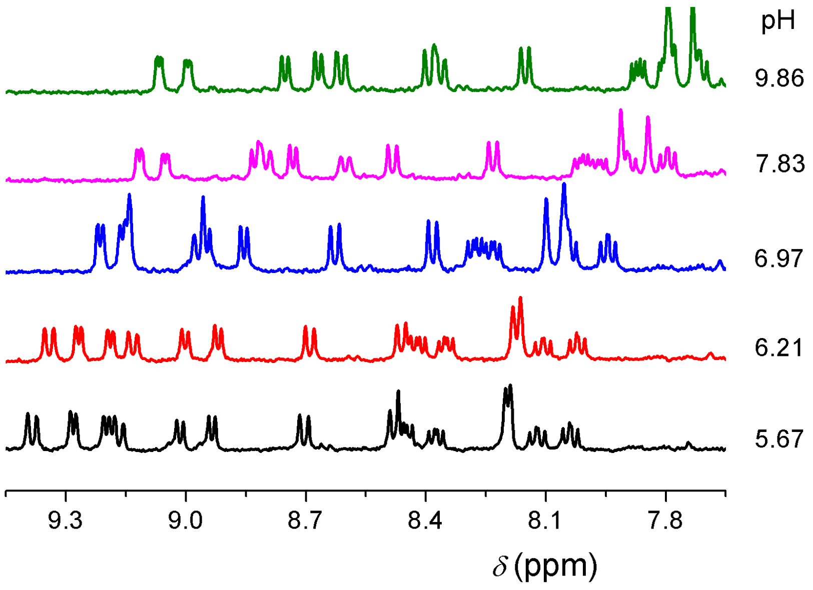

Figure S59. The pH dependence of the aromatic region of the ^{1}H-NMR spectra of 5MPO and 6MPO in H_2O at 400 MHz. $[5MPO+6MPO]_{tot}$ = 8.1 mM, I = 0.10 M; T = 25.0 °C. Only 50% of the total number of spectra are shown for clarity.

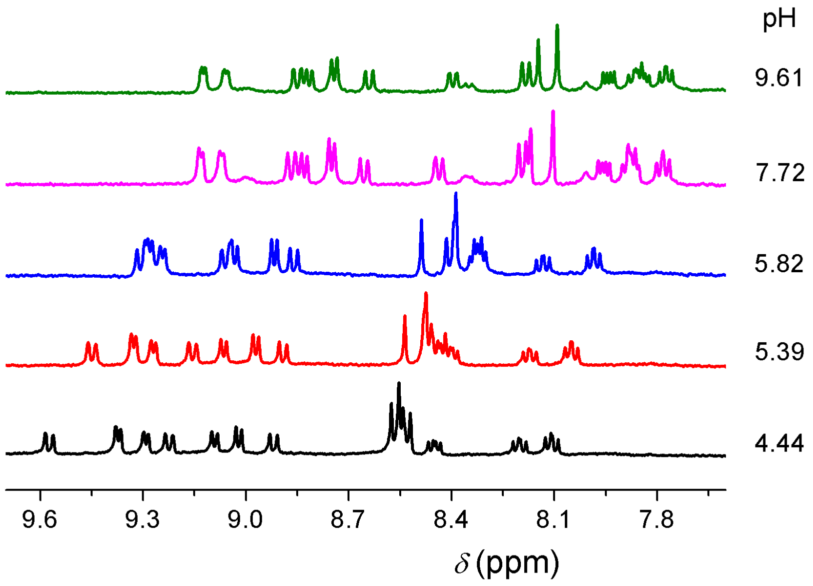

Figure S60. The pH dependence of the ^{1}H-NMR spectra of 5CPO and 6CPO in H_2O at 400 MHz. $[5CPO+6CPO]_{tot}$ = 8.0 mM, I = 0.10 M; T = 25.0 °C. Only 50% of the total number of spectra are shown for clarity.

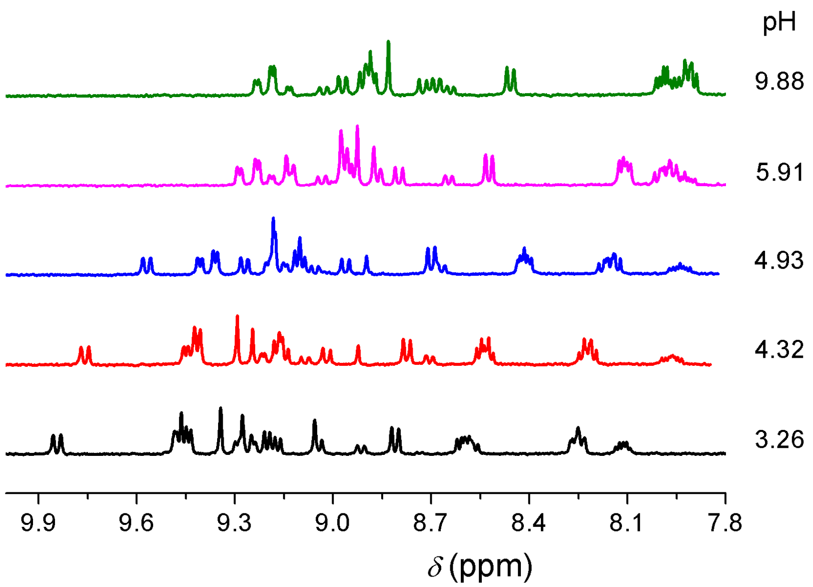

Figure S61. The pH dependence of the ^{1}H-NMR spectra of 5NPO and 6NPO in H_2O at 400 MHz. $[5NPO+6NPO]_{tot}$ = 8.2 mM, *I* = 0.10 M; *T* = 25.0 °C. Only 50% of the total number of spectra are shown for clarity.

X-ray structures of 1,10-phenatroline-1-*N*-oxide derivatives

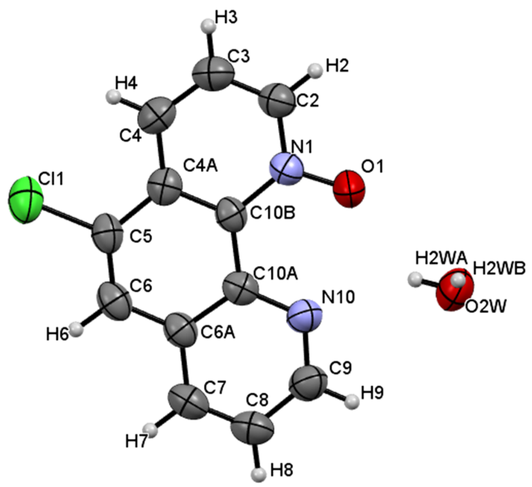

Figure S62. The molecular structure of **5CPO×H₂O** with displacement ellipsoids drawn at 50% probability level.

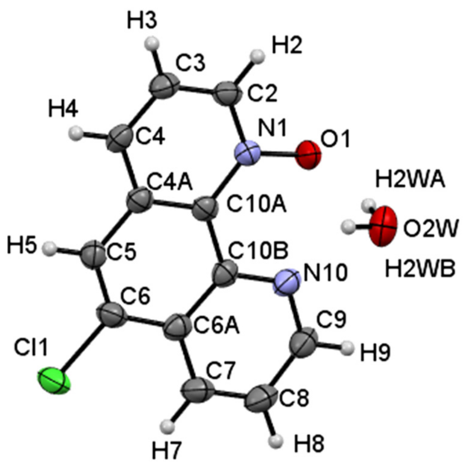

Figure S63. The molecular structure of **6CPO×H_2O** with displacement ellipsoids drawn at 50% probability level.

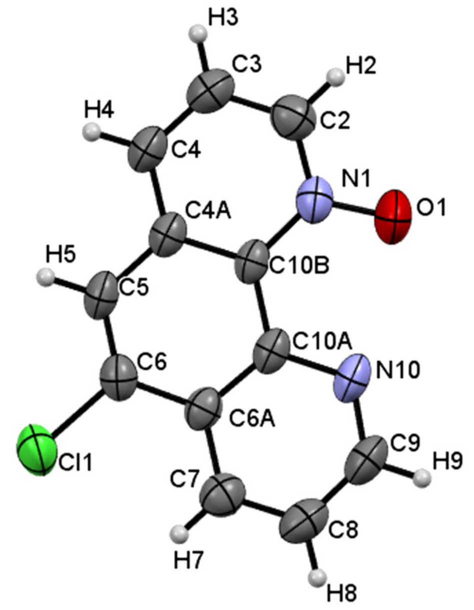

Figure S64. The molecular structure of **6CPO** with displacement ellipsoids drawn at 50% probability level.

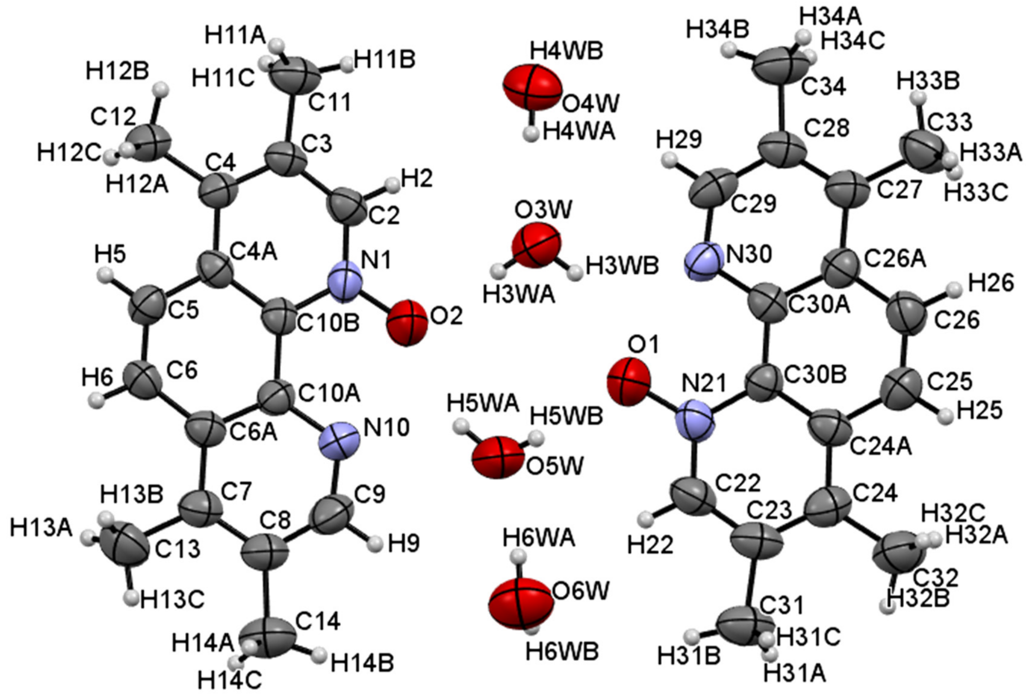

Figure S65. The molecular structure of **TMPO** with displacement ellipsoids drawn at 50% probability level.

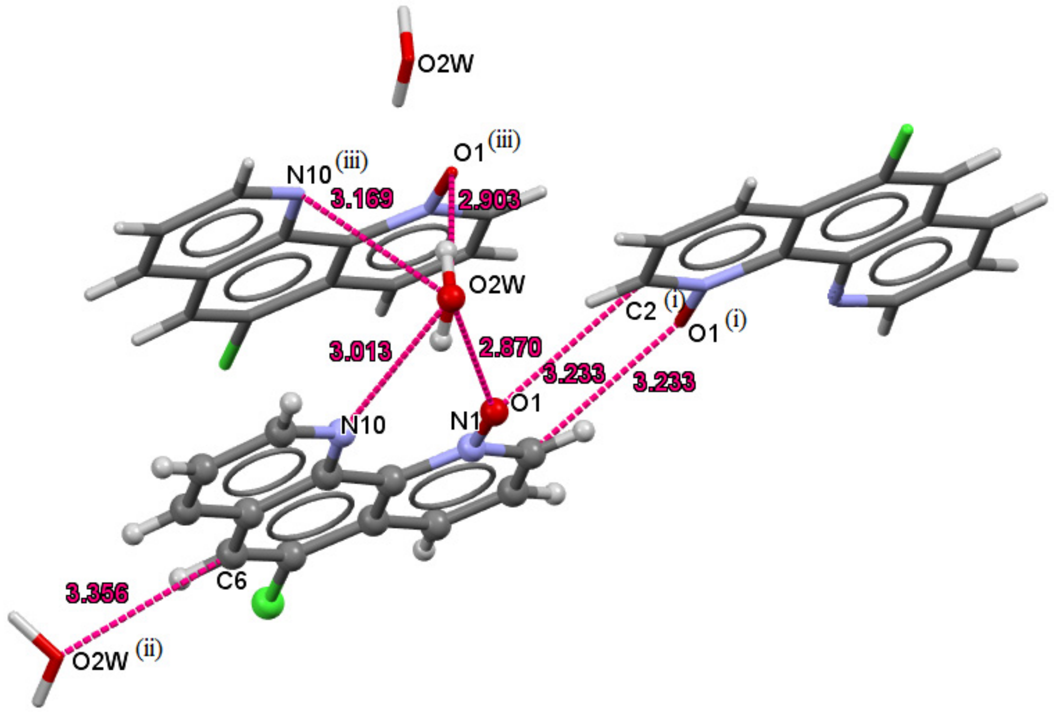

Figure S66. Selected hydrogen bonds in **5CPO×H2O.** The asymmetric unit is given in ball and sticks, and the symmetry-generated part of the compound is given in capped sticks representation.

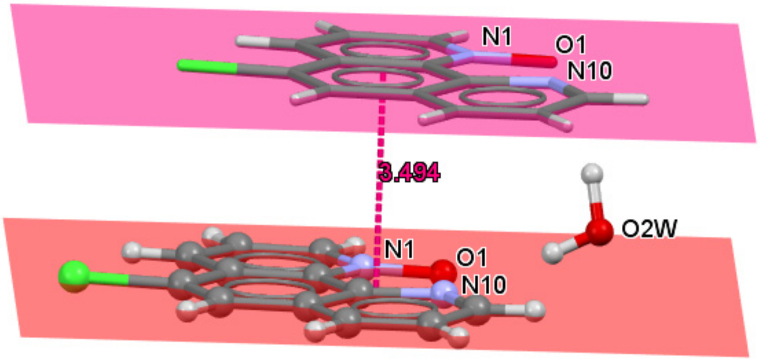

Figure S67. Distance between the aromatic rings of **5CPO×H2O**.

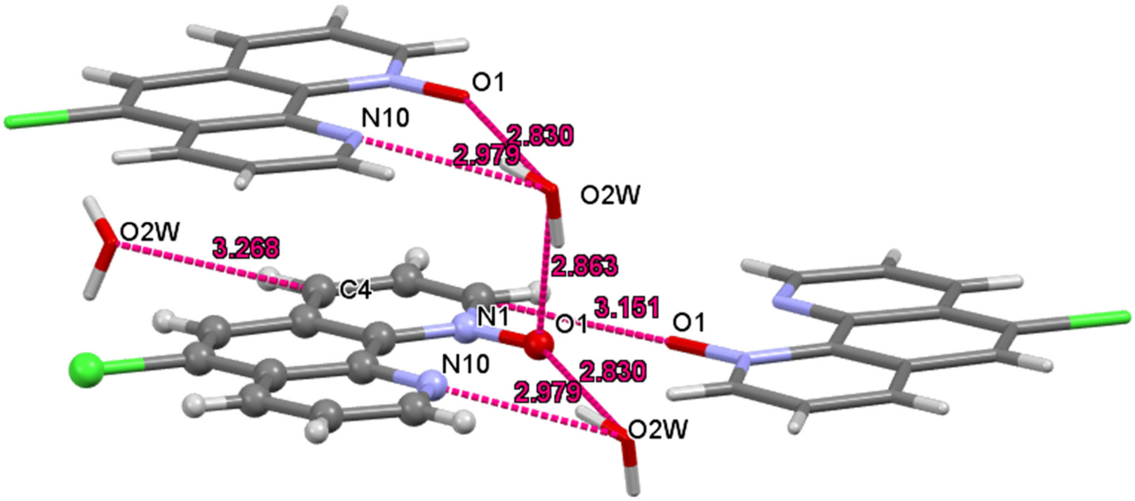

Figure S68. Selected hydrogen bonds in **6CPO×H$_2$O.**
The asymmetric unit is given in ball and sticks, and the symmetry-generated part of the compound is given in capped sticks representation.

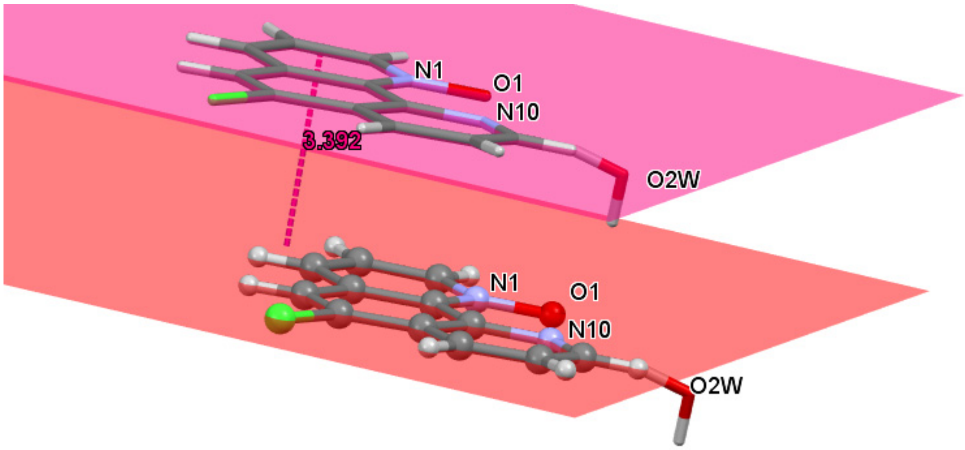

Figure S69. Distance between the aromatic rings of **6CPO×H$_2$O**.

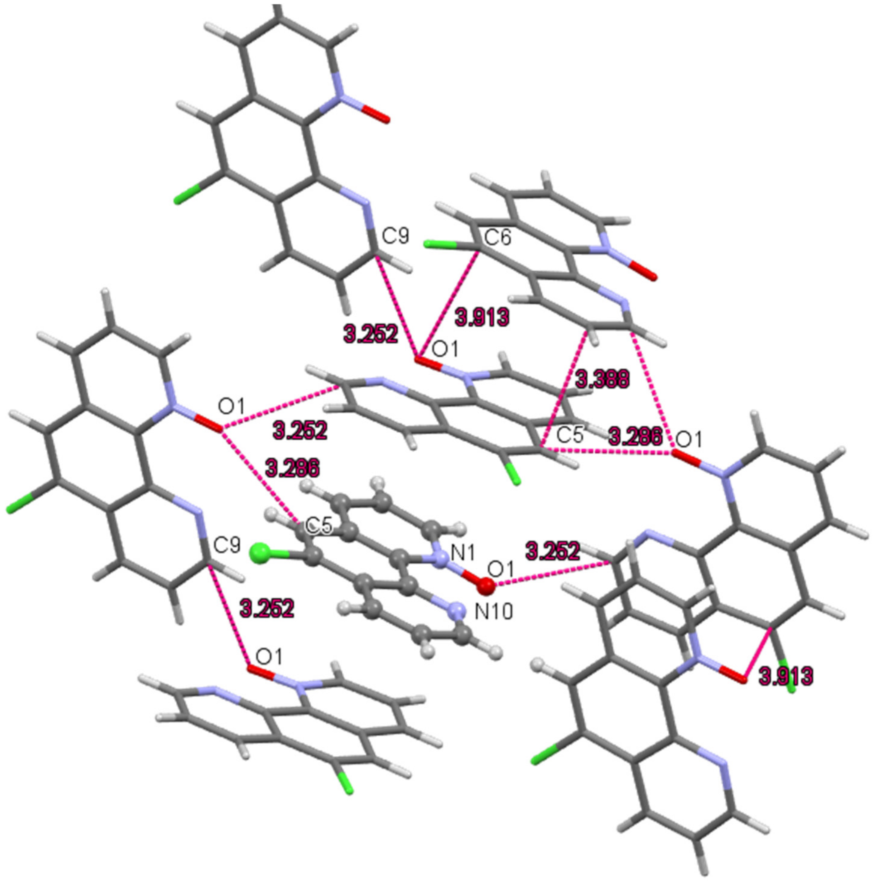

Figure S70. Partial packing diagram with selected weak interactions of **6CPO.**

The asymmetric unit of **6CPO** is given in ball and sticks, and the symmetry-generated part of the compound is given in capped sticks representation.

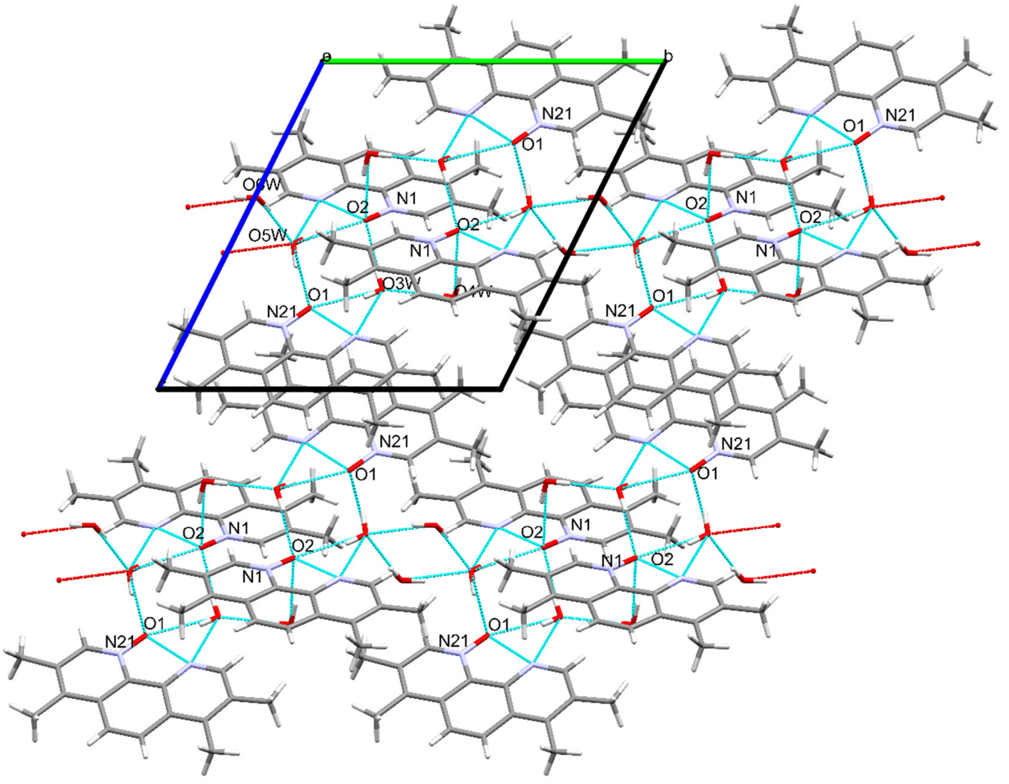

Figure S71. Partial packing view of TMPO along axis "**a**"

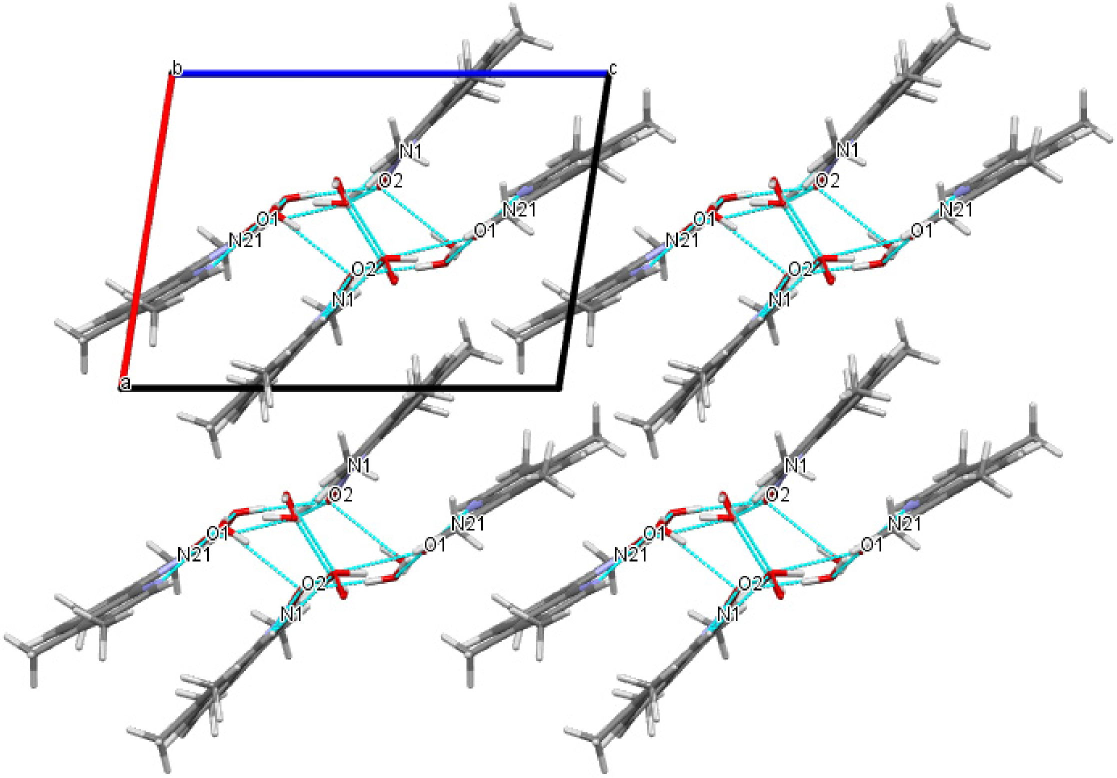

Figure S72. Partial packing view of TMPO along axis "**b**"

Table S1. Crystallographic data of 1,10-phenatroline-mono-*N*-oxdides.

**Compound**	**5CPO×H_2O**	**6CPO×H_2O**	**6CPO**	**2×TMPO×4H_2O**
Empirical formula	$C_{12}H_9ClN_2O$	$C_{12}H_9ClN_2O_2$	$C_{12}H_7ClN_2O$	$C_{32}H_{40}N_4O_6$
M(K)	248.66	248.66	230.65	576.31
T	298(2)	100(2)	298(2)	299.12
Crystal system	monoclinic	Monoclinic	orthorhombic	triclinic
Space group	*P*2$_1$/*c*	*P*2$_1$/*c*	Pbca	P-1
Crystal colour, habit	colourless, needle	colourless, needle	colourless, needle	colourless, block
a[Å]	3.8520(5)	17.7612(15)	14.2028(7)	9.1842(6)
b[Å]	20.096(2)	3.8411(4)	8.7203(4)	12.9797(9)
c [Å]	14.0112(18)	16.1120(14)	15.5475(7)	13.8860(9)
α[°]	90	90		115.642(4)
β [°]	95.414(4)	109.656(3)	90	95.436(4)
γ [°]	90	90		96.332(4)
V[Å^3]	1079.7(2)	1035.15(17)	1925.60(16)	1464.33(18)
Z	4	4	8	2
D_{calcd} [g cm^{-3}]	1.530	1.596	1.591	1.307
μ (mm^{-1})	0.343	0.358	0.371	0.091
F(000)	512.0	512.0	944.0	614.0
Crystal size/mm^3	0.304 × 0.118 × 0.081	0.372 × 0.072 × 0.046	0.376 × 0.103 × 0.061	0.252 × 0.081 × 0.51
Radiation, λ [Å]	MoKα (λ = 0.71073)	MoKα (λ = 0.71073)	MoKα (λ = 0.71073)	MoKα (λ = 0.71073)
2θ-Range [°]	6.748 to 51.762	5.914 to 52.286	5.974 to 52.136	5.246 to 50.802
Index ranges	-4 ≤ h ≤ 4,<br>-24 ≤ k ≤ 24,<br>-17 ≤ l ≤ 16	-21 ≤ h ≤ 21,<br>-4 ≤ k ≤ 4,<br>-19 ≤ l ≤ 19	-17 ≤ h ≤ 17,<br>-10 ≤ k ≤ 10,<br>-19 ≤ l ≤ 19	-11 ≤ h ≤ 11,<br>-15 ≤ k ≤ 15,<br>-16 ≤ l ≤ 16
Number of reflections collected	19759	17581	49701	33460
Number of independent reflections (R_{int})	2075 [R_{int} = 0.1383]	2048 [R_{int} = 0.0954]	1902 [R_{int} = 0.1333]	5382 [R_{int} = 0.1798]
Data/restraints/parameters	2075/0/163	2048/0/157	1902/0/145	5382/0/399
Goodness-of-fit on F^2	1.011	1.060	1.038	1.019

Final R indexes [I>=2σ (I)]	R_1 = 0.0526, wR_2 = 0.1110	R_1 = 0.0444, wR_2 = 0.1081	R_1 = 0.0453, wR_2 = 0.1116	R_1 = 0.0913, wR_2 = 0.2222
Final R indexes [all data]	R_1 = 0.1258, wR_2 = 0.1484	R_1 = 0.0562, wR_2 = 0.1174	R_1 = 0.0709, wR_2 = 0.1315	R_1 = 0.2248, wR_2 = 0.3092
Largest diff. peak/hole / e Å^{-3}	0.23/–0.25	0.39/-0.33	0.22/-0.21	0.54/-0.28
CCDC-number	2075043	2075045	2075044	2075046

Table S2. Bond lengths(Å) for **5CPO×H_2O**.

Atom	Atom	Length	Atom	Atom	Length
Cl1	C5	1.738(4)	C10B	C4A	1.420(5)
O1	N1	1.294(4)	C10A	C6A	1.419(5)
N1	C2	1.346(4)	C6A	C7	1.411(5)
N1	C10B	1.398(4)	C6A	C6	1.418(5)
N10	C10A	1.359(4)	C7	C8	1.356(5)
N10	C9	1.315(4)	C8	C9	1.388(5)
C3	C2	1.368(5)	C4	C4A	1.400(5)
C3	C4	1.363(5)	C4A	C5	1.441(5)
C10B	C10A	1.450(5)	C5	C6	1.336(5)

Table S3. Bond angles(°) for **5CPO×H_2O**.

**Atom**	**Atom**	**Atom**	**Angle**	**Atom**	**Atom**	**Atom**	**Angle**
O1	N1	C2	116.8(3)	C7	C6A	C6	120.8(3)
O1	N1	C10B	122.1(3)	C6	C6A	C10A	121.1(3)
C2	N1	C10B	121.1(3)	C8	C7	C6A	119.5(3)
C9	N10	C10A	117.6(3)	C7	C8	C9	118.1(4)
C4	C3	C2	120.9(4)	C3	C4	C4A	119.1(4)
N1	C2	C3	121.4(4)	C10B	C4A	C5	117.4(3)
N1	C10B	C10A	121.6(3)	C4	C4A	C10B	120.3(3)
N1	C10B	C4A	117.3(3)	C4	C4A	C5	122.3(3)
C4A	C10B	C10A	121.1(3)	C4A	C5	Cl1	118.0(3)
N10	C10A	C10B	121.6(3)	C6	C5	Cl1	119.6(3)
N10	C10A	C6A	121.2(3)	C6	C5	C4A	122.4(3)
C6A	C10A	C10B	117.2(3)	C5	C6	C6A	120.7(3)
C7	C6A	C10A	118.1(3)	N10	C9	C8	125.3(4)

Table S4. Hydrogen bonds (with weak hydrogen bonds) for **5CPO×H_2O**

D	H	A	d(D-H)/Å	d(H-A)/Å	d(D-A)/Å	D-H-A/°
C2	H2	O1(i)	0.93	2.37	3.233(5)	154.4
C6	H6	O2W(ii)	0.93	2.55	3.356(5)	145.1
C7	H7	O2W(ii)	0.93	2.57	3.366(5)	144.0
O2W	H2WA	O1	0.82(5)	2.19(5)	2.870(4)	140(5)
O2W	H2WA	N10	0.82(5)	2.29(5)	3.012(5)	147(5)
O2W	H2WB	O1(iii)	0.93(6)	2.00(6)	2.903(5)	164(5)
O2W	H2WB	N10(iii)	0.93(6)	2.59(6)	3.169(5)	121(4)

[Symmetry codes: (i):1–x,1–y,1–z; (ii): 1+x,1/2–y,-1/2+z; (iii): –1+x,+y,+z]

Table S5. Bond lengths(Å) for **6CPO×H_2O**.

Atom	Atom	Length	Atom	Atom	Length
Cl1	C6	1.745(2)	C10A	C10B	1.450(3)
O1	N1	1.306(2)	C10B	C6A	1.427(3)
N1	C2	1.352(3)	C9	C8	1.396(3)
N1	C10A	1.391(3)	C8	C7	1.370(3)
N10	C9	1.320(3)	C4	C4A	1.401(3)
N10	C10B	1.356(3)	C4A	C5	1.429(3)
C3	C4	1.373(3)	C5	C6	1.341(3)
C3	C2	1.379(3)	C6	C6A	1.430(3)
C10A	C4A	1.416(3)	C6A	C7	1.399(3)

Table S6. Bond angles(°) for **6CPO×H_2O.**

Atom	Atom	Atom	Angle	Atom	Atom	Atom	Angle
O1	N1	C2	116.96(17)	C7	C8	C9	118.2(2)
O1	N1	C10A	122.22(17)	C3	C4	C4A	118.7(2)
C2	N1	C10A	120.81(18)	C4	C4A	C10A	121.32(19)
C9	N10	C10B	118.97(19)	C4	C4A	C5	118.85(19)
C4	C3	C2	119.9(2)	C10A	C4A	C5	119.83(19)
N1	C2	C3	122.02(19)	C6	C5	C4A	120.3(2)
N1	C10A	C4A	117.20(18)	C5	C6	C6A	122.6(2)
N1	C10A	C10B	122.96(18)	C5	C6	Cl1	118.77(17)
C4A	C10A	C10B	119.84(18)	C6A	C6	Cl1	118.61(17)

Table S6. Bond angles(°) for **6CPO×H_2O.**

Atom	Atom	Atom	Angle	Atom	Atom	Atom	Angle
N10	C10B	C6A	120.94(19)	C7	C6A	C10B	117.90(19)
N10	C10B	C10A	120.74(19)	C7	C6A	C6	123.02(19)
C6A	C10B	C10A	118.32(19)	C10B	C6A	C6	119.09(19)
N10	C9	C8	123.8(2)	C8	C7	C6A	120.1(2)

Table S7. Hydrogen bonds (with weak hydrogen bonds) for **6CPO×H_2O**

D	H	A	d(D-H)/Å	d(H-A)/Å	d(D-A)/Å	D-H-A/°
O2W	H2WA	O1$^{(i)}$	0.87	2.07	2.830(2)	146.00
O2W	H2WA	N10$^{(i)}$	0.87	2.26	2.979(3)	140.00
O2W	H2WB	O1	0.87	2.03	2.863(2)	161.00
C2	H2	O1$^{(ii)}$	0.95	2.26	3.151(3)	155.00
C4	H4	O2W$^{(iii)}$	0.95	2.40	3.267(3)	152.00
C5	H5	O2W$^{(iii)}$	0.95	2.55	3.375(3)	146.00

[Symmetry codes: (i): x,–1+y,z, (ii) 1–x,1–y,1–z, (iii): x,–1/2–y,–1/2+z]

Table S8. Bond lengths(Å) for **6CPO.**

Atom	Atom	Length	Atom	Atom	Length
Cl1	C6	1.740(2)	C10A	C10B	1.454(3)
N1	O1	1.284(3)	C6	C5	1.334(3)
N1	C2	1.352(3)	C10B	C4A	1.408(3)
N1	C10B	1.394(3)	C5	C4A	1.426(3)
N10	C9	1.322(3)	C4A	C4	1.395(3)
N10	C10A	1.352(3)	C4	C3	1.359(4)
C6A	C7	1.403(3)	C9	C8	1.385(4)
C6A	C10A	1.419(3)	C7	C8	1.358(4)
C6A	C6	1.433(3)	C2	C3	1.381(4)

Table S9. Bond angles(°) for **6CPO.**

Atom	Atom	Atom	Angle	Atom	Atom	Atom	Angle
O1	N1	C2	116.9(2)	N1	C10B	C4A	117.8(2)
O1	N1	C10B	123.4(2)	N1	C10B	C10A	122.4(2)
C2	N1	C10B	119.7(2)	C4A	C10B	C10A	119.8(2)
C9	N10	C10A	118.2(2)	C6	C5	C4A	120.6(2)
C7	C6A	C10A	117.8(2)	C4	C4A	C10B	121.0(2)
C7	C6A	C6	122.6(2)	C4	C4A	C5	119.0(2)
C10A	C6A	C6	119.6(2)	C10B	C4A	C5	120.0(2)
N10	C10A	C6A	121.3(2)	C3	C4	C4A	119.4(2)
N10	C10A	C10B	120.6(2)	N10	C9	C8	124.5(3)
C6A	C10A	C10B	118.0(2)	C8	C7	C6A	120.0(3)
C5	C6	C6A	122.1(2)	N1	C2	C3	122.5(3)
C5	C6	Cl1	119.70(19)	C7	C8	C9	118.2(3)
C6A	C6	Cl1	118.23(19)	C4	C3	C2	119.5(3

Table S10. Hydrogen bonds (with weak hydrogen bonds) for **6CPO**

D	H	A	d(D-H)/Å	d(H-A)/Å	d(D-A)/Å	D-H-A/°
C5	H5	O1(i)	0.93	2.45	3.286(2)	149.00

[Symmetry codes: (i): 1/2+x,y,3/2–z]

DFT and TD-DFT calculations

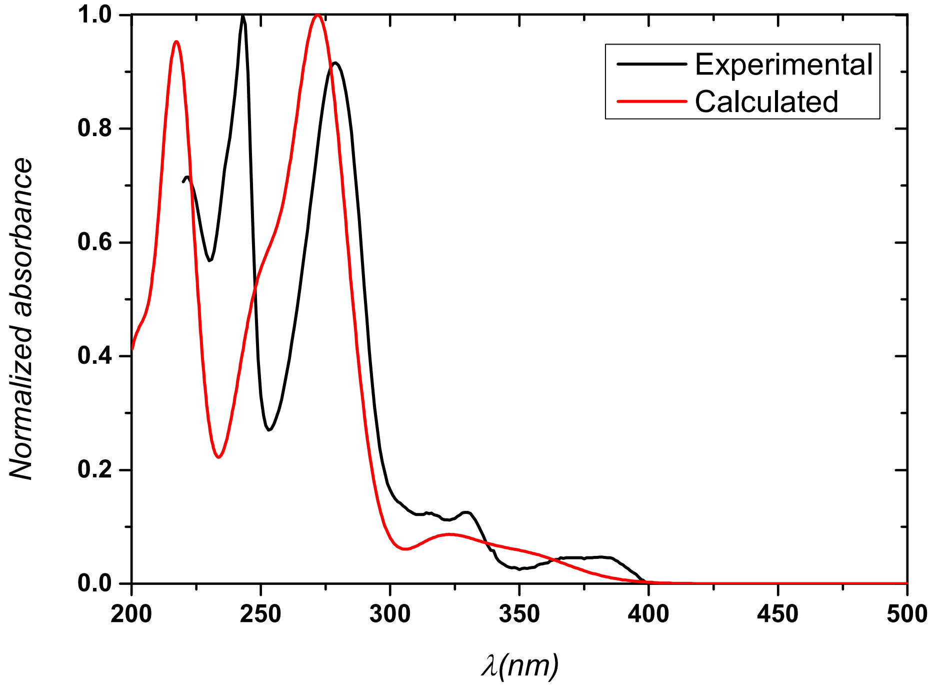

Figure S73. Experimental and calculated UV-VIS spectra of 5CPO using TD-DFT/TPSSh/ *def2*-TZVP level of theory. The presence of water was taken into account due to the PCM model.

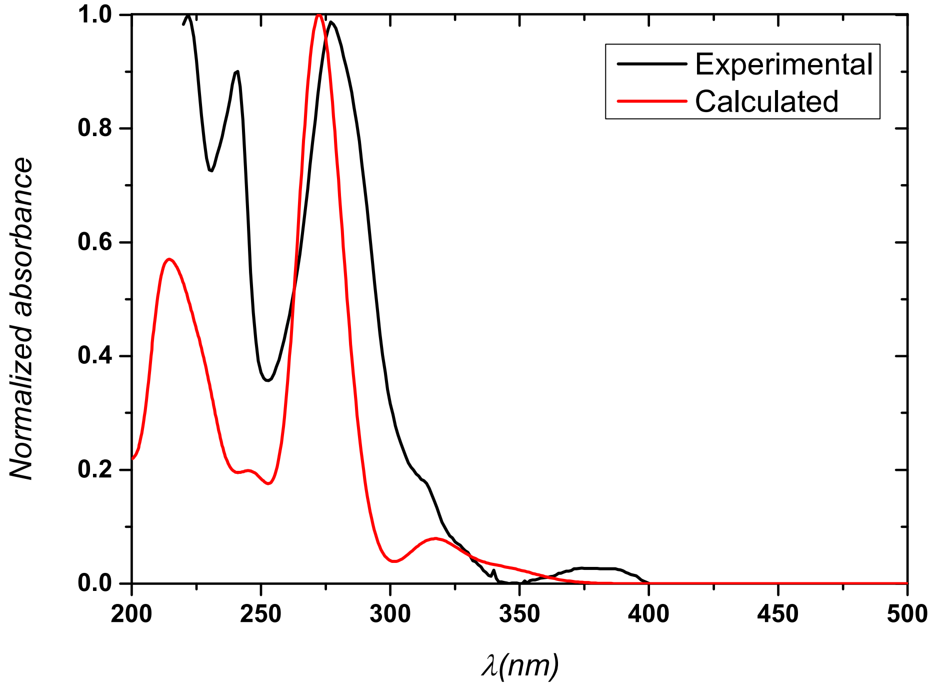

Figure S74. Experimental and calculated UV-VIS spectra of 6CPO using TD-DFT/TPSSh/ *def2*-TZVP level of theory. The presence of water was taken into account due to the PCM model.

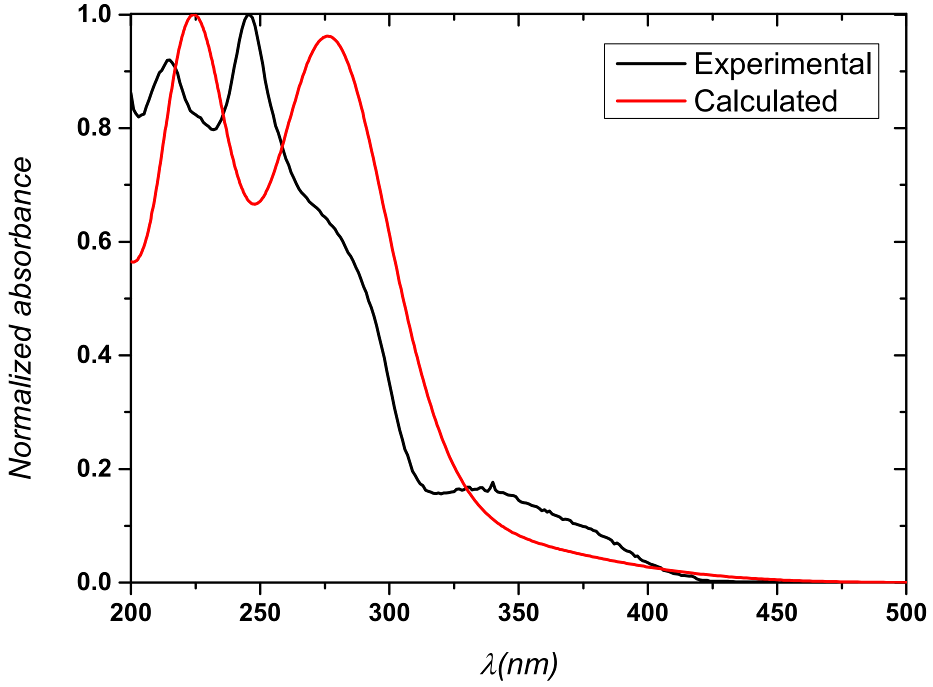

Figure S75. Experimental and calculated UV-VIS spectra of 5MPO using TD-DFT/TPSSh/ *def2*-TZVP level of theory. The presence of water was taken into account due to the PCM model.

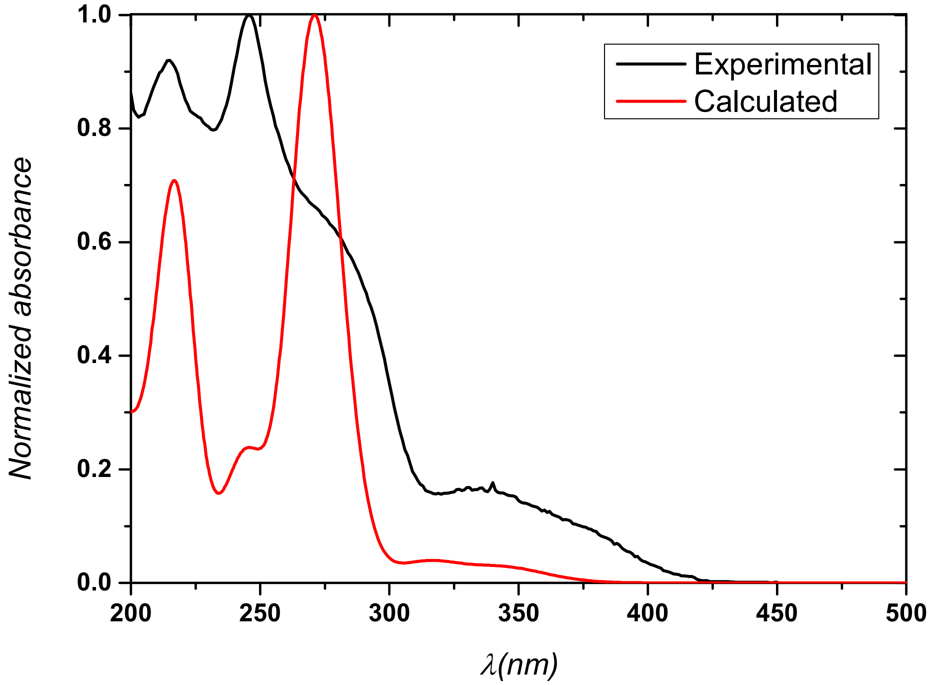

Figure S76. Experimental and calculated UV-VIS spectra of 6MPO using TD-DFT/TPSSh/ *def2*-TZVP level of theory. The presence of water was taken into account due to the PCM model.

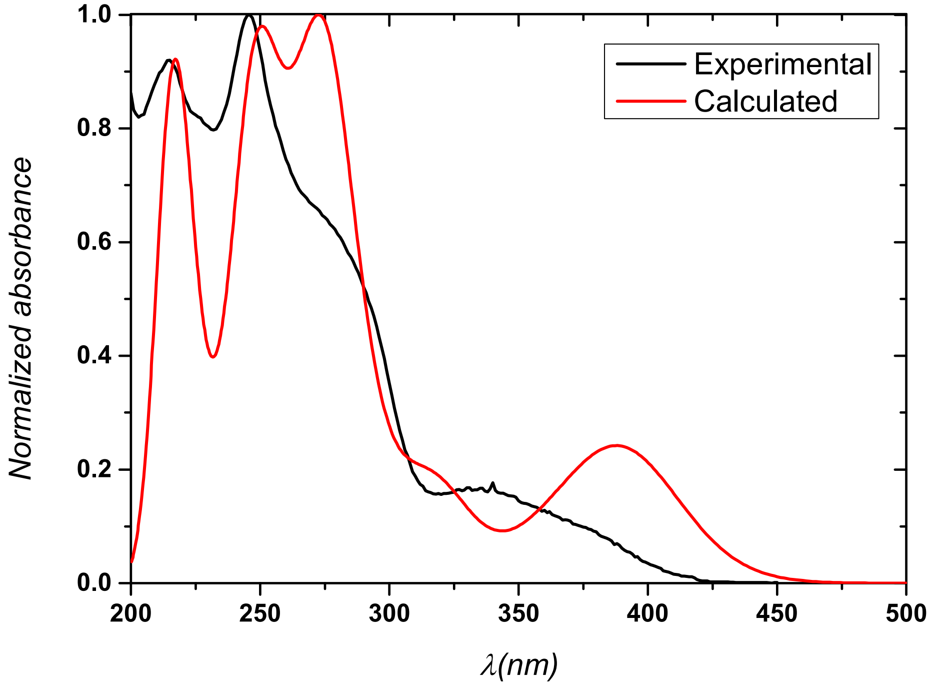

Figure S77. Experimental and calculated UV-VIS spectra of 5NPO using TD-DFT/TPSSh/ *def2*-TZVP level of theory. The presence of water was taken into account due to the PCM model.

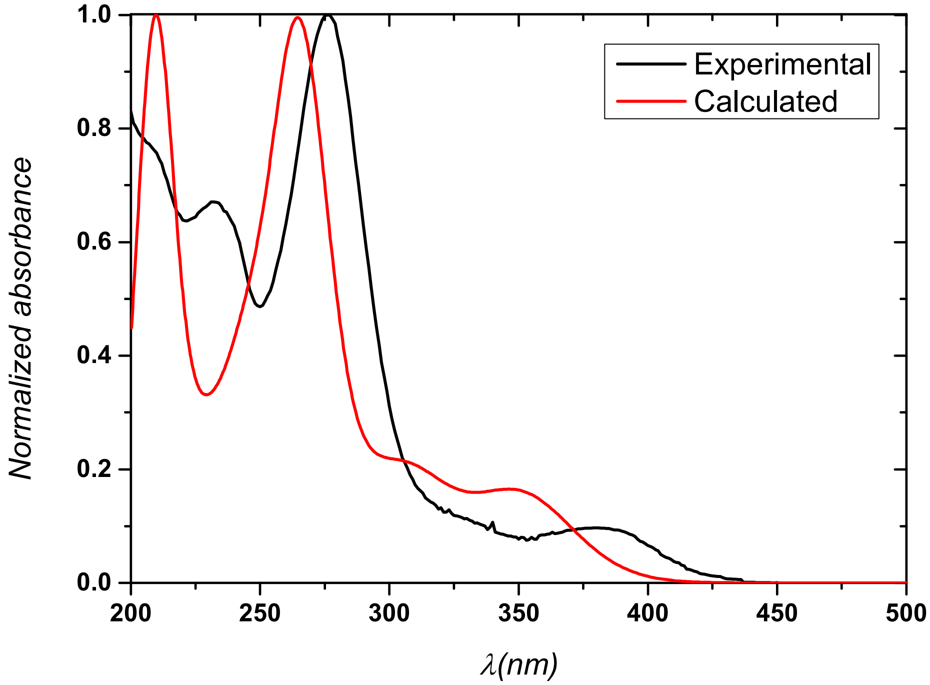

Figure S78. Experimental and calculated UV-VIS spectra of 6NPO using TD-DFT/TPSSh/ *def2*-TZVP level of theory. The presence of water was taken into account due to the PCM model.

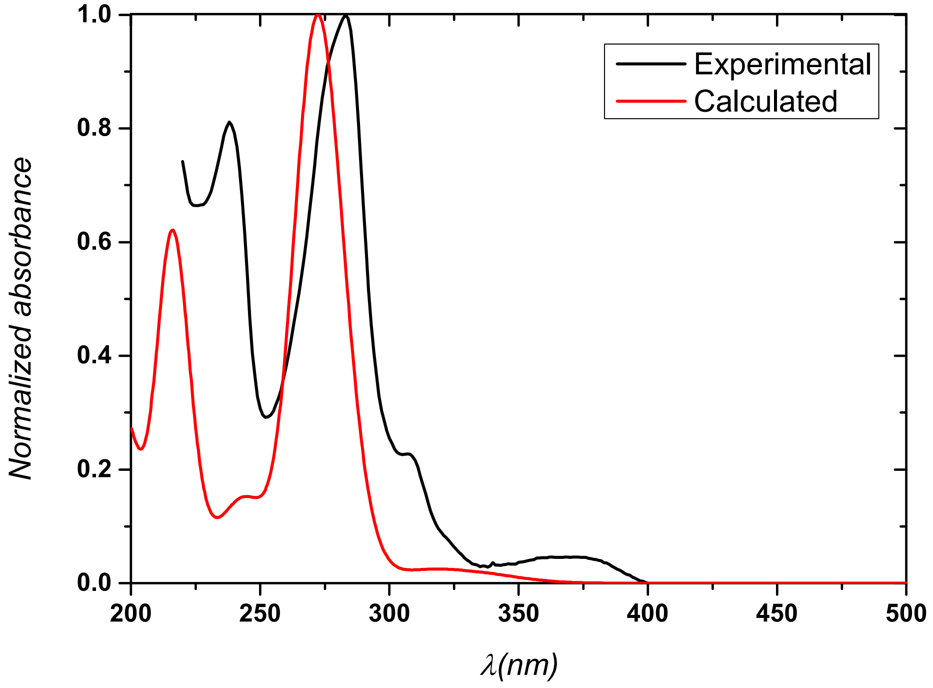

Figure S79. Experimental and calculated UV-VIS spectra of DMPO using TD-DFT/TPSSh/ *def2*-TZVP level of theory. The presence of water was taken into account due to the PCM model.

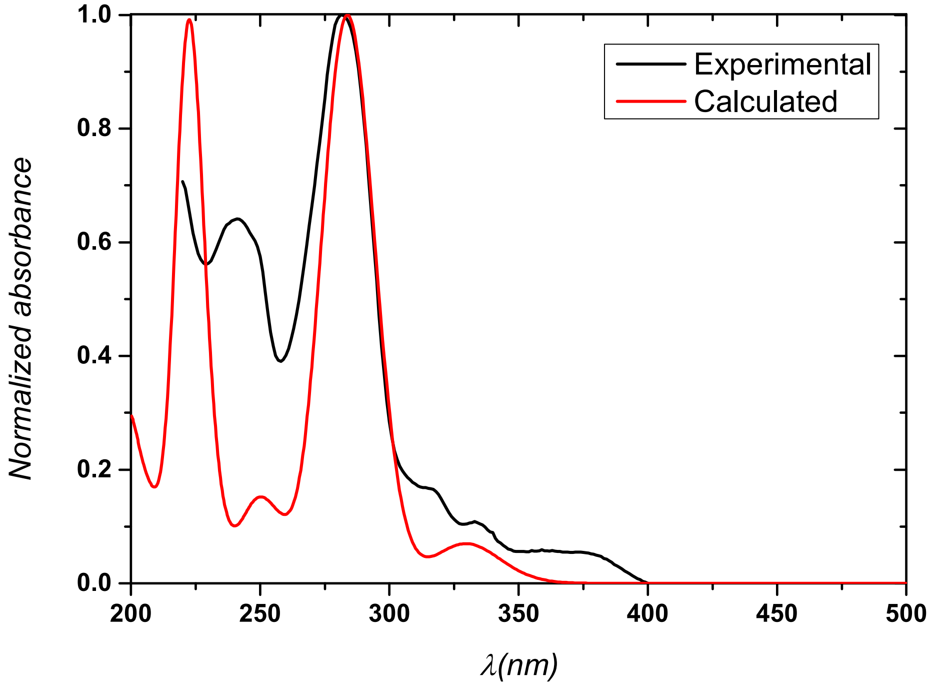

Figure S80. Experimental and calculated UV-VIS spectra of TMPO using TD-DFT/TPSSh/ *def2*-TZVP level of theory. The presence of water was taken into account due to the PCM model.

Table S11. Computed total energies, number of imaginary frequencies and Cartesian coordinates of phenO at B3P86 *def*2-TZVP level of theory.

Electronic Energy (Eh)	-649.31024044
Sum of electronic and zero-point Energies (Eh)	-649.123705
Sum of electronic and thermal Energies (Eh)	-649.113654
Sum of electronic and enthalpy Energies (Eh)	-649.112709
Sum of electronic and thermal Free Energies (Eh)	-649.159405
Number of Imaginary Frequencies	0

Molecular Geometry in Cartesian Coordinates

C	-3.501112	-0.362585	-0.000476
C	-2.932999	0.886720	-0.000067
C	-1.535657	1.018140	0.000246
C	-0.781760	-0.169136	0.000270
C	-2.673320	-1.486402	-0.000455
C	-0.873112	2.278815	0.000300
C	0.643512	-0.082860	0.000336
C	1.280297	1.171840	0.000106
C	0.477979	2.350207	0.000182
C	2.680000	1.223920	-0.000314
H	3.173432	2.186884	-0.000458
C	3.407400	0.059677	-0.000597
C	2.750971	-1.158140	-0.000269
H	-1.476980	3.177482	0.000345
H	-4.574518	-0.491353	-0.000759
H	-3.547267	1.778740	-0.000044
H	-3.084921	-2.487703	-0.000744
H	0.987224	3.305094	0.000088
H	4.487699	0.062464	-0.001061
H	3.247198	-2.117296	-0.000393
N	-1.359051	-1.387799	-0.000016
N	1.422946	-1.204007	0.000276
O	0.860583	-2.427426	0.000696
H	-0.196986	-2.233431	0.000074

Table S12. Computed total energies, number of imaginary frequencies and Cartesian coordinates of DMPO at B3P86 *def*2-TZVP level of theory.

Electronic Energy (Eh)	-725.902894207
Sum of electronic and zero-point Energies (Eh)	-725.659681
Sum of electronic and thermal Energies (Eh)	-725.646149
Sum of electronic and enthalpy Energies (Eh)	-725.645205
Sum of electronic and thermal Free Energies (Eh)	-725.700065
Number of Imaginary Frequencies	0
Mean of alpha and beta Electrons	59

Molecular Geometry in Cartesian Coordinates

C	-3.517638	0.090786	0.000195
C	-2.926295	1.322619	0.000082
C	-1.524386	1.429964	-0.000006
C	-0.800798	0.231292	0.000026
C	-2.721475	-1.067757	0.000223
C	-0.842495	2.683067	-0.000111
C	0.633270	0.290398	-0.000053
C	1.281744	1.528778	-0.000162
C	0.505706	2.728628	-0.000187
C	2.685155	1.555667	-0.000240
H	3.194981	2.509919	-0.000332
C	3.391540	0.388273	-0.000194
C	2.735717	-0.843399	-0.000075
H	-4.594096	-0.009821	0.000272
H	-3.523954	2.225745	0.000067
H	4.471793	0.383815	-0.000245
N	-1.405474	-0.974357	0.000146
N	1.401129	-0.846723	-0.000023
O	0.812931	-2.063752	0.000037
H	-0.214451	-1.866291	0.000028
H	1.035879	3.671713	-0.000262
H	-1.431051	3.591491	-0.000120
C	3.442342	-2.146471	-0.000037
H	3.165925	-2.729569	0.880158
H	3.165969	-2.729562	-0.880258
H	4.515775	-1.977032	-0.000022

C	-3.320340	-2.434034	0.000310
H	-2.992838	-2.987875	-0.881101
H	-2.992084	-2.988114	0.881283
H	-4.407159	-2.383699	0.000760

Table S13. Computed total energies, number of imaginary frequencies and Cartesian coordinates of TMPO at B3P86 *def*2-TZVP level of theory.

Electronic Energy (Eh)	-804.53670418
Sum of electronic and zero-point Energies (Eh)	-804.237059
Sum of electronic and thermal Energies (Eh)	-804.220366
Sum of electronic and enthalpy Energies (Eh)	-804.219422
Sum of electronic and thermal Free Energies (Eh)	-804.280726
Number of Imaginary Frequencies	0
Mean of alpha and beta Electrons	67

Molecular Geometry in Cartesian Coordinates

C	-3.520540	-0.492349	0.000076
C	-2.908199	0.749709	-0.000082
C	-1.492033	0.804666	-0.000051
C	-0.777079	-0.398007	0.000001
C	-2.706946	-1.632162	0.000185
C	-0.764288	2.035511	-0.000010
C	0.660984	-0.366023	-0.000022
C	1.356899	0.849695	0.000033
C	0.583980	2.055135	0.000073
C	2.771925	0.869361	0.000053
C	3.454561	-0.338714	-0.000036
C	2.720602	-1.513570	-0.000121
N	-1.394197	-1.595483	0.000117
N	1.396931	-1.513103	-0.000080
O	0.810672	-2.735255	-0.000112
H	-0.205451	-2.533750	0.000014
H	1.102554	3.002283	0.000165
H	-1.307173	2.969785	0.000001
H	-3.158656	-2.617158	0.000318

H	3.175669	-2.493212	-0.000227
C	-3.700474	2.019280	-0.000261
H	-3.460837	2.620343	0.879312
H	-3.459824	2.620785	-0.879248
H	-4.770107	1.830983	-0.000931
C	-5.008912	-0.669454	0.000106
H	-5.461039	-0.208779	0.880155
H	-5.460961	-0.209472	-0.880355
H	-5.266209	-1.727785	0.000492
C	4.949919	-0.442408	-0.000044
H	5.261438	-1.485457	-0.000161
H	5.372345	0.042609	-0.881133
H	5.372284	0.042366	0.881220
C	3.500148	2.174092	0.000167
H	3.230743	2.761952	-0.879544
H	3.230533	2.761888	0.879857
H	4.576900	2.036186	0.000292

Table S14. Computed total energies, number of imaginary frequencies and Cartesian coordinates of 7MPO at B3P86 *def2*-TZVP level of theory.

Electronic Energy (Eh)	-686.574988522
Sum of electronic and zero-point Energies (Eh)	-686.358271
Sum of electronic and thermal Energies (Eh)	-686.346768
Sum of electronic and enthalpy Energies (Eh)	-686.345824
Sum of electronic and thermal Free Energies (Eh)	-686.395606
Number of Imaginary Frequencies	0
Mean of alpha and beta Electrons	55

Molecular Geometry in Cartesian Coordinates

C	3.057030	1.128577	-0.006520
C	2.765888	-0.215471	-0.003080
C	1.398976	-0.593916	-0.001399
C	0.436345	0.425133	-0.000996
C	2.031988	2.072720	-0.005011
C	0.979323	-1.961040	-0.000238
C	-0.955473	0.066780	0.000217
C	-1.341400	-1.280454	-0.001413
C	-0.328694	-2.287673	-0.001197
C	-2.704948	-1.606368	-0.002828
C	-3.645997	-0.611234	-0.002049

C	-3.234487	0.710142	0.001102
N	0.761314	1.735456	-0.001730
N	-1.943770	1.012684	0.002390
O	-1.648683	2.331649	0.007864
H	-0.609803	2.366510	0.003315
H	-0.645583	-3.322036	-0.001785
H	1.729115	-2.739489	0.000599
H	2.253391	3.132516	-0.006091
H	-3.908838	1.553487	0.003169
H	4.084221	1.467197	-0.009367
H	-2.997220	-2.648068	-0.004591
H	-4.704707	-0.822643	-0.003274
C	3.853697	-1.239390	0.007962
H	3.816289	-1.826944	0.927914
H	3.747118	-1.935580	-0.825672
H	4.829184	-0.761947	-0.059051

Table S15. Computed total energies, number of imaginary frequencies and Cartesian coordinates of 4MPO at B3P86 *def2*-TZVP level of theory.

Electronic Energy (Eh)	-686.575280874
Sum of electronic and zero-point Energies (Eh)	-686.358704
Sum of electronic and thermal Energies (Eh)	-686.347016
Sum of electronic and enthalpy Energies (Eh)	-686.346072
Sum of electronic and thermal Free Energies (Eh)	-686.396441
Number of Imaginary Frequencies	0
Mean of alpha and beta Electrons	55

Molecular Geometry in Cartesian Coordinates

C	-3.792588	-0.296722	0.000150
C	-2.959141	-1.382414	0.000140
C	-1.566907	-1.191820	0.000101

C	-1.096658	0.127654	0.000063
C	-3.232729	0.983785	0.000123
C	-0.641217	-2.277054	0.000096
C	0.322859	0.357730	-0.000007
C	1.220154	-0.720475	-0.000053
C	0.687601	-2.049992	0.000027
C	2.616421	-0.477483	-0.000171
C	3.044548	0.831155	-0.000250
C	2.136055	1.869336	-0.000181
N	-1.933614	1.187290	0.000078
N	0.835658	1.624905	-0.000052
O	0.032570	2.715899	0.000036
H	-0.925196	2.332240	0.000037
H	1.377232	-2.881728	0.000030
H	-1.031335	-3.286682	0.000150
H	-3.861038	1.865537	0.000138
H	2.412972	2.913058	-0.000211
H	-4.867148	-0.411384	0.000179
H	4.097348	1.073922	-0.000368
H	-3.354488	-2.390331	0.000162
C	3.600450	-1.599056	-0.000112
H	3.463244	-2.229827	0.880155
H	3.462562	-2.230741	-0.879599
H	4.617887	-1.214496	-0.000712

Table S16. Computed total energies, number of imaginary frequencies and Cartesian coordinates of 5MPO at B3P86 *def2*-TZVP level of theory.

Electronic Energy (Eh)	-686.573224657
Sum of electronic and zero-point Energies (Eh)	-686.356294
Sum of electronic and thermal Energies (Eh)	-686.344778
Sum of electronic and enthalpy Energies (Eh)	-686.343834
Sum of electronic and thermal Free Energies (Eh)	-686.393574
Number of Imaginary Frequencies	0

Mean of alpha and beta Electrons	55

Molecular Geometry in Cartesian Coordinates

C	-3.650961	0.139172	-0.000119
C	-2.826230	1.232277	-0.000148
C	-1.432401	1.053805	-0.000111
C	-0.956665	-0.261627	-0.000045
C	-3.083208	-1.137684	-0.000054
C	-0.518353	2.149203	-0.000124
C	0.464276	-0.469407	0.000009
C	1.348052	0.622474	0.000012
C	0.821394	1.968489	-0.000062
C	2.727541	0.369336	0.000101
C	3.198714	-0.918276	0.000202
C	2.300765	-1.967239	0.000195
N	-1.782057	-1.329255	-0.000022
N	0.996966	-1.727777	0.000089
O	0.204900	-2.823469	0.000059
H	-0.761231	-2.450968	0.000060
H	-3.704314	-2.024341	-0.000033
H	2.580941	-3.009896	0.000258
H	-4.726340	0.246455	-0.000142
H	4.255595	-1.138260	0.000288
H	-3.231004	2.236449	-0.000200
H	3.424753	1.194742	0.000098
H	-0.931514	3.150467	-0.000182
C	1.762012	3.132620	-0.000032
H	2.408267	3.118428	0.880175
H	2.408492	3.118334	-0.880066
H	1.203174	4.066705	-0.000147

Table S17. Computed total energies, number of imaginary frequencies and Cartesian coordinates of 6MPO at B3P86 *def2*-TZVP level of theory.

Electronic Energy (Eh)	-686.573803809
Sum of electronic and zero-point Energies (Eh)	-686.357461
Sum of electronic and thermal Energies (Eh)	-686.345606
Sum of electronic and enthalpy Energies (Eh)	-686.344662
Sum of electronic and thermal Free Energies (Eh)	-686.396141
Number of Imaginary Frequencies	0
Mean of alpha and beta Electrons	55

Molecular Geometry in Cartesian Coordinates

C	3.098035	-1.484542	0.000068
C	2.871069	-0.133631	0.000055
C	1.555007	0.361643	-0.000004
C	0.525196	-0.591618	-0.000044
C	2.007059	-2.353630	0.000038
C	1.253398	1.772870	0.000000
C	-0.839436	-0.144016	-0.000064
C	-1.122981	1.226743	0.000001
C	-0.042269	2.160516	0.000019
C	-2.460495	1.648814	0.000052
C	-3.470034	0.722676	0.000039
C	-3.156429	-0.624863	-0.000044
N	0.765910	-1.919186	-0.000005
N	-1.890384	-1.017208	-0.000099
O	-1.689240	-2.354337	-0.000186
H	-0.661319	-2.462714	-0.000365
H	-0.296931	3.212918	0.000053
H	2.147572	-3.426966	0.000053
H	-3.889687	-1.417302	-0.000067
H	4.101814	-1.884965	0.000108
H	-4.510597	1.010457	0.000082
H	3.705024	0.554824	0.000098
H	-2.678856	2.708532	0.000099
C	2.365372	2.773628	0.000146
H	3.000303	2.650504	0.880021
H	3.000641	2.650435	-0.879471
H	1.966320	3.786188	0.000028

Table S18. Computed total energies, number of imaginary frequencies and Cartesian coordinates of 5CPO at B3P86 *def*2-TZVP level of theory.

Electronic Energy (Eh)	-1106.8566487
Sum of electronic and zero-point Energies (Eh)	-1106.67764
Sum of electronic and thermal Energies (Eh)	-1106.666371
Sum of electronic and enthalpy Energies (Eh)	-1106.665427
Sum of electronic and thermal Free Energies (Eh)	-1106.715422
Number of Imaginary Frequencies	0
Mean of alpha and beta Electrons	59

Molecular Geometry in Cartesian Coordinates

C	3.572871	-1.234061	0.000073
C	2.421832	-1.974736	0.000015
C	1.177807	-1.322208	-0.000032
C	1.182697	0.076513	-0.000029
C	3.480199	0.160041	0.000076
C	-0.054635	-2.038803	-0.000047
C	-0.076478	0.769459	-0.000043
C	-1.290725	0.062556	-0.000002
C	-1.226109	-1.375936	-0.000025
C	-2.495272	0.777340	0.000071
H	-3.437357	0.248874	0.000103
C	-2.481052	2.147449	0.000110
C	-1.272760	2.815282	0.000050
H	-0.026315	-3.120151	-0.000062
H	4.544987	-1.705579	0.000113
H	2.452700	-3.056841	0.000010
H	4.369848	0.776709	0.000120
H	-3.393809	2.723657	0.000181
H	-1.169066	3.890030	0.000059
N	2.325968	0.790430	0.000022
N	-0.134916	2.134830	-0.000041
O	0.988914	2.883648	-0.000160
H	1.765868	2.199339	-0.000073
Cl	-2.705046	-2.275260	-0.000020

Table S19. Computed total energies, number of imaginary frequencies and Cartesian coordinates of 6CPO at B3P86 *def*2-TZVP level of theory.

Electronic Energy (Eh)	-1106.85804283
Sum of electronic and zero-point Energies (Eh)	-1106.678679
Sum of electronic and thermal Energies (Eh)	-1106.66741
Sum of electronic and enthalpy Energies (Eh)	-1106.666465
Sum of electronic and thermal Free Energies (Eh)	-1106.716545
Number of Imaginary Frequencies	0
Mean of alpha and beta Electrons	59

Molecular Geometry in Cartesian Coordinates

C	2.361634	2.422142	0.000260
C	2.516267	1.061851	0.000062
C	1.384456	0.231241	-0.000044
C	0.128546	0.859039	-0.000019
C	1.073704	2.958583	0.000332
C	1.437386	-1.204106	-0.000074
C	-1.063167	0.055475	-0.000075
C	-0.969356	-1.340397	0.000085
C	0.319513	-1.954261	0.000046
C	-2.140013	-2.111894	0.000264
H	-2.061007	-3.190682	0.000363
C	-3.361775	-1.493428	0.000341
C	-3.423383	-0.111185	0.000094
H	3.217581	3.081690	0.000358
H	3.505376	0.625898	0.000002
H	0.912555	4.028805	0.000515
H	-4.286084	-2.051107	0.000546
H	-4.343000	0.454540	0.000026
N	-0.000567	2.200219	0.000172
N	-2.311338	0.610556	-0.000173
O	-2.483610	1.950498	-0.000765
H	-1.527328	2.337382	-0.000027
H	0.369449	-3.034275	0.000109
Cl	2.981283	-1.986472	-0.000200

Table S20. Computed total energies, number of imaginary frequencies and Cartesian coordinates of 5NPO at B3P86 *def2*-TZVP level of theory.

Electronic Energy (Eh)	-851.759349198
Sum of electronic and zero-point Energies (Eh)	-851.567991
Sum of electronic and thermal Energies (Eh)	-851.556301
Sum of electronic and enthalpy Energies (Eh)	-851.555356
Sum of electronic and thermal Free Energies (Eh)	-851.606237
Number of Imaginary Frequencies	1
Mean of alpha and beta Electrons	62

Molecular Geometry in Cartesian Coordinates

C	3.267683	-2.065786	0.000047
C	1.957533	-2.462667	-0.000019
C	0.945577	-1.489681	-0.000029
C	1.321050	-0.145495	0.000003
C	3.553639	-0.699745	0.000118
C	-0.429057	-1.836566	-0.000058
C	0.291963	0.864525	-0.000010
C	-1.077955	0.526239	0.000008
C	-1.387777	-0.888438	-0.000033
C	-2.016817	1.572394	0.000081
H	-3.068872	1.356174	0.000109
C	-1.603228	2.878177	0.000121
C	-0.257440	3.172487	0.000067
H	-0.697260	-2.882570	-0.000086
H	4.075747	-2.782796	0.000064
H	1.686062	-3.510294	-0.000053
H	4.576171	-0.344976	0.000190
H	-2.314761	3.690001	0.000176
H	0.152017	4.171347	0.000063
N	2.614390	0.224663	0.000097
N	0.631691	2.190332	-0.000009
O	1.921045	2.589854	-0.000143
H	2.476188	1.712826	-0.000399
N	-2.770073	-1.400667	-0.000059
O	-2.924894	-2.604467	0.000105
O	-3.697197	-0.618221	-0.000218

Table S21. Computed total energies, number of imaginary frequencies and Cartesian coordinates of 6CPO at B3P86 *def*2-TZVP level of theory.

Electronic Energy (Eh)	-851.762941246
Sum of electronic and zero-point Energies (Eh)	-851.571386
Sum of electronic and thermal Energies (Eh)	-851.558851
Sum of electronic and enthalpy Energies (Eh)	-851.557907
Sum of electronic and thermal Free Energies (Eh)	-851.610741
Number of Imaginary Frequencies	0
Mean of alpha and beta Electrons	62

Molecular Geometry in Cartesian Coordinates

C	1.748773	2.878346	0.120291
C	2.138497	1.566548	0.092942
C	1.165585	0.553778	0.023816
C	-0.179609	0.957564	0.014738
C	0.389490	3.186954	0.080408
C	1.430207	-0.855770	0.015679
C	-1.223883	-0.033124	-0.006488
C	-0.910726	-1.396109	0.004117
C	0.460572	-1.785794	0.024827
C	-1.934938	-2.351141	-0.014916
H	-1.679659	-3.402141	-0.009354
C	-3.241703	-1.939949	-0.039663
C	-3.524063	-0.586432	-0.046769
H	2.478819	3.672937	0.173818
H	3.188220	1.317904	0.128092
H	0.046173	4.213205	0.092456
H	-4.062507	-2.640956	-0.053798
H	-4.523345	-0.177633	-0.066956
N	-0.536998	2.255769	0.035995
N	-2.545243	0.310751	-0.030809
O	-2.937187	1.602693	-0.045179
H	-2.056984	2.141688	-0.007172
H	0.698362	-2.840548	0.021536
N	2.809989	-1.350519	-0.044208
O	3.093252	-2.316985	0.629924
O	3.582368	-0.781419	-0.787165

Table S22. Calculated and experimental transitions for some *N*-oxides.

	λ_{calc}	Transition (weight%)	Main character	λ_{exp}	APD
phenO	334	HOMO → LUMO (66.3)	C(p_z) (83%) → C(p_z) (76%)	318	4.9
	269	H–1 → LUMO (45.2)	C(p_z) (90%) → C(p_z) (76%)	268	0.2
	265	H–2 → L+1 (53.1)	N(p_z) (48%) → C(p_z) (90%)		
	245	H–4 → LUMO (56.9)	C(p_z) (92%) → C(p_z) (76%)	240	2.0
TMPO	335	HOMO → LUMO (56.7)	C(p_z) (75%) → C(p_z) (76%)	333	0.5
	288	H–1 → LUMO (47.5)	C(p_z) (78%) → C(p_z) (76%)	283	1.7
	278	H–1 → L+1 (63.1)	C(p_z) (78%) → C(p_z) (81%)		
	258	H–3 → LUMO (58.2)	C(p_z) (63%) → C(p_z) (76%)	244	5.5
DMPO	344	HOMO → LUMO (66.5)	C(p_z) (78%) → C(p_z) (74%)	315	9.1
	277	H–1 → LUMO (52.7)	C(p_z) (91%) → C(p_z) (74%)	271	2.2
	253	H–3 → LUMO (63.7)	O(p_z) (35%) → C(p_z) (74%)	240	5.2
5CPO	353	HOMO → LUMO (67.7)	C(p_z) (77%) → C(p_z) (74%)	376	6.1
	321	HOMO → L+1 (58.4)	C(p_z) (77%) → C(p_z) (88%)	331	3.2
	274	H–1 → LUMO (49.6)	C(p_z) (88%) → C(p_z) (74%)	277	1.0
	269	H–1 → L+1 (50.1)	C(p_z) (88%) → C(p_z) (88%)		
	252	H–3 → L+1 (53.5)	C(p_z) (87%) → C(p_z) (88%)		
	251	H–3 → LUMO (56.4)	C(p_z) (87%) → C(p_z) (74%)	244	2.8
6CPO	344	HOMO → LUMO (64.7)	C(p_z) (74%) → C(p_z) (76%)	381	9.7
	317	HOMO → L+1 (56.6)	C(p_z) (74%) → C(p_z) (88%)	317	0.1
	279	H–1 → L+1 (47.7)	C(p_z) (82%) → C(p_z) (88%)	274	1.9
	271	H–1 → LUMO (46.6)	C(p_z) (82%) → C(p_z) (76%)		
	244	H–3 → LUMO (52.9)	C(p_z) (54%) → C(p_z) (76%)	242	1.0

5MPO	352	HOMO → LUMO (68.3)	C(p_z) (82%) → C(p_z) (75%)	342	2.8
	316	HOMO → L+1 (57.4)	C(p_z) (82%) → C(p_z) (89%)		
	272	H–1 → LUMO (50.8)	C(p_z) (90%) → C(p_z) (75%)	277	1.9
	248	H–3 → LUMO (60.2)	O(p_z) (37%) → C(p_z) (75%)	247	0.2
6MPO	345	HOMO → LUMO (67.2)	C(p_z) (82%) → C(p_z) (75%)	342	0.9
	315	HOMO → L+1 (56.6)	C(p_z) (82%) → C(p_z) (88%)		
	274	H–1 → L+1 (42.4)	C(p_z) (87%) → C(p_z) (88%)	281	2.5
	244	H–3 → LUMO (52.0)	O(p_z) (35%) → C(p_z) (75%)	248	1.7
5NPO	355	HOMO → LUMO (67.5)	C(p_z) (69%) → C(p_z) (53%)	345	3.0
	299	HOMO → L+1 (60.6)	C(p_z) (69%) → C(p_z) (72%)	288	3.7
	272	H–4 → LUMO (47.1)	C(p_z) (59%) → C(p_z) (53%)		
	260	H–1 → L+1 (48.4)	C(p_z) (89%) → C(p_z) (72%)	251	3.7
6NPO	353	HOMO → LUMO (66.2)	C(p_z) (72%) → C(p_z) (50%) + NO_3 (48%)	381	7.3
	311	HOMO → L+1 (50.7)	C(p_z) (72%) → C(p_z) (72%)		
	273	H–4 → L+1 (47.8)	NO_3 (38%) → C(p_z) (72%)		
	266	H–2 → L+1 (48.7)	C(p_z) (85%) → C(p_z) (72%)	265	0.3
